# Unusual Formation of 1,2,4-Oxadiazine Core in Reaction of Amidoximes with Maleic or Fumaric Esters

**DOI:** 10.3390/molecules27217508

**Published:** 2022-11-03

**Authors:** Sofia I. Presnukhina, Marina V. Tarasenko, Kirill K. Geyl, Svetlana O. Baykova, Sergey V. Baykov, Anton A. Shetnev, Vadim P. Boyarskiy

**Affiliations:** 1 Institute of Chemistry, Saint Petersburg State University, 7/9 Universitetskaya Nab., 199034 Saint Petersburg, Russia; 2 Pharmaceutical Technology Transfer Centre, Yaroslavl State Pedagogical University Named after K.D. Ushinsky, 108 Respublikanskaya St., 150000 Yaroslavl, Russia

**Keywords:** amidoximes, heterocycles, esters, nucleophilic addition, cyclization, basic medium

## Abstract

We have developed a simple and convenient method for the synthesis of 3-aryl- and 3-hetaryl-1,2,4-oxadiazin-5-ones bearing an easily functionalizable (methoxycarbonyl)methyl group at position 6 via the reaction of aryl or hetaryl amidoximes with maleates or fumarates. The conditions for this reaction were optimized. Different products can be synthesized selectively in good yields depending on the base used and the ratio of reactants: substituted (1,2,4-oxadiazin-6-yl)acetic acids, corresponding methyl esters, or hybrid 3-(aryl)-6-((3-(aryl)-1,2,4-oxadiazol-5-yl)methyl)-4*H*-1,2,4-oxadiazin-5(6*H*)-ones. The reaction is tolerant to substituents’ electronic and steric effects in amidoximes. As a result, a series of 2-(5-oxo-3-(*p*-tolyl)-5,6-dihydro-4*H*-1,2,4-oxadiazin-6-yl)acetic acids, their methyl esters, and 1,2,4-oxadiazoles based on them were prepared and characterized by HRMS, ^1^H, and ^13^C NMR spectroscopy. The structures of three of them were elucidated with X-ray diffraction.

## 1. Introduction

**Scheme 1:**
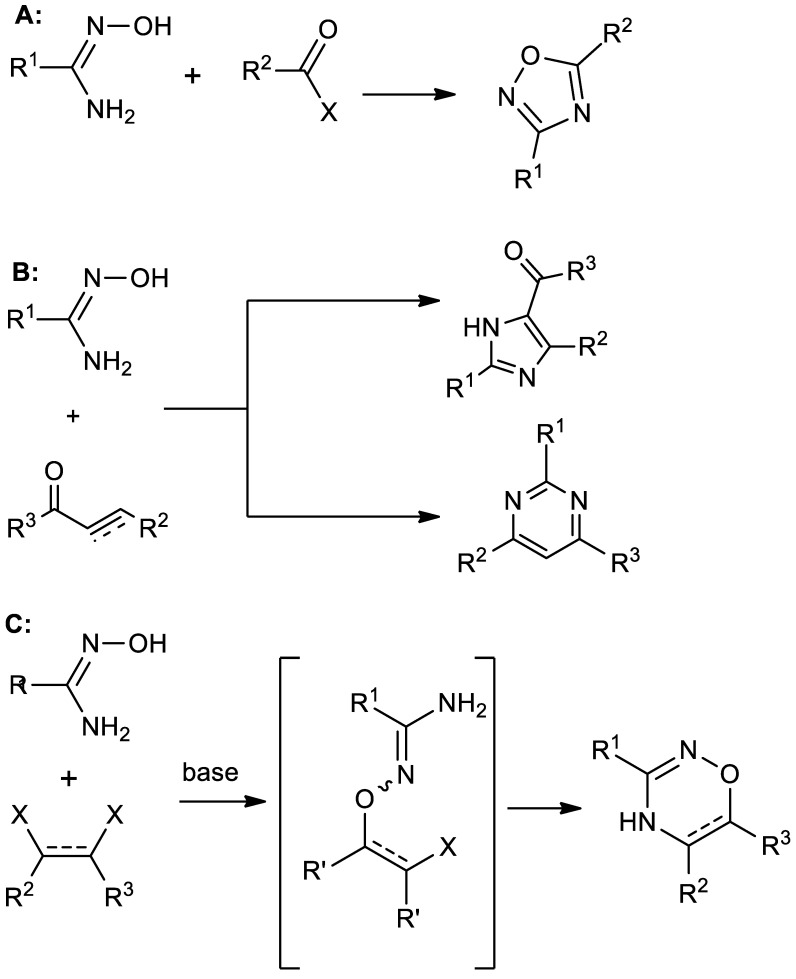
Previously known reactions of amidoximes with carboxylic acid derivatives (**A**), α,β–unsaturated carbonyl compounds (**B**), and vicinal *bis*-electrophiles (**C**).

Amidoximes are polynucleophilic species that are widely used in synthetic chemistry to assemble diverse heterocyclic [1,2,3,4] and organometallic systems [5,6]. The type of the formed heterocyclic core depends mainly on the structure of the electrophile. In particular, the coupling of amidoximes with carbonyl-based compounds such as aldehydes [7,8,9], carboxylic acids [10,11,12], and their derivatives (esters [13,14,15,16], anhydrides [17,18,19,20], as well as acyl chlorides [21,22,23,24]) usually results in 1,2,4-oxadiazoles. In this case, both nucleophilic centers of amidoxime (NOH and NH_2_ groups) attack the same carbon atom of the electrophile (Scheme 1A).

On the other hand, amidoximes react with α,β-unsaturated carbonyl compounds via the Michael addition to the electron-deficient carbon atom of the double bond followed by N-O bond cleavage and the formation of imidazole or pyrimidine core depending on reaction conditions (Scheme 1B), which are harsh in both cases [25].

At the same time, it seems interesting to develop a convenient route for the preparation of 1,2,4-oxadiazines based on such a reaction. These relatively little-studied heterocyclic compounds have significant potential in medicinal chemistry [26,27,28,29,30,31,32,33]. The research in the field of biological activity of this scaffold is hampered, first of all, by the lack of a suitable simple and universal method for its synthesis [34].

In order for amidoximes to be a starting material for the preparation of 1,2,4-oxadiazines, it is necessary that the electrophilic centers in the polyelectrophile occupy a vicinal position (Scheme 1C). For example, the reaction of amidoximes with α-halocarbonyl compounds or their derivatives with the formation of 1,2,4-oxadiazine core has been described [35,36,37]. Other similar examples are reactions with perfluoroarenes [38], acetyleniodonium salts [39], and acetylenedicarboxylates [40].

At first glance, the latter method is quite convenient due to the availability of acetylenedicarboxylates. Unfortunately, this reaction for most substrates resulted in low product yields (not exceeding 50%). The reason for this, most likely, is the excessive reactivity of the intermediate, which contains the fumaric acid fragment. In the basic media, this intermediate is able not only to cyclize to the target oxadiazinone but also to react with nucleophiles present in the system, forming the double bond addition by-products [40].

The use of maleates and fumarates as polyelectrophiles, rather than acetylenedicarboxylates, looks much more attractive. However, the literature provides no examples of the oxadiazine systems synthesis in this way. Although the addition of amidoximes to diethyl chlorofumarate led to the formation of an oxadiazinon, the reaction yield was lower than the one with diethyl acetylenedicarboxylate [40].

We have recently shown [41,42,43] that, in the MOH-DMSO medium, both key stages of the coupling between amidoximes and esters, namely *O*-acylation and 1,2,4-oxadiazole ring closing, occur even at room temperature. We assumed that 1,2,4-oxadiazinones could also be formed in this medium. We have studied this possibility with the example of dimethyl maleate (DMM) and dimethyl fumarate and have shown that this reaction can indeed be a convenient method for the synthesis of 1,2,4-oxadiazinones. Below we present the results of this study.

## 2. Results and Discussion

To start, we evaluated the procedure [43] previously used for the assembly of 1,2,4-oxadiazole core via the condensation of amidoximes with esters (Table 1, entry 1). Although acid **3a** was isolated as the main product instead of the expected ester **4a**, this first experiment encouraged us to conduct a more detailed study. The yield of acid **3a** was only 37%, but the following optimization of DMM and NaOH amounts, as well as the reaction time, provided a 65% yield of the product (Table 1, entries 2 and 4). Increasing the reagent amounts and prolonging the reaction time did not improve the product yield; however, it significantly promoted side processes, mainly the formation of 4-methylbenzonitrile (Table 1, entries 3 and 5). Variation of the base used revealed that NaOH is the most suitable alkali metal hydroxide for the preparation of acids **3**, while the use of *t*-BuONa allows one to obtain corresponding esters 4 (Table 1, entries 6–8). In the latter case, hybrid **5a** bearing two heterocyclic cores (1,2,4-oxadiazinone and 1,2,4-oxadiazole) with the same aromatic substituent (4-methylphenyl) was detected as a minor byproduct (aside from 4-methylbenzonitrile). Probably, its formation can be explained by the reaction of ester **4a** with the second molecule of amidoxime **1a**. After reducing the DMM/amidoxime ratio from 2:1 to 0.4:1, we obtain hybrid **5a** in yield of 82% (Table 1, entry 11). Finally, we carried out the reactions with isomeric dimethyl fumarate (Table 1, entries 12 and 13) and found no significant difference in the reactivity of the two esters (fumarate and maleate).

**Scheme 2:**
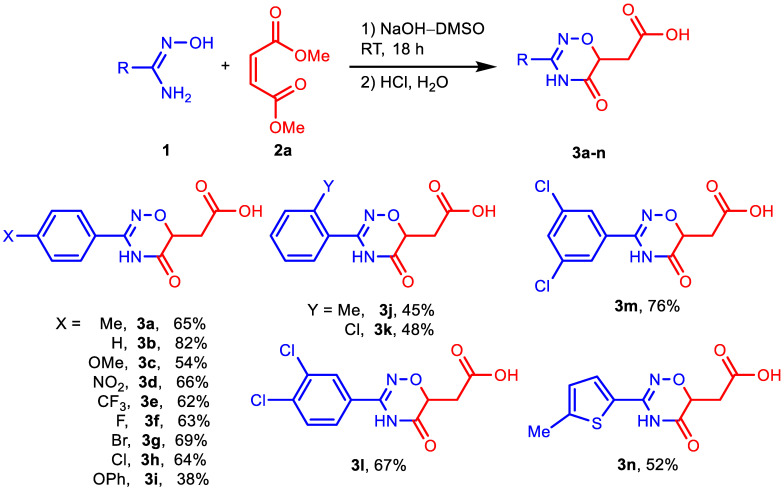
Reaction of amidoximes **1** with **2a** in NaOH–DMSO medium. Reaction conditions: **1** (2 mmol), **2** (4 mmol), NaOH (4 mmol), DMSO (3 mL), RT, 18 h. Isolated yields are presented.

**Scheme 3:**
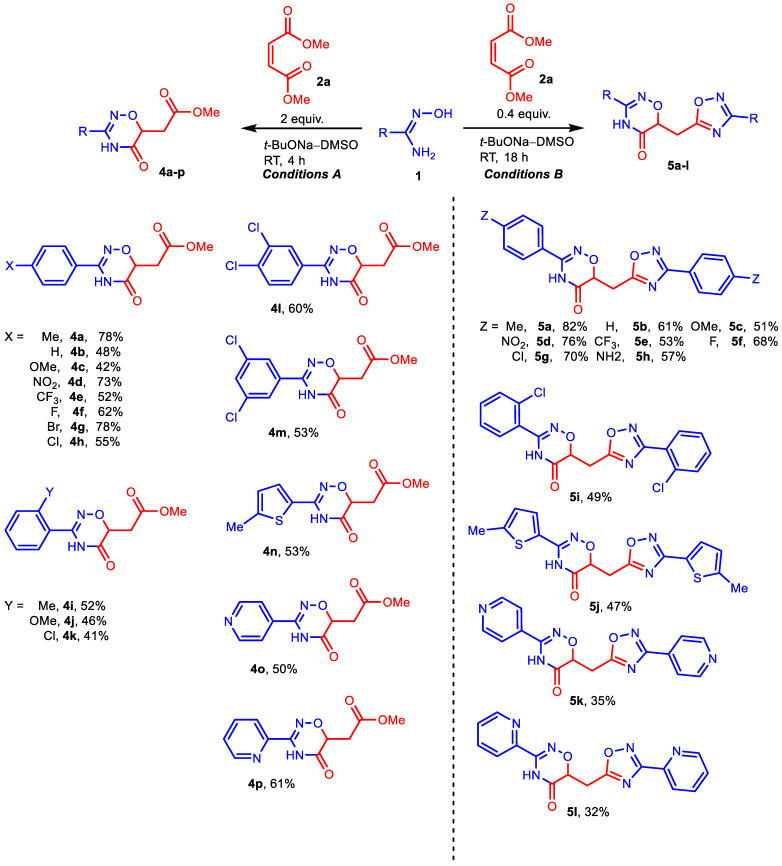
Reactions of amidoximes **1** with **2a** in *t*-BuONa–DMSO medium. Reaction conditions: (**left**) **1** (2 mmol), **2** (4 mmol), *t*-BuONa (2 mmol), DMSO (3 mL), RT, 4 h; (**right**) **1** (5 mmol), **2** (2 mmol), *t*-BuONa (4 mmol), DMSO (3 mL), RT, 18 h. Isolated yields are presented.

Next, we investigated the scope of amidoximes for the synthesis of acids **3** (Scheme 2), esters **4**, and hybrids **5** (Scheme 3). Electron-withdrawing substituents at position 4 of amidoximes have almost no effect on the yield of the corresponding products either in NaOH–DMSO medium or in *t*-BuONa–DMSO medium. The presence of electron donor groups OMe and OPh in amidoximes leads to some decrease in the yields of acids (**3c**, **3i**) or esters (**4c**), but this effect is small. In the synthesis of hybrids **5**, neither the methoxy group nor even the amino group reduced the yields of the desired products (**5c**, **5h**). Substituents in the *ortho*-position also do not interfere with the reaction, although they slightly reduce the yield of the corresponding products (**3j**, **3k**, **4i**, **4k**, **5i**) compared to their *para*-substituted isomers.

The use of heterocyclic amidoximes made it possible to obtain 3-hetaryl-substituted derivatives of 1,2,4-oxadiazin-5-ones in acceptable yields. At the same time, for 5-methyl-thiazol-2-yl and pyridin-4-yl substituents, the corresponding hybrids **5** are formed as well as acids **3** or esters **4**. For pyridin-2-yl amidoxime, the yield of hybrid **5k** is noticeably lower than that of ester **4p**. Nevertheless, we managed to isolate the corresponding hybrid, which confirms the fundamental possibility of using the reaction we discovered for the synthesis of 1,2,4-oxadiazinones bearing additional heterocyclic cores in this case as well.

**Figure 1:**
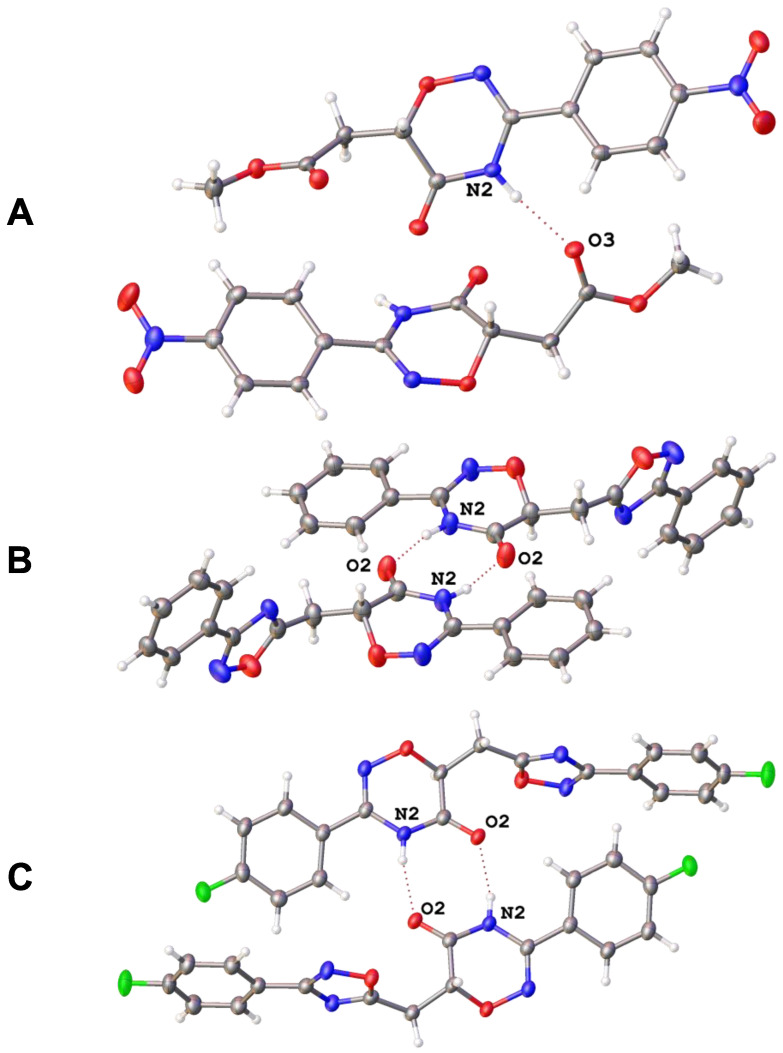
Hydrogen-bonded dimers in crystals of **4d** (**A**), **5b** (**B**), and **5f** (**C**).

To additionally confirm the structures of the obtained products, we used X-ray diffraction. Monocrystalline samples were grown for compounds **4d**, **5b**, and **5f**, and their structures were established by XRD (the structures solution and refinement details, as well as numbering plots presented in Supplementary Materials, Table S1 and Figures S1–S3). In cases of hybrids **5b** and **5f**, molecules in the crystals form dimers via N-H**···O** hydrogen bonds between two oxadiazinone moieties (Figure 1B,C), whereas in the structure of ester **4d**, the acidic NH proton of the oxadiazinone rings is involved in hydrogen bonds with oxygen atoms of methoxy carbonyl groups (Figure 1A). Moreover, π**···**π stacking between aromatic rings was observed in each case.

**Scheme 4:**
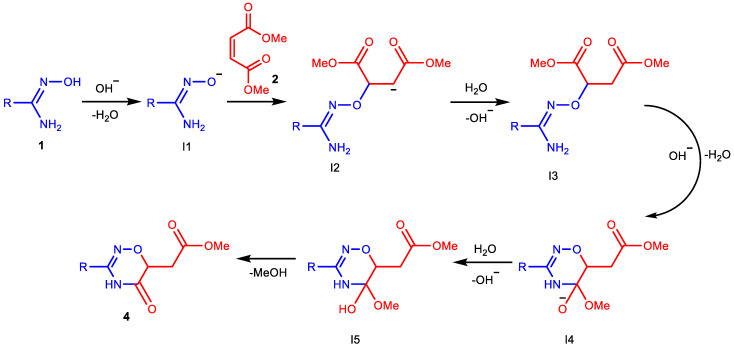
Plausible mechanism of 1,2,4-oxadiazine ring formation.

Based on the literature data, we can suggest the following possible mechanism for the formation of oxadiazines (Scheme 4). It is well known that amidoximes deprotonate in basic media and attack (by the oxygen atoms) double bonds activated by electron-withdrawing substituents [1,40,44]. In our case, intermediate I3 is obtained in this way. Then, in the basic media, the amino group reacts with alkoxycarbonyl group [45], and the cyclization occurs to intermediate I4, which, after protonation and elimination of methanol, forms the target product, similar to the reaction described in [37].

## 3. Material and Methods

### 3.1. General

Amidoximes **1** were prepared from commercial nitriles according to the literature procedures [10,17,46]. Maleic and fumaric esters, as well as all other reagents and solvents, were purchased from Merck (Merck KGaA, Darmstadt, Germany) and used as is. Reactions were monitored by analytical thin layer chromatography (TLC) Macherey-Nagel, TLC plates Silufol UV-254 using UV light for detection. Column chromatography was carried out with silica gel grade 60 (0.040–0.063 mm) 230–400. NMR spectra were recorded on Bruker Avance DPX 400 (400 MHz, 101 MHz, and 376 MHz for ^1^H, ^13^C, and ^19^F, respectively) or on Bruker Avance III 500 MHz (500 MHz for ^1^H, 126 MHz for ^13^C) in DMSO-*d*_6_, CDCl_3_, or acetone-*d*_6_. Chemical shifts are reported as parts per million (*δ*, ppm). The ^1^H and ^13^C spectra were calibrated using the residual signals of nondeuterated solvents as internal reference (2.50 ppm for residual ^1^H, 39.50 ppm for ^13^C in DMSO-*d*_6_, 2.05 ppm for residual ^1^H, 29.84 and 206.26 for residual ^13^C in acetone-*d*_6_; 7.26 ppm for residual ^1^H, 77.16 ppm for ^13^C in CDCl_3_). ^19^F NMR spectra were referenced through the solvent lock (2H) signal according to IUPAC recommended secondary referencing method and the manufacturer’s protocols, and the chemical shifts are reported relative to CFCl_3_ (δ 0.0 ppm). Multiplicities are abbreviated as follows: s = singlet, d = doublet, t = triplet, q = quartet, m = multiplet, br = broad; coupling constants, *J*, are reported in Hertz (Hz). Melting points were determined in open capillary tubes on Electrothermal IA 9300 series Digital Melting Point Apparatus (Calibre Scientific, LA, CA, USA). High-resolution mass spectra (HRMS) were measured on Bruker Maxis HRMS-ESI-qTOF (ESI Ionization) (Bruker, Billerica, MA, USA).

Singe crystals for X-ray studying were obtained by slow evaporation of DMSO solutions of corresponding oxadiazines **4d**, **5b**, and **5f** at RT in air. X-ray diffraction data were collected at an Xcalibur Eos (**4d**) (Agilent Technologies, Santa Clara, CA, USA), at a Rigaku SuperNova (**5f**), and at a Rigaku XtaLAB Synergy-S (**5b**) (Rigaku Corporation, Tokyo, Japan) diffractometers using MoKα (λ = 0.71073 nm) or CuKα (λ = 0.154184 nm) radiation. The structures have been solved with the ShelXT [47] structure solution program using Intrinsic Phasing and refined with the ShelXL [48] refinement package incorporated in the OLEX2 program package [49] using Least Squares minimization. Empirical absorption correction was applied in CrysAlisPro [50] program complex using spherical harmonics, implemented in SCALE3 ABSPACK scaling algorithm. Supplementary crystallographic data for this paper have been deposited at Cambridge Crystallographic Data Centre and can be obtained free of charge via www.ccdc.cam.ac.uk/data_request/cif (accessed on 30 October 2022). CCDC numbers 2210873 (**4d**), 2210875 (**5b**), and 2210876 (**5f**).

### 3.2. Oxadiazinones Preparation and Characterization

**General procedure for preparation acids 3**. To a solution of amidoxime **1** (2 mmol) and ester **2** (4 mmol) in DMSO (3 mL), powdered NaOH (240 mg, 4 mmol) was rapidly added. The reaction mixture was stirred at room temperature for 18 h, diluted with cold brine (30 mL), and twice washed with toluene (5 mL). The water solution was acidified to pH of about 1 by hydrochloric acid and cooled to 5 °C. The resulting precipitate was filtered off, washed with cold water (5 mL), and dried in air at 50 °C.

**2-(5-Oxo-3-(4-methylphenyl)-5,6-dihydro-4*H*-1,2,4-oxadiazin-6-yl)acetic acid 3a.** White powder; 65% yield (161 mg); m.p. 198–199 °C. ^1^H NMR (400 MHz, DMSO-*d*_6_): *δ* 12.55 (s, 1H), 11.33 (s, 1H), 7.64 (d, *J* = 8.3 Hz, 2H), 7.29 (d, *J* = 8.0 Hz, 2H), 4.54 (dd, *J* = 7.7, 4.5 Hz, 1H), 2.92 (dd, *J* = 16.8, 4.5 Hz, 1H), 2.66 (dd, *J* = 16.8, 7.7 Hz, 1H), 2.36 (s, 3H). ^13^C NMR (101 MHz, DMSO-*d*_6_): *δ* 171.4, 167.3, 152.4, 141.8, 129.8, 127.3, 126.8, 73.1, 33.7, 21.6. HRMS (ESI), *m*/*z*: [M + Na]^+^ calcd. for C_12_H_12_N_2_NaO_4_ 271.0689; found 271.0703.

**2-(5-Oxo-3-phenyl-5,6-dihydro-4*H*-1,2,4-oxadiazin-6-yl)acetic acid 3b**. Beige powder; 82% yield (192 mg); m.p. 179–181 °C. ^1^H NMR (400 MHz, DMSO-*d*_6_): *δ* 12.63 (s, 1H), 11.41 (s, 1H), 7.76 (d, *J* = 7.3 Hz, 2H), 7.58–7.43 (m, 3H), 4.58 (dd, *J* = 7.6, 4.5 Hz, 1H), 2.94 (dd, *J* = 16.8, 4.5 Hz, 1H), 2.68 (dd, *J* = 16.8, 7.7 Hz, 1H). ^13^C NMR (101 MHz, CDCl_3_): *δ* 171.2, 167.0, 152.3, 131.6, 129.5, 129.1, 127.2, 72.9, 33.5. HRMS (ESI), *m*/*z*: [M + Na]^+^ calcd. for C_11_H_10_N_2_NaO_4_ 257.0533; found 257.0538.

**2-(3-(4-Methoxyphenyl)-5-oxo-5,6-dihydro-4*H*-1,2,4-oxadiazin-6-yl)acetic acid 3c.** Beige powder; 54% yield (143 mg); m.p. 202–204 °C. ^1^H NMR (400 MHz, DMSO-*d*_6_): *δ* 12.58 (s, 1H), 11.31 (s, 1H), 7.71 (d, *J* = 8.5 Hz, 2H), 7.04 (d, *J* = 8.5 Hz, 2H), 4.54 (dd, *J* = 7.1, 4.5 Hz, 1H), 3.82 (s, 3H), 2.93 (dd, *J* = 16.7, 4.1 Hz, 1H), 2.66 (dd, *J* = 16.7, 7.7 Hz, 1H). ^13^C NMR (101 MHz, CDCl_3_): *δ* 171.3, 167.2, 162.0, 152.0, 128.8, 121.5, 114.5, 72.9, 55.9, 33.5. HRMS (ESI), m/z: [M + Na]^+^ calcd. for C_12_H_12_N_2_NaO_5_ 287.0638; found 287.0644.

**2-(3-(4-Nitrophenyl)-5-oxo-5,6-dihydro-4*H*-1,2,4-oxadiazin-6-yl)acetic acid 3d.** Yellow powder; 66% yield (184 mg); m.p. 210–213 °C. ^1^H NMR (400 MHz, DMSO-*d*_6_): *δ* 1H NMR (400 MHz, DMSO) δ 12.13 (s, 1H), 8.33 (d, *J* = 8.9 Hz, 2H), 8.03 (d, *J* = 8.9 Hz, 2H), 4.65 (dd, *J* = 7.6, 4.5 Hz, 1H), 2.94 (dd, *J* = 16.8, 4.5 Hz, 1H), 2.68 (dd, *J* = 16.8, 7.6 Hz, 1H). ^13^C NMR (101 MHz, DMSO-*d*_6_): *δ* 171.3, 166.8, 151.0, 149.4, 135.5, 128.7, 124.2, 73.2, 33.7. HRMS (ESI), *m*/*z*: [M + Na]^+^ calcd. for C_11_H_9_N_3_NaO_6_ 316.0540; found 316.0527.

**2-(5-Oxo-3-(4-(trifluoromethyl)phenyl)-5,6-dihydro-4*H*-1,2,4-oxadiazin-6-yl)acetic acid 4e**. White powder; 62% yield (187 mg); m.p. 218–220 °C. ^1^H NMR (400 MHz, DMSO-*d*_6_): *δ* 12.60 (s, 1H), 11.59 (s, 1H), 7.97 (d, *J* = 8.2 Hz, 2H), 7.87 (d, *J* = 8.2 Hz, 2H), 4.63 (dd, *J* = 7.7, 4.5 Hz, 1H), 2.95 (dd, *J* = 16.8, 4.6 Hz, 1H), 2.70 (dd, *J* = 16.9, 7.7 Hz, 1H). ^13^C NMR (101 MHz, DMSO-*d*_6_): *δ* 171.4, 167.0, 151.5, 133.6, 131.7 (q, *J* = 32.1 Hz), 128.4, 126.2, 124.5 (d, *J* = 272.7 Hz), 73.2, 33.6. ^19^F NMR (376 MHz, DMSO-*d*_6_): *δ* –61.43. HRMS (ESI), *m*/*z*: [M + Na]^+^ calcd. for C_12_H_9_F_3_N_2_NaO_4_ 325.0407; found 325.0409.

**2-(3-(4-Fluorophenyl)-5-oxo-5,6-dihydro-4*H*-1,2,4-oxadiazin-6-yl)acetic acid 3f.** Beige powder; 63% yield (159 mg); m.p. 202–203 °C. ^1^H NMR (400 MHz, DMSO-*d*_6_): *δ* 12.56 (s, 1H), 11.43 (s, 1H), 7.84–7.77 (m, 2H), 7.34 (t, *J* = 8.9 Hz, 2H), 4.57 (dd, *J* = 7.7, 4.5 Hz, 1H), 2.93 (dd, *J* = 16.8, 4.5 Hz, 1H), 2.67 (dd, *J* = 16.8, 7.7 Hz, 1H). ^13^C NMR (101 MHz, DMSO-*d*_6_): *δ* 171.4, 167.2, 164.4 (d, *J* = 248.8 Hz), 151.8, 130.0 (d, *J* = 8.7 Hz), 126.2, 116.4 (d, *J* = 22.1 Hz), 73.1, 33.6. ^19^F NMR (376 MHz, DMSO-*d*_6_): *δ* –109.20. HRMS (ESI), *m*/*z*: [M + Na]^+^ calcd. for C_11_H_9_FN_2_NaO_4_ 275.0439; found 275.0453.

**2-(3-(4-Bromophenyl)-5-oxo-5,6-dihydro-4*H*-1,2,4-oxadiazin-6-yl)acetic acid 3g.** White powder; 69% yield (216 mg); m.p. 246–248 °C. ^1^H NMR (400 MHz, DMSO-*d*_6_): *δ* 12.55 (s, 1H), 11.44 (s, 1H), 7.70 (s, 4H), 4.58 (dd, *J* = 7.7, 4.5 Hz, 1H), 2.93 (dd, *J* = 16.8, 4.5 Hz, 1H), 2.67 (dd, *J* = 16.8, 7.7 Hz, 1H). ^13^C NMR (101 MHz, DMSO-*d*_6_): *δ* 171.4, 167.1, 151.8, 132.3, 129.4, 128.9, 125.4, 73.1, 33.6. HRMS (ESI), *m*/*z*: [M + Na]^+^ calcd. for C_11_H_9_BrN_2_NaO_4_ 334.9638; found 334.9649.

**2-(3-(4-Chlorophenyl)-5-oxo-5,6-dihydro-4*H*-1,2,4-oxadiazin-6-yl)acetic acid 3h.** White powder; 64% yield (172 mg); m.p. 191–193 °C. ^1^H NMR (400 MHz, DMSO-*d*_6_): *δ* 12.38 (br s, 1H), 11.52 (s, 1H), 7.78 (d, *J* = 8.5 Hz, 2H), 7.57 (d, *J* = 8.5 Hz, 2H), 4.59 (dd, *J* = 7.5, 4.5 Hz, 1H), 2.93 (dd, *J* = 16.8, 4.4 Hz, 1H), 2.67 (dd, *J* = 16.8, 7.7 Hz, 1H). ^13^C NMR (101 MHz, DMSO-*d*_6_): *δ* 171.2, 166.9, 151.5, 136.4, 129.2, 129.1, 128.3, 73.0, 33.5. HRMS (ESI), *m*/*z*: [M + H]^+^ calcd. for C_11_H_10_ClN_2_O_4_ 269.0324; found 269.0337.

**2-(5-Oxo-3-(4-phenoxyphenyl)-5,6-dihydro-4*H*-1,2,4-oxadiazin-6-yl)acetic acid 3i.** Beige powder; 38% yield (124 mg); m.p. 179–180 °C. ^1^H NMR (400 MHz, DMSO-*d*_6_): *δ* 12.55 (s, 1H), 11.38 (s, 1H), 7.77 (d, *J* = 8.9 Hz, 2H), 7.46 (t, *J* = 8.0 Hz, 2H), 7.23 (t, *J* = 7.4 Hz, 1H), 7.13–7.03 (m, 4H), 4.56 (dd, *J* = 7.7, 4.5 Hz, 1H), 2.93 (dd, *J* = 16.7, 4.5 Hz, 1H), 2.67 (dd, *J* = 16.8, 7.7 Hz, 1H). ^13^C NMR (101 MHz, DMSO-*d*_6_): *δ* 171.4, 167.2, 160.2, 156.1, 152.0, 130.9, 129.4, 125.1, 124.2, 120.3, 118.4, 73.1, 33.7. HRMS (ESI), *m*/*z*: [M + Na]^+^ calcd. for C_17_H_14_N_2_NaO_5_ 349.0795; found 349.0812.

**2-(5-Oxo-3-(2-methylphenyl)-5,6-dihydro-4*H*-1,2,4-oxadiazin-6-yl)acetic acid 3j.** White powder; 45% yield (112 mg); m.p. 201–202 °C. ^1^H NMR (400 MHz, DMSO-*d*_6_): *δ* 12.60 (s, 1H), 11.21 (s, 1H), 7.46–7.40 (m, 2H), 7.35–7.27 (m, 2H), 4.62 (dd, *J* = 7.8, 4.4 Hz, 1H), 2.94 (dd, *J* = 16.8, 4.4 Hz, 1H), 2.68 (dd, *J* = 16.7, 7.8 Hz, 1H), 2.35 (s, 3H). ^13^C NMR (101 MHz, DMSO-*d*_6_): *δ* 171.42, 166.82, 153.15, 137.37, 131.22, 131.04, 130.06, 126.50, 73.05, 33.71, 20.06. HRMS (ESI), *m*/*z*: [M + Na]^+^ calcd. for C_12_H_12_N_2_NaO_4_ 271.0689; found 271.0704.

**2-(3-(2-Chlorophenyl)-5-oxo-5,6-dihydro-4*H*-1,2,4-oxadiazin-6-yl)acetic acid 3k.** Beige powder; 48% yield (129); m.p. 197–199 °C. ^1^H NMR (400 MHz, DMSO-*d*_6_): *δ* 12.65 (s, 1H), 11.42 (s, 1H), 7.64–7.55 (m, 3H), 7.47 (td, *J* = 7.4, 1.5 Hz, 1H), 4.60 (dd, *J* = 7.8, 4.5 Hz, 1H), 2.94 (dd, *J* = 16.8, 4.6 Hz, 1H), 2.72 (dd, *J* = 16.8, 7.7 Hz, 1H). ^13^C NMR (101 MHz, DMSO-*d*_6_): *δ* 171.16, 166.35, 151.53, 132.82, 132.75, 131.94, 130.23, 129.41, 127.89, 72.98, 33.37. HRMS (ESI), *m*/*z*: [M + H]^+^ calcd. for C_11_H_10_ClN_2_O_4_ 269.0324; found 269.0334.

**2-(3-(3,4-Dichlorophenyl)-5-oxo-5,6-dihydro-4*H*-1,2,4-oxadiazin-6-yl)acetic acid 3l.** White powder; 67% yield (203 mg); m.p. 243–245 °C. ^1^H NMR (400 MHz, DMSO-*d*_6_): *δ* 12.60 (s, 1H), 11.52 (s, 1H), 8.00 (s, 1H), 7.80–7.72 (m, *J* = 8.5, 6.2, 1.9 Hz, 2H), 4.60 (dd, *J* = 7.4, 4.6 Hz, 1H), 2.93 (dd, *J* = 16.8, 4.4 Hz, 1H), 2.69 (dd, *J* = 16.9, 7.6 Hz, 1H). ^13^C NMR (101 MHz, DMSO-*d*_6_): *δ* 171.3, 166.9, 150.8, 134.5, 132.2, 131.6, 130.2, 129.4, 127.6, 73.2, 33.6. HRMS (ESI), *m*/*z*: [M+Na]^+^ calcd. for C_11_H_8_Cl_2_N_2_NaO_4_ 324.9753; found 324.9738.

**2-(3-(3,5-Dichlorophenyl)-5-oxo-5,6-dihydro-4*H*-1,2,4-oxadiazin-6-yl)acetic acid 3m.** White powder; 76% yield (230 mg); m.p. 248–250 °C. ^1^H NMR (400 MHz, DMSO-*d*_6_): *δ* 12.59 (s, 1H), 11.51 (s, 1H), 7.83 (s, 1H), 7.79 (s, 2H), 4.60 (dd, *J* = 7.7, 4.5 Hz, 1H), 2.93 (dd, *J* = 16.9, 4.5 Hz, 1H), 2.69 (dd, *J* = 16.9, 7.7 Hz, 1H). ^13^C NMR (101 MHz, DMSO-*d*_6_): *δ* 171.3, 166.8, 150.5, 135.1, 133.0, 131.1, 126.2, 73.2, 33.6. HRMS (ESI), *m*/*z*: [M + Na]^+^ calcd. for C_11_H_8_Cl_2_N_2_NaO_4_ 324.9753; found 324.9774.

**2-(3-(5-Methylthiophen-2-yl)-5-oxo-5,6-dihydro-4*H*-1,2,4-oxadiazin-6-yl)acetic acid 3n.** White powder; 52% yield (132 mg); m.p. 202–203 °C. ^1^H NMR (400 MHz, DMSO-*d*_6_): *δ* 12.55 (s, 1H), 11.50 (s, 1H), 7.58 (d, *J* = 3.7 Hz, 1H), 6.87 (dd, *J* = 3.6, 0.9 Hz, 1H), 4.57 (dd, *J* = 7.7, 4.4 Hz, 1H), 2.92 (dd, *J* = 16.8, 4.4 Hz, 1H), 2.66 (dd, *J* = 16.8, 7.8 Hz, 1H), 2.47 (s, 3H) ^13^C NMR (101 MHz, DMSO-*d*_6_): *δ* 171.3, 167.0, 148.5, 144.6, 129.8, 128.8, 126.8, 73.5, 33.6, 15.7. HRMS (ESI), *m*/*z*: [M + Na]^+^ calcd. for C_10_H_10_N_2_NaO_4_S 277.0253; found 277.0244.

**General procedure for preparation esters 4**. To a solution of amidoxime **1** (2 mmol) in DMSO (3 mL), *t*-BuONa (192 mg, 2 mmol) was rapidly added. The reaction mixture was stirred at room temperature for 10 min, and ester **2** (4 mmol) was added. The reaction mixture was stirred at room temperature for another 4 h and was diluted with cold brine (30 mL). Compounds **4a**, **4d**, **4e**, **4g**, and **4l**–**p** were filtered off, washed with cold water (5 mL) or 2), and dried in air at 50 °C. Compounds **4b**, **4c**, **4f**, **4h**–**k** were extracted with toluene (5 × 3 mL) and dried under reducing pressure. If necessary, the crude product was purified by column chromatography on SiO_2_ using EA:Hexane:MeOH mixture as an eluent.

**Methyl 2-(3-(4-methylphenyl)-5-oxo-5,6-dihydro-4*H*-1,2,4-oxadiazin-6-yl)acetate 4a**. White powder; 78% yield (205 mg); m.p. 158–160 °C. ^1^H NMR (400 MHz, CDCl_3_): *δ* 8.58 (s, 1H), 7.59 (d, *J* = 7.9 Hz, 2H), 7.28 (d, *J* = 7.9 Hz, 2H), 4.76–4.71 (m, 1H), 3.74 (s, 3H), 3.10 (dd, *J* = 16.9, 5.0 Hz, 1H), 2.89 (dd, *J* = 16.8, 7.2 Hz, 1H), 2.40 (s, 3H). ^13^C NMR (101 MHz, CDCl_3_): *δ* 170.0, 166.6, 151.1, 142.6, 130.1, 126.1, 125.8, 73.2, 52.5, 33.4, 21.7. HRMS (ESI), *m*/*z*: [M + Na]^+^ calcd. for C_13_H_14_N_2_NaO_4_ 285.0846; found 285.0845.

**Methyl 2-(5-oxo-3-phenyl-5,6-dihydro-4*H*-1,2,4-oxadiazin-6-yl)acetate 4b**. White powder; 48% yield (119 mg); m.p. 124–126 °C. ^1^H NMR (400 MHz, CDCl_3_): *δ* 8.92 (s, 1H), 7.72 (d, *J* = 7.2 Hz, 2H), 7.56–7.44 (m, 3H), 4.75 (dd, *J* = 7.1, 5.1 Hz, 1H), 3.73 (s, 3H), 3.10 (dd, *J* = 16.8, 5.1 Hz, 1H), 2.90 (dd, *J* = 16.8, 7.2 Hz, 1H). ^13^C NMR (101 MHz, CDCl_3_): *δ* 170.5, 167.0, 152.5, 131.9, 129.6, 129.3, 127.4, 72.8, 52.5, 33.4. HRMS (ESI), *m*/*z*: [M + Na]^+^ calcd. for C_12_H_12_N_2_NaO_4_ 271.0689; found 271.0681.

**Methyl 2-(3-(4-methoxyphenyl)-5-oxo-5,6-dihydro-4*H*-1,2,4-oxadiazin-6-yl)acetate 4c**. White powder; 42% yield (117 mg); m.p. 161–163 °C. ^1^H NMR (400 MHz, CDCl_3_): *δ* 10.05 (s, 1H), 8.74 (d, *J* = 5.1 Hz, 2H), 7.67 (d, *J* = 5.8 Hz, 2H), 4.79–4.73 (m, 1H), 3.74 (s, 3H), 3.11 (dd, *J* = 16.9, 5.3 Hz, 1H), 2.93 (dd, *J* = 16.9, 6.8 Hz, 1H). ^13^C NMR (126 MHz, CDCl_3_): *δ* 169.9, 167.1, 162.4, 150.9, 127.7, 120.5, 114.5, 72.8, 55.5, 52.3, 33.2. HRMS (ESI), *m*/*z*: [M + Na]^+^ calcd. for C_13_H_14_N_2_NaO_5_ 301.0795; found 301.0794.

**Methyl 2-(3-(4-nitrophenyl)-5-oxo-5,6-dihydro-4*H*-1,2,4-oxadiazin-6-yl)acetate 4d.** Yellow powder; 73% yield (214 mg); m.p. 112–113 °C. ^1^H NMR (400 MHz, DMSO-*d*_6_): *δ* 11.71 (s, 1H), 8.34 (d, *J* = 8.6 Hz, 2H), 8.03 (d, *J* = 8.8 Hz, 2H), 4.71 (dd, *J* = 7.7, 4.5 Hz, 1H), 3.67 (s, 3H), 3.07 (dd, *J* = 16.9, 4.6 Hz, 1H), 2.85 (dd, *J* = 16.8, 7.7 Hz, 1H). ^13^C NMR (101 MHz, DMSO-*d*_6_): *δ* 170.2, 166.5, 151.1, 149.4, 135.4, 128.7, 124.2, 72.8, 52.3, 33.1. HRMS (ESI), *m*/*z*: [M + Na]^+^ calcd. for C_12_H_11_N_3_NaO_6_ 316.0540; found 316.0527.

**Methyl 2-(5-oxo-3-(4-(trifluoromethyl)phenyl)-5,6-dihydro-4*H*-1,2,4-oxadiazin-6-yl)acetate 4e.** White powder; 52% yield (164 mg); m.p. 172–173 °C. ^1^H NMR (400 MHz, CDCl_3_): *δ* 9.65 (s, 1H), 7.90 (d, *J* = 8.2 Hz, 2H), 7.73 (d, *J* = 8.1 Hz, 2H), 4.79–4.73 (m, 1H), 3.09 (dd, *J* = 16.8, 5.4 Hz, 1H), 2.92 (dd, *J* = 16.8, 6.8 Hz, 1H). ^13^C NMR (101 MHz, CDCl_3_): *δ* 169.8, 167.3, 150.0, 133.7 (d, *J* = 33.1 Hz), 132.0, 126.8, 126.3 (q, *J* = 3.5 Hz), 123.7 (d, *J* = 272.8 Hz), 73.0, 52.5, 33.3. ^19^F NMR (376 MHz, CDCl_3_): *δ* –63.13. HRMS (ESI), *m*/*z*: [M + Na]^+^ calcd. for C_13_H_11_F_3_N_2_NaO_4_ 339.0563; found 339.0558.

**Methyl 2-(3-(4-fluorophenyl)-5-oxo-5,6-dihydro-4*H*-1,2,4-oxadiazin-6-yl)acetate 4f.** White powder; 62% yield (165 mg); m.p. 165–166 °C. ^1^H NMR (400 MHz, CDCl_3_): *δ* 9.06 (s, 1H), 7.74 (dd, *J* = 8.7, 5.1 Hz, 2H), 7.19–7.12 (m, 2H), 4.75–4.71 (m, 1H), 3.73 (s, 3H), 3.09 (dd, *J* = 16.9, 5.2 Hz, 1H), 2.89 (dd, *J* = 16.8, 7.0 Hz, 1H). ^13^C NMR (101 MHz, DMSO-*d*_6_): *δ* 170.4, 166.9, 152.8, 151.9, 132.3, 129.5, 127.1 (d, *J* = 335.2 Hz), 72.8, 52.5, 33.3. ^19^F NMR (376 MHz, CDCl_3_): *δ* –107.05. HRMS (ESI), *m*/*z*: [M + Na]^+^ calcd. for C_12_H_11_FN_2_NaO_4_ 289.0595; found 289.0584.

**Methyl 2-(3-(4-bromophenyl)-5-oxo-5,6-dihydro-4*H*-1,2,4-oxadiazin-6-yl)acetate 4g.** White powder; 78% yield (255 mg); m.p. 146–147 °C. ^1^H NMR (400 MHz, CDCl_3_): *δ* 9.09 (s, 1H), 7.61 (s, 4H), 4.74 (dd, *J* = 7.0, 5.2 Hz, 1H), 3.74 (s, 3H), 3.09 (dd, *J* = 16.8, 5.2 Hz, 1H), 2.90 (dd, *J* = 16.8, 7.0 Hz, 1H). ^13^C NMR (101 MHz, DMSO-*d*_6_): *δ* 170.4, 166.9, 151.9, 132.3, 129.5, 128.8, 125.5, 72.8, 52.5, 33.3. HRMS (ESI), *m*/*z*: [M + Na]^+^ calcd. for C_12_H_11_BrN_2_NaO_4_ 348.9794; found 348.9778.

**Methyl 2-(3-(4-chlorophenyl)-5-oxo-5,6-dihydro-4*H*-1,2,4-oxadiazin-6-yl)acetate 4h.** White powder; 55% yield (155 mg); m.p. 168–170 °C. ^1^H NMR (400 MHz, CDCl_3_): *δ* 9.21 (s, 1H), 7.68 (d, *J* = 8.6 Hz, 2H), 7.44 (d, *J* = 8.6 Hz, 2H), 4.73 (dd, *J* = 6.9, 5.2 Hz, 1H), 3.73 (s, 3H), 3.09 (dd, *J* = 16.8, 5.2 Hz, 1H), 2.90 (dd, *J* = 16.8, 7.0 Hz, 1H). ^13^C NMR (101 MHz, CDCl_3_): *δ* 170.5, 167.0, 151.9, 136.6, 129.4, 129.3, 128.5, 72.84, 52.5, 33.3. HRMS (ESI), *m*/*z*: [M + Na]^+^ calcd. for C_12_H_11_ClN_2_NaO_4_ 305.0300; found 305.0280.

**Methyl 2-(3-(2-methylphenyl)-5-oxo-5,6-dihydro-4*H*-1,2,4-oxadiazin-6-yl)acetate 4i.** White powder; 52% yield (136 mg); m.p. 115–117 °C. ^1^H NMR (400 MHz, CDCl_3_): *δ* 9.09 (s, 1H), 7.47–7.27 (m, 2H), 7.38–7.30 (m, 2H), 4.73 (dd, *J* = 7.2, 5.1 Hz, 1H), 3.72 (d, *J* = 1.3 Hz, 3H), 3.08 (dd, *J* = 16.9, 4.9 Hz, 1H), 2.88 (dd, *J* = 16.8, 7.1 Hz, 1H), 2.39 (s, 3H).^13^C NMR (101 MHz, DMSO-*d*_6_): *δ* 170.5, 167.0, 152.6, 138.6, 132.5, 129.5, 129.2, 127.9, 124.6, 72.8, 52.5, 33.4, 21.5. HRMS (ESI), *m*/*z*: [M + Na]^+^ calcd. for C_13_H_14_N_2_NaO_4_ 285.0846; found 285.0859.

**Methyl 2-(3-(2-methoxyphenyl)-5-oxo-5,6-dihydro-4*H*-1,2,4-oxadiazin-6-yl)acetate 4j.** White powder; 46% yield (128 mg); m.p. 110–112 °C. ^1^H NMR (400 MHz, CDCl_3_): *δ* 9.08 (s, 1H), 7.36 (t, *J* = 7.9 Hz, 1H), 7.30–7.25 (m, 2H), 7.05 (dd, *J* = 8.2, 2.4 Hz, 1H), 4.73 (dd, *J* = 7.2, 5.0 Hz, 1H), 3.83 (s, 3H), 3.73 (s, 3H), 3.08 (dd, *J* = 16.8, 5.0 Hz, 1H), 2.88 (dd, *J* = 16.8, 7.1 Hz, 1H). ^13^C NMR (126 MHz, CDCl_3_): *δ* 169.8, 166.6, 160.1, 150.8, 130.2, 129.7, 118.3, 118.1, 111.0, 72.9, 55.5, 52.3, 33.1. HRMS (ESI), *m*/*z*: [M + Na]^+^ calcd. for C_13_H_14_N_2_NaO_5_ 301.0795; found 301.0802.

**Methyl 2-(3-(2-chlorophenyl)-5-oxo-5,6-dihydro-4*H*-1,2,4-oxadiazin-6-yl)acetate 4k.** Light-brown powder; 41% yield (116 mg); m.p. 91–92 °C. ^1^H NMR (400 MHz, CDCl_3_): *δ* 8.76 (s, 1H), 7.56 (d, *J* = 7.6 Hz, 1H), 7.44 (d, *J* = 3.4 Hz, 2H), 7.37–7.32 (m, 1H), 4.72 (dd, *J* = 7.5, 4.8 Hz, 1H), 3.71 (s, 3H), 3.03 (dd, *J* = 16.7, 4.8 Hz, 1H), 2.86 (dd, *J* = 16.8, 7.5 Hz, 1H). ^13^C NMR (126 MHz, CDCl_3_): *δ* 169.8, 165.7, 150.6, 132.6, 132.6, 131.5, 130.5, 128.0, 127.5, 72.9, 52.4, 33.1. HRMS (ESI), *m*/*z*: [M + Na]^+^ calcd. for C_12_H_11_ClN_2_NaO_4_ 305.0300; found 305.0274.

**Methyl 2-(3-(3,4-dichlorophenyl)-5-oxo-5,6-dihydro-4*H*-1,2,4-oxadiazin-6-yl)acetate 4l.** White powder; 60% yield (190 mg); m.p. 163–165 °C. ^1^H NMR (400 MHz, CDCl_3_): *δ* 9.21 (s, 1H), 7.87 (s, 1H), 7.60–7.53 (m, 2H), 4.74 (dd, *J* = 6.9, 5.0 Hz, 1H), 3.74 (s, 3H), 3.11 (dd, *J* = 16.9, 5.1 Hz, 1H), 2.92 (dd, *J* = 16.9, 6.9 Hz, 1H). ^13^C NMR (101 MHz, DMSO-*d*_6_): *δ* 170.4, 166.8, 150.9, 134.6, 132.2, 131.6, 130.2, 129.4, 127.6, 72.9, 52.5, 33.3. HRMS (ESI), *m*/*z*: [M + Na]^+^ calcd. for C_12_H_10_Cl_2_N_2_NaO_4_ 338.9910; found 338.9909.

**Methyl 2-(3-(3,5-dichlorophenyl)-5-oxo-5,6-dihydro-4*H*-1,2,4-oxadiazin-6-yl)acetate 4m**. White powder; 53% yield (168 mg); m.p. 145–147 °C. ^1^H NMR (400 MHz, CDCl_3_): *δ* 9.41 (s, 1H), 7.67 (s, 2H), 7.50 (s, 1H), 4.75–4.70 (m, 1H), 3.74 (s, 3H), 3.13 (dd, *J* = 16.9, 3.6 Hz, 1H), 2.93 (dd, *J* = 17.1, 6.9 Hz, 1H). ^13^C NMR (101 MHz, CDCl_3_): *δ* 169.9, 167.3, 149.0, 136.2, 131.8, 131.4, 124.8, 73.0, 52.6, 33.2. HRMS (ESI), *m*/*z*: [M + Na]^+^ calcd. for C_12_H_10_Cl_2_N_2_NaO_4_ 338.9910; found 338.9887.

**Methyl 2-(3-(5-methylthiophen-2-yl)-5-oxo-5,6-dihydro-4*H*-1,2,4-oxadiazin-6-yl)acetate 4n**. White powder; 53% yield (142 mg); m.p. 168–170 °C. ^1^H NMR (400 MHz, CDCl_3_): *δ* 9.69 (s, 1H), 7.32 (d, *J* = 3.7 Hz, 1H), 6.75 (s, 1H), 4.77–4.71 (m, 1H), 3.73 (s, 3H), 3.07 (dd, *J* = 16.7, 5.2 Hz, 1H), 2.88 (dd, *J* = 16.6, 7.2 Hz, 1H), 2.49 (s, 3H). ^13^C NMR (126 MHz, CDCl_3_): *δ* 169.7, 167.2, 147.4, 145.6, 127.9, 127.8, 126.0, 73.1, 52.3, 33.2, 15.6. HRMS (ESI), *m*/*z*: [M + Na]^+^ calcd. for C_11_H_12_N_2_NaO_4_S 291.0410; found 291.0390.

**Methyl 2-(5-oxo-3-(pyridin-4-yl)-5,6-dihydro-4*H*-1,2,4-oxadiazin-6-yl)acetate 4o**. White powder; 50% yield (125 mg); m.p. 145–147 °C. ^1^H NMR (400 MHz, CDCl_3_): *δ* 10.05 (s, 1H), 8.74 (d, *J* = 5.0 Hz, 2H), 7.67 (d, *J* = 5.7 Hz, 2H), 4.79–4.73 (m, 1H), 3.74 (s, 3H), 3.11 (dd, *J* = 16.9, 5.3 Hz, 1H), 2.93 (dd, *J* = 16.9, 6.8 Hz, 1H). ^13^C NMR (126 MHz, CDCl_3_): *δ* 169.7, 166.8, 150.6, 149.1, 136.4, 120.0, 72.9, 52.4, 33.1. HRMS (ESI), *m*/*z*: [M + Na]^+^ calcd. for C_11_H_11_N_3_NaO_4_ 272.0642; found 272.0639.

**Methyl 2-(5-oxo-3-(pyridin-2-yl)-5,6-dihydro-4*H*-1,2,4-oxadiazin-6-yl)acetate 4p.** White powder; 61% yield (152 mg); m.p. 119–120 °C. ^1^H NMR (400 MHz, CDCl_3_): *δ* 9.42 (s, 1H), 8.58 (d, *J* = 4.5 Hz, 1H), 8.08 (d, *J* = 7.9 Hz, 1H), 7.81 (td, *J* = 7.7, 1.6 Hz, 1H), 7.45–7.40 (m, 1H), 4.75 (dd, *J* = 7.6, 4.4 Hz, 1H), 3.75 (s, 3H), 3.15 (dd, *J* = 16.9, 4.5 Hz, 1H), 2.91 (dd, *J* = 16.9, 7.6 Hz, 1H). ^13^C NMR (101 MHz, CDCl_3_): *δ* 170.0, 165.1, 148.8, 145.3, 137.7, 126.3, 121.1, 73.4, 52.5, 40.5, 33.6. HRMS (ESI), *m*/*z*: [M + Na]^+^ calcd. for C_11_H_11_N_3_NaO_4_ 272.0642; found 272.0656.

**General procedure for preparation of hybrids 5.** To a solution of amidoxime **1** (5 mmol) and ester **2** (2 mmol) in DMSO (3 mL), *t*-BuONa (384 mg, 4 mmol) was rapidly added. The reaction mixture was stirred at room temperature for 18 h and diluted with cold brine (30 mL). The resulting precipitate was filtered off, washed with cold water (5 mL), and dried in air at 50 °C. If necessary, the crude product was purified by column chromatography on SiO_2_ using EA:Hexane:MeOH mixture as an eluent.

**3-(4-Methylphenyl)-6-((3-(4-methylphenyl)-1,2,4-oxadiazol-5-yl)methyl)-4*H*-1,2,4-oxadiazin-5(6*H*)-one 5a.** Beige powder; 82% yield (297 mg); m.p. 189–190 °C. ^1^H NMR (400 MHz, Acetone-*d*_6_): *δ* 10.26 (s, 1H), 7.97 (d, *J* = 8.0 Hz, 2H), 7.76 (d, *J* = 8.0 Hz, 2H), 7.37 (d, *J* = 8.0 Hz, 2H), 7.32 (d, *J* = 7.9 Hz, 2H), 4.98 (dd, *J* = 8.2, 4.6 Hz, 1H), 3.80 (dd, *J* = 16.4, 4.5 Hz, 1H), 3.55 (dd, *J* = 16.4, 8.1 Hz, 1H), 2.42 (s, 3H), 2.40 (s, 3H). ^13^C NMR (101 MHz, Acetone-*d*_6_): *δ* 176.5, 168.2, 165.4, 152.0, 141.7, 141.6, 129.6, 129.3, 127.1, 126.6, 126.4, 124.1, 73.2, 26.0, 20.6, 20.5. HRMS (ESI), *m*/*z*: [M + Na]^+^ calcd. for C_20_H_18_N_4_NaO_3_ 385.1277; found 385.1251.

**3-Phenyl-6-((3-phenyl-1,2,4-oxadiazol-5-yl)methyl)-4*H*-1,2,4-oxadiazin-5(6*H*)-one 5b**. Beige powder; 61% yield (204 mg); m.p. 194–196 °C. ^1^H NMR (400 MHz, DMSO-*d*_6_): *δ* 11.59 (s, 1H), 8.05–7.99 (m, 2H), 7.79–7.75 (m, 2H), 7.61–7.47 (m, 6H), 4.95 (dd, *J* = 8.0, 4.6 Hz, 1H), 3.78 (dd, *J* = 16.5, 4.6 Hz, 1H), 3.59 (dd, *J* = 16.4, 8.0 Hz, 1H). ^13^C NMR (101 MHz, DMSO-*d*_6_): *δ* 177.2, 168.1, 166.2, 152.5, 132.1, 131.8, 129.7, 129.2, 129.1, 127.5, 127.3, 126.5, 73.1, 26.2. HRMS (ESI), *m*/*z*: [M + Na]^+^ calcd. for C_18_H_14_N_4_NaO_3_ 357.0958; found 357.0943.

**3-(4-Methoxyphenyl)-6-((3-(4-methoxyphenyl)-1,2,4-oxadiazol-5-yl)methyl)-4*H*-1,2,4-oxadiazin-5(6*H*)-one 5c.** Beige powder; 51% yield (178 mg); m.p. 181–182 °C. ^1^H NMR (400 MHz, DMSO-*d*_6_): *δ* 11.49 (s, 1H), 7.95 (d, *J* = 8.8 Hz, 2H), 7.72 (d, *J* = 8.9 Hz, 2H), 7.11 (d, *J* = 8.8 Hz, 2H), 7.04 (d, *J* = 8.9 Hz, 2H), 4.89 (dd, *J* = 8.0, 4.5 Hz, 1H), 3.84 (s, 3H), 3.82 (s, 3H), 3.73 (dd, *J* = 16.4, 4.6 Hz, 1H), 3.53 (dd, *J* = 16.4, 8.1 Hz, 1H). ^13^C NMR (101 MHz, DMSO-*d*_6_): *δ* 176.9, 167.8, 166.4, 162.2, 162.1, 152.3, 129.2, 128.8, 121.3, 118.8, 115.1, 114.5, 73.1, 55.9, 26.2. HRMS (ESI), *m*/*z*: [M + Na]^+^ calcd. for C_20_H_18_N_4_NaO_5_ 417.1169; found 417.1165.

**3-(4-Nitrophenyl)-6-((3-(4-nitrophenyl)-1,2,4-oxadiazol-5-yl)methyl)-4*H*-1,2,4-oxadiazin-5(6*H*)-one 5d.** Yellow powder; 76% yield (322 mg); m.p. 216–217 °C. ^1^H NMR (400 MHz, DMSO-*d*_6_): *δ* 11.87 (s, 1H), 8.41 (d, *J* = 8.7 Hz, 2H), 8.33 (d, *J* = 8.7 Hz, 2H), 8.26 (d, *J* = 8.6 Hz, 2H), 8.03 (d, *J* = 8.9 Hz, 2H), 5.04 (dd, *J* = 7.9, 4.6 Hz, 1H), 3.85 (dd, *J* = 16.6, 4.7 Hz, 1H), 3.68 (dd, *J* = 16.6, 7.9 Hz, 1H). ^13^C NMR (101 MHz, DMSO-*d*_6_): *δ* 178.0, 166.9, 165.8, 151.3, 149.7, 149.5, 135.2, 132.2, 128.9, 128.8, 125.0, 124.2, 73.1, 26.2. HRMS (ESI), *m*/*z*: [M + Na]^+^ calcd. for C_18_H_12_N_6_NaO_7_ 447.0660; found 447.0659.

**3-(4-(Trifluoromethyl)phenyl)-6-((3-(4-(trifluoromethyl)phenyl)-1,2,4-oxadiazol-5-yl)methyl)-4*H*-1,2,4-oxadiazin-5(6*H*)-one 5e.** Light-yellow powder; 53% yield (249 mg); m.p. 203–205 °C. ^1^H NMR (400 MHz, DMSO-*d*_6_): *δ* 11.79 (s, 1H), 8.21 (d, *J* = 8.1 Hz, 2H), 7.99–7.93 (m, 4H), 7.86 (d, *J* = 8.3 Hz, 2H), 5.02 (dd, *J* = 7.8, 4.6 Hz, 1H), 3.82 (dd, *J* = 16.5, 4.7 Hz, 1H), 3.64 (dd, *J* = 16.5, 7.8 Hz, 1H). ^13^C NMR (101 MHz, DMSO-*d*_6_): *δ* 177.8, 167.2, 166.1, 151.8, 133.3, 131.9 (d, *J* = 31.1 Hz), 131.6 (d, *J* = 30.9 Hz), 130.3, 128.3 (d, *J* = 11.7 Hz), 126.8 (q, *J* = 3.6 Hz), 126.0 (q, *J* = 3.6 Hz), 125.7, 122.9, 120.2, 73.1, 26.3.^19^F NMR (376 MHz, DMSO-*d*_6_): *δ* –61.47, –61.56. HRMS (ESI), *m*/*z*: [M + H]^+^ calcd. for C_20_H_13_F_6_N_4_O_3_ 471.0886; found 471.0901.

**3-(4-Fluorophenyl)-6-((3-(4-fluorophenyl)-1,2,4-oxadiazol-5-yl)methyl)-4*H*-1,2,4-oxadiazin-5(6*H*)-one 5f.** Beige powder; 68% yield (252 mg); m.p. 175–177 °C. ^1^H NMR (400 MHz, DMSO-*d*_6_): *δ* 11.62 (s, 1H), 8.06 (dd, *J* = 8.7, 5.6 Hz, 2H), 7.82 (dd, *J* = 8.8, 5.5 Hz, 2H), 7.38 (dt, *J* = 28.2, 8.9 Hz, 4H), 4.94 (dd, *J* = 7.9, 4.6 Hz, 1H), 3.77 (dd, *J* = 16.5, 4.6 Hz, 1H), 3.58 (dd, *J* = 16.5, 8.0 Hz, 1H). ^13^C NMR (101 MHz, DMSO-*d*_6_): *δ* 177.3, 167.3, 166.1, 165.6 (d, *J* = 12.3 Hz), 163.1 (d, *J* = 12.2 Hz), 151.8, 130.0 (d, *J* = 9.1 Hz), 129.9 (d, *J* = 8.9 Hz), 125.7 (d, *J* = 3.1 Hz), 123.1 (d, *J* = 3.1 Hz), 116.9 (d, *J* = 22.2 Hz), 116.2 (d, *J* = 22.2 Hz), 73.0, 26.2. ^19^F NMR (376 MHz, DMSO-*d*_6_): *δ* –108.41, –108.95. HRMS (ESI), *m*/*z*: [M + H]^+^ calcd. for C_18_H_13_F_2_N_4_O_3_ 371.0950; found 371.0951.

**3-(4-Chlorophenyl)-6-((3-(4-chlorophenyl)-1,2,4-oxadiazol-5-yl)methyl)-4*H*-1,2,4-oxadiazin-5(6*H*)-one 5g.** Beige powder; 70% yield (282 mg); m.p. 200–201 °C. ^1^H NMR (400 MHz, DMSO-*d*_6_): *δ* 11.65 (s, 1H), 8.01 (d, *J* = 8.4 Hz, 2H), 7.78 (d, *J* = 8.3 Hz, 2H), 7.65 (d, *J* = 8.5 Hz, 2H), 7.57 (d, *J* = 8.3 Hz, 2H), 4.95 (dd, *J* = 7.9, 4.6 Hz, 1H), 3.77 (dd, *J* = 16.5, 4.6 Hz, 1H), 3.59 (dd, *J* = 16.5, 7.9 Hz, 1H). ^13^C NMR (101 MHz, DMSO-*d*_6_): *δ* 177.5, 167.3, 166.1, 151.8, 136.8, 136.6, 129.9, 129.3, 129.2, 129.1, 128.1, 125.3, 73.0, 26.2. HRMS (ESI), *m*/*z*: [M + H]^+^ calcd. for C_18_H_13_Cl_2_N_4_O_3_ 403.0359; found 403.0378.

**3-(4-Aminophenyl)-6-((3-(4-aminophenyl)-1,2,4-oxadiazol-5-yl)methyl)-4*H*-1,2,4-oxadiazin-5(6*H*)-one 5h.** Beige powder; 57% yield (208 mg); m.p. 180–181 °C. ^1^H NMR (400 MHz, DMSO-*d*_6_): *δ* 11.21 (s, 1H), 7.67 (d, *J* = 8.4 Hz, 2H), 7.44 (d, *J* = 8.4 Hz, 2H), 6.66 (d, *J* = 8.5 Hz, 2H), 6.59 (d, *J* = 8.4 Hz, 2H), 5.75 (s, 2H), 5.70 (s, 2H), 4.78 (dd, *J* = 8.2, 4.5 Hz, 1H), 3.64 (dd, *J* = 16.4, 4.6 Hz, 1H), 3.43 (dd, *J* = 16.3, 8.2 Hz, 1H). ^13^C NMR (101 MHz, DMSO-*d*_6_): *δ* 176.1, 168.3, 166.6, 152.8, 152.3, 152.2, 128.8, 128.4, 115.3, 114.0, 113.5, 113.1, 73.2, 26.1. HRMS (ESI), *m*/*z*: [M + Na]^+^ calcd. for C_18_H_16_N_6_NaO_3_ 387.1176; found 387.1177.

**3-(2-Chlorophenyl)-6-((3-(2-chlorophenyl)-1,2,4-oxadiazol-5-yl)methyl)-4*H*-1,2,4-oxadiazin-5(6*H*)-one 5i.** Beige powder; 49% yield (198 mg); m.p. 156–158 °C. ^1^H NMR (400 MHz, CDCl_3_): *δ* 8.08 (s, 1H), 7.96 (dd, *J* = 7.6, 1.9 Hz, 1H), 7.65 (d, *J* = 7.5 Hz, 1H), 7.56 (d, *J* = 7.7 Hz, 1H), 7.53–7.36 (m, 5H), 5.04 (dd, *J* = 8.2, 4.5 Hz, 1H), 3.83 (dd, *J* = 16.5, 4.5 Hz, 1H), 3.61 (dd, *J* = 16.5, 8.2 Hz, 1H). ^13^C NMR (101 MHz, Acetone-*d*_6_): *δ* 176.0, 167.1, 164.7, 151.3, 132.9, 132.9, 132.3, 132.1, 131.8, 131.5, 130.9, 129.9, 129.1, 127.4, 127.3, 126.1, 73.3, 25.8.. HRMS (ESI), *m*/*z*: [M + Na]^+^ calcd. for C_18_H_12_Cl_2_N_4_NaO_3_ 425.0179; found 425.0185.

**3-(5-Methylthiophen-2-yl)-6-((3-(5-methylthiophen-2-yl)-1,2,4-oxadiazol-5-yl)methyl)-4*H*-1,2,4-oxadiazin-5(6*H*)-one 5j.** Beige powder; 47% yield (176 mg); m.p. 190–191 °C. ^1^H NMR (400 MHz, DMSO-*d*_6_): *δ* 11.68 (s, 1H), 7.60 (d, *J* = 3.8 Hz, 2H), 6.97 (d, *J* = 3.6 Hz, 1H), 6.88 (d, *J* = 3.7 Hz, 1H), 4.91 (dd, *J* = 8.2, 4.5 Hz, 1H), 3.71 (dd, *J* = 16.5, 4.5 Hz, 1H), 3.52 (dd, *J* = 16.5, 8.1 Hz, 1H), 2.53 (s, 3H), 2.46 (s, 3H). ^13^C NMR (101 MHz, DMSO-*d*_6_): *δ* 176.9, 166.0, 164.2, 148.6, 145.1, 144.7, 130.7, 129.9, 128.3, 127.5, 126.6, 125.2, 73.3, 26.1, 15.5, 15.5. HRMS (ESI), *m*/*z*: [M + Na]^+^ calcd. for C_16_H_14_N_4_NaO_3_S_2_ 397.0400; found 397.0398.

**3-(Pyridin-4-yl)-6-((3-(pyridin-4-yl)-1,2,4-oxadiazol-5-yl)methyl)-4*H*-1,2,4-oxadiazin-5(6*H*)-one 5k.** Beige powder; 35% yield (118 mg); m.p. 208–210 °C. ^1^H NMR (400 MHz, DMSO-*d*_6_): *δ* 11.81 (s, 1H), 8.84–8.79 (m, 2H), 8.75–8.70 (m, 2H), 7.96–7.90 (m, 2H), 7.76–7.72 (m, 2H), 5.03 (dd, *J* = 7.9, 4.6 Hz, 1H), 3.84 (dd, *J* = 16.5, 4.6 Hz, 1H), 3.67 (dd, *J* = 16.5, 7.9 Hz, 1H). ^13^C NMR (101 MHz, DMSO-*d*_6_): *δ* 178.0, 166.9, 165.8, 151.4, 150.9, 150.7, 136.7, 133.7, 121.4, 121.1, 73.1, 26.2. HRMS (ESI), *m*/*z*: [M + H]^+^ calcd. for C_16_H_13_N_6_O_3_ 337.1044; found 337.1039.

**3-(Pyridin-2-yl)-6-((3-(pyridin-2-yl)-1,2,4-oxadiazol-5-yl)methyl)-4*H*-1,2,4-oxadiazin-5(6*H*)-one 5l.** Beige powder; 32% yield (108 mg); m.p. 180–184 °C. ^1^H NMR (400 MHz, CDCl_3_): *δ* 9.48 (s, 1H), 8.78 (d, *J* = 4.8 Hz, 1H), 8.59 (d, *J* = 4.8 Hz, 1H), 8.09 (dd, *J* = 17.7, 7.9 Hz, 2H), 7.82 (q, *J* = 6.9 Hz, 2H), 7.47–7.36 (m, 2H), 5.02 (dd, *J* = 8.5, 4.3 Hz, 1H), 3.84 (dd, *J* = 16.4, 4.3 Hz, 1H), 3.56 (dd, *J* = 16.4, 8.5 Hz, 1H). ^13^C NMR (101 MHz, CDCl_3_): *δ* 176.4, 168.5, 164.0, 150.6, 148.9, 148.7, 146.4, 145.2, 137.7, 137.1, 126.4, 125.7, 123.5, 121.1, 73.9, 26.8. HRMS (ESI), *m*/*z*: [M + H]^+^ calcd. for C_16_H_13_N_6_O_3_ 337.1044; found 337.1065.

## 4. Concluding Remarks

In this work, we found a previously unknown formation of the 1,2,4-oxadiazine core. We have shown that the reaction of aryl or hetaryl amidoximes with maleates or fumarates affords substituted 1,2,4-oxadiazin-5-ones. Using the example of *N*’-hydroxy-4-methylbenzimidamide **1a**, the conditions for this reaction were optimized. We have found that, depending on the base used and the ratio of reactants, different products can be synthesized selectively in good yields. The use of NaOH as the base afforded 2-(5-oxo-3-(*p*-tolyl)-5,6-dihydro-4*H*-1,2,4-oxadiazin-6-yl)acetic acid **3a**, and *t*-BuONa–the corresponding methyl ester **4a**. In case of an excess of **1a**, hybrid 3-(*p*-tolyl)-6-((3-(*p*-tolyl)-1,2,4-oxadiazol-5-yl)methyl)-4*H*-1,2,4-oxadiazin-5(6*H*)-one was the main reaction product. The reaction is tolerant to the electronic effect of substituents in amidoximes. The introduction of the substituent in the *ortho* position of arylamidoxime slightly reduces the yield but does not prevent the reaction in general. As a result, we have developed a simple and convenient method for the synthesis of 3-aryl- and 3-hetaryl-1,2,4-oxadiazin-5-ones bearing an easily functionalizable (methoxycarbonyl)methyl group at position 6.

## Figures and Tables

**Table 1:** Optimization of the reaction conditions.

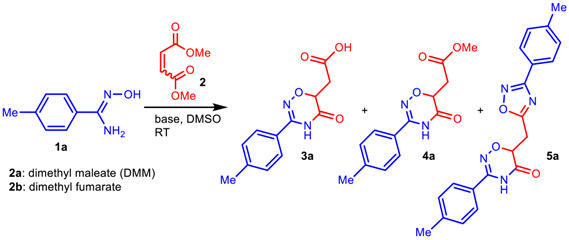						
Entry	2a (Equiv)	Base (Equiv)	Time, h	Isolated Yield, %		
				3a	4a	5a
1	1.5	NaOH (1.5)	4	37	26	–
2	2.0	NaOH (2.0)	4	47	17	–
3	2.5	NaOH (2.5)	4	35	14	–
**4**	**2.0**	**NaOH (2.0)**	**18**	**65**	**trace**	–
5	2.0	NaOH (2.0)	24	61	trace	–
6	2.0	LiOH (2.0)	18	60	trace	–
7	2.0	KOH (2.0)	18	55	trace	–
**8**	**2.0**	***t*-BuONa (1.0)**	**4**	–	**78**	**trace**
9	2.0	*t*-BuONa (2.0)	4	–	58	18
10	0.4	*t*-BuONa (2.0)	4	–	29	54
**11**	**0.4**	***t*-BuONa (2.0)**	**18**	–	**trace**	**82**
12 ^a^	2.0	NaOH (2.0)	18	63	trace	–
13 ^a^	2.0	*t*-BuONa (1.0)	4	–	76	trace

^a^ Dimethyl fumarate **2b** was used instead of **2a**.

## Data Availability

The data presented in this study are available on request from the corresponding author.

## Supplementary Materials

The following supporting information can be downloaded at: https://www.mdpi.com/article/10.3390/molecules27217508/s1. Copies of ^1^H, ^13^C, and ^19^F NMR spectra for compounds **3**, **4**, and **5**; X-ray diffraction data.

## Sample Availability

Samples of the compounds are not available from the authors.
