# Supplementary material for: Unusual Formation of 1,2,4-Oxadiazine Core in Reaction of Amidoximes with Maleic or Fumaric Esters
Source: Molecules. 2022 Nov 3;27(21):7508. doi: 10.3390/molecules27217508 (PMC9655267; doi:10.3390/molecules27217508)
Supplement: Supplementary file 1 [file molecules-27-07508-s001.zip › molecules-1988170-supplementary.pdf]

Supporting information

for

Sofia I. Presnukhina[1], Marina V. Tarasenko[2], Kirill K. Geyl[1], Svetlana O. Baykova[1], Sergey V. Baykov[1], Anton A. Shetnev[2], and Vadim P. Boyarskiy[1,]*

[1]*Institute of Chemistry, Saint Petersburg State University, Universitetskaya Nab., 7/9, 199034 Saint Petersburg, Russian Federation*

[2] *Pharmaceutical Technology Transfer Centre, Yaroslavl State Pedagogical University Named after K.D. Ushinsky, Respublikanskaya St., 108, 150000 Yaroslavl,* Russian Federation

*Corresponding authors: e–mail: v.boyarskii@spbu.ru.

Content

1. Copies of ^{1}H, ^{13}C, and ^{19}F NMR spectra for compounds **3**, **4**, and **5** 3

2. X–ray diffraction data 51

1. Copies of ^{1}H, ^{13}C, and ^{19}F NMR spectra for compounds 3, 4, and 5

^{1}H and ^{13}C spectra of 2-(5-oxo-3-(4-methylphenyl)-5,6-dihydro-4*H*-1,2,4-oxadiazin-6-yl)acetic acid **3a**

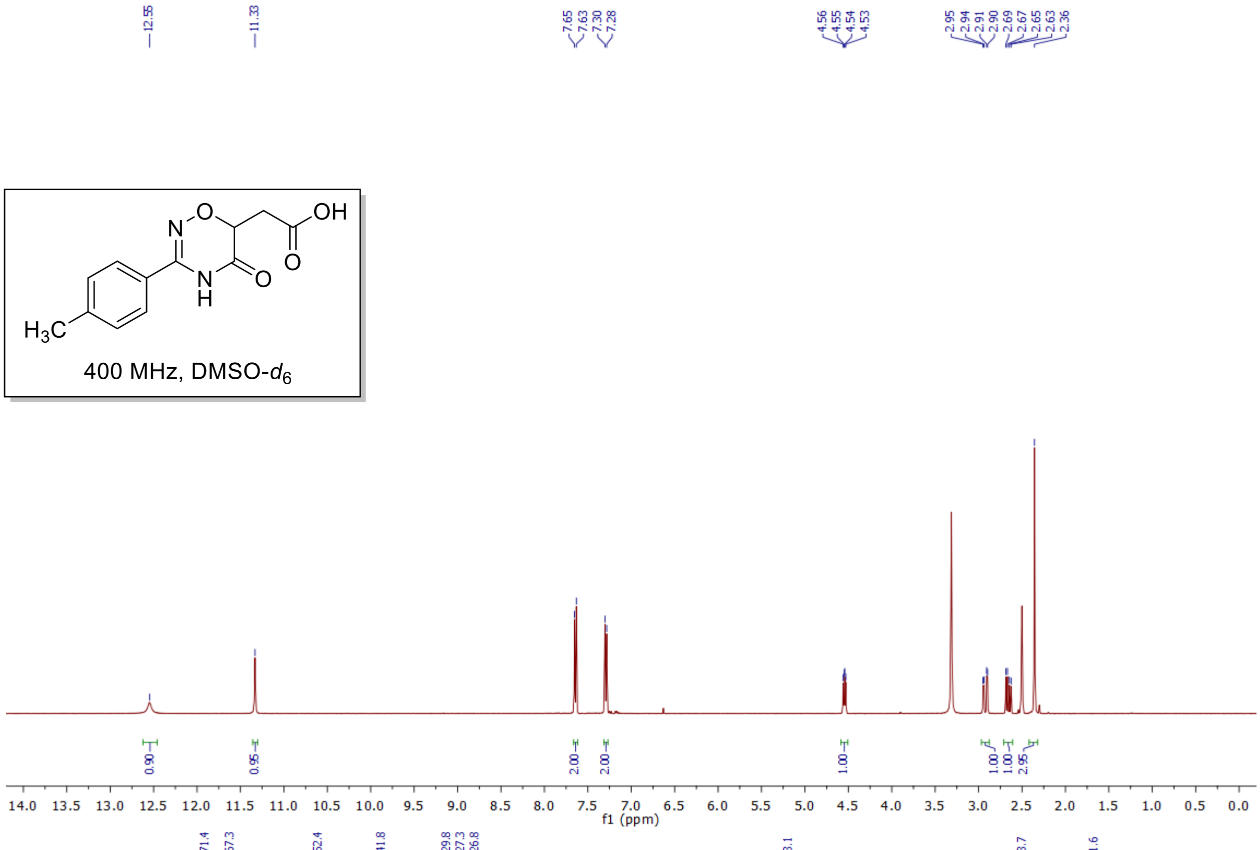

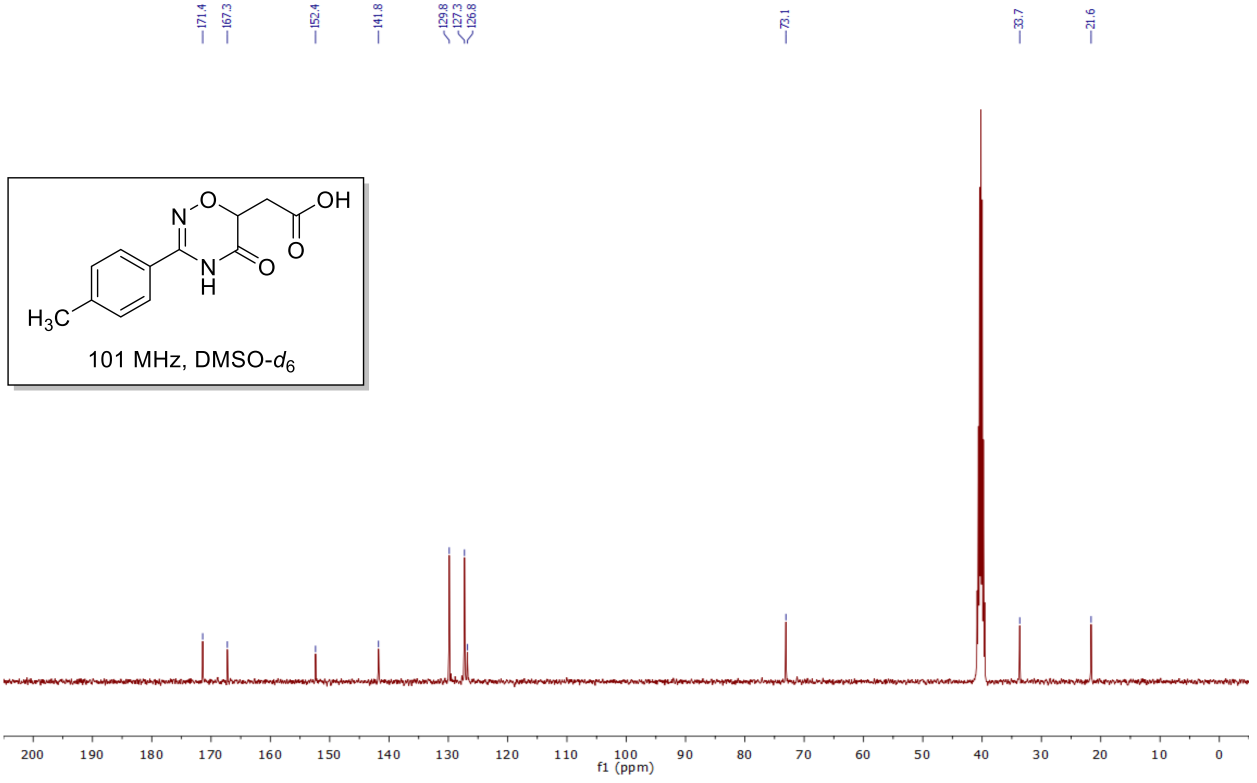

^{1}H and ^{13}C spectra of 2-(5-oxo-3-phenyl-5,6-dihydro-4*H*-1,2,4-oxadiazin-6-yl)acetic acid **3b**

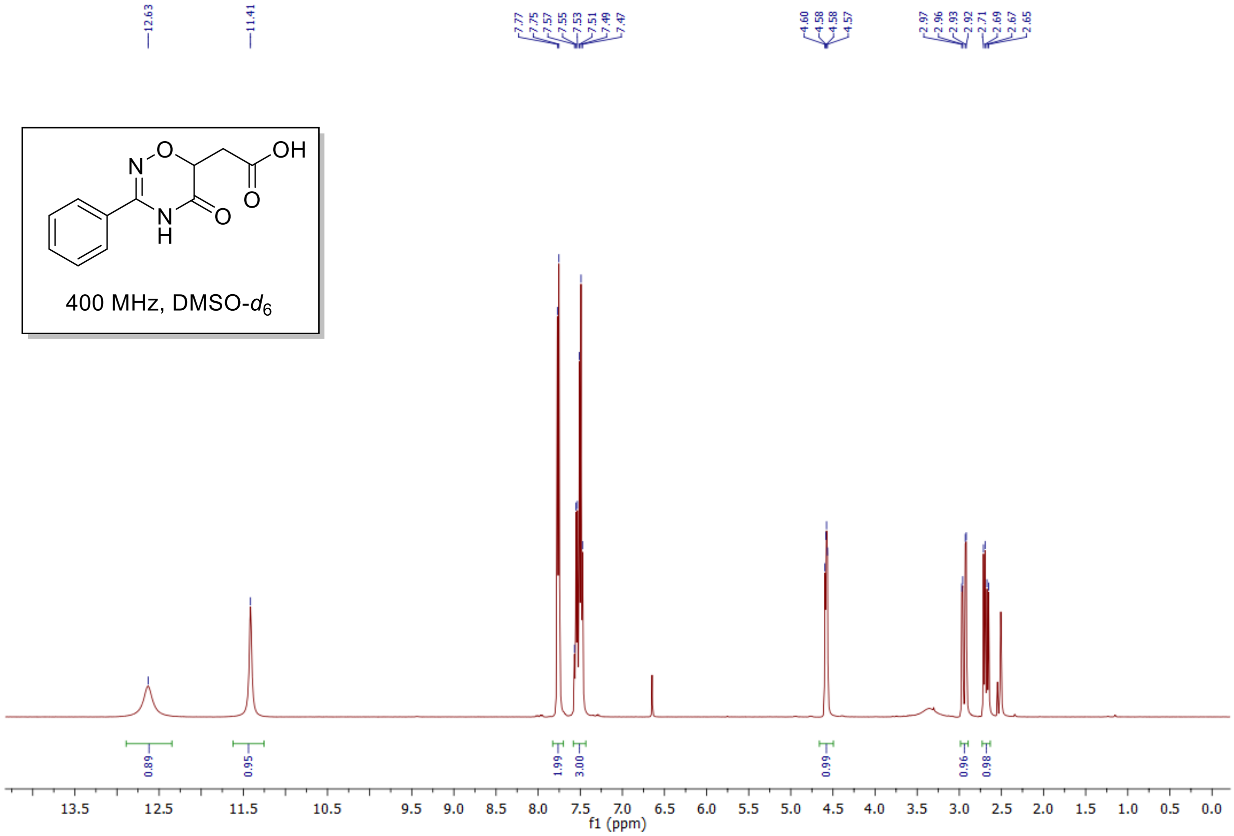

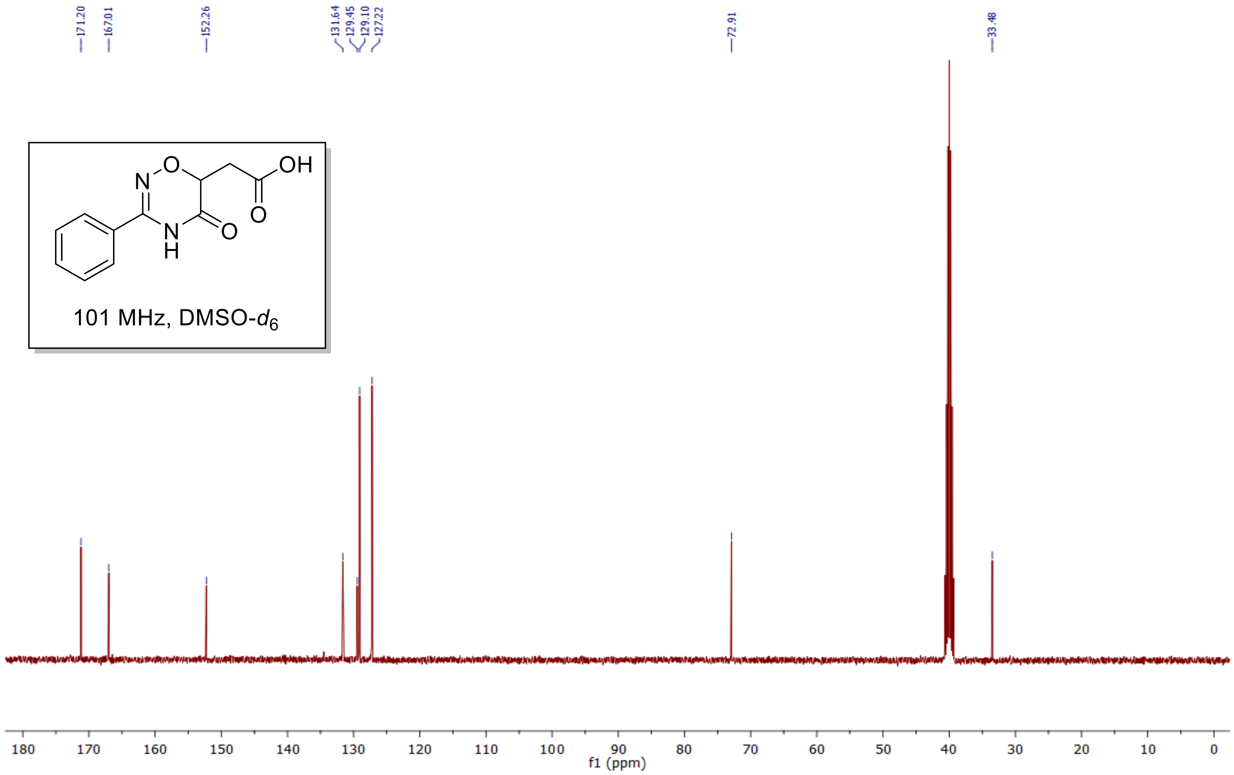

^{1}H and ^{13}C spectra of 2-(3-(4-methoxyphenyl)-5-oxo-5,6-dihydro-4*H*-1,2,4-oxadiazin-6-yl)acetic acid **3c**

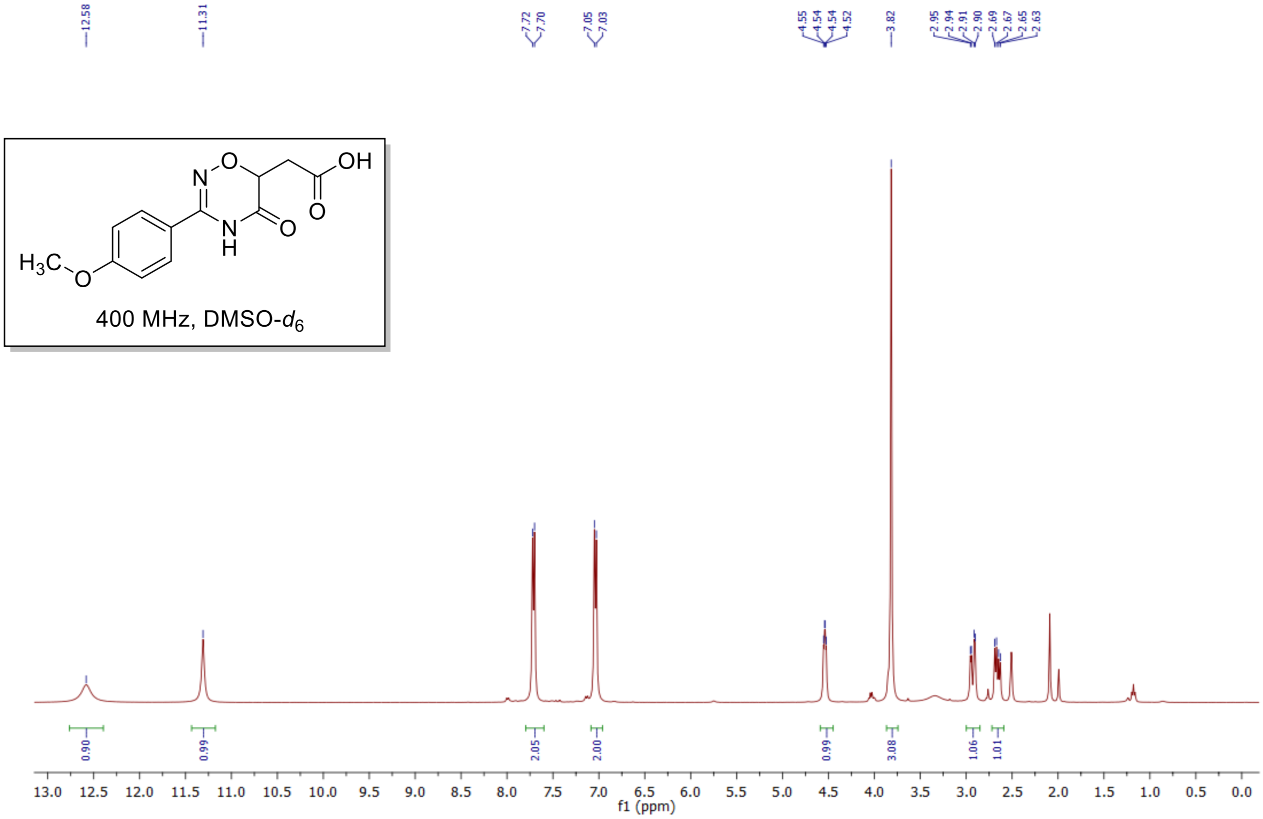

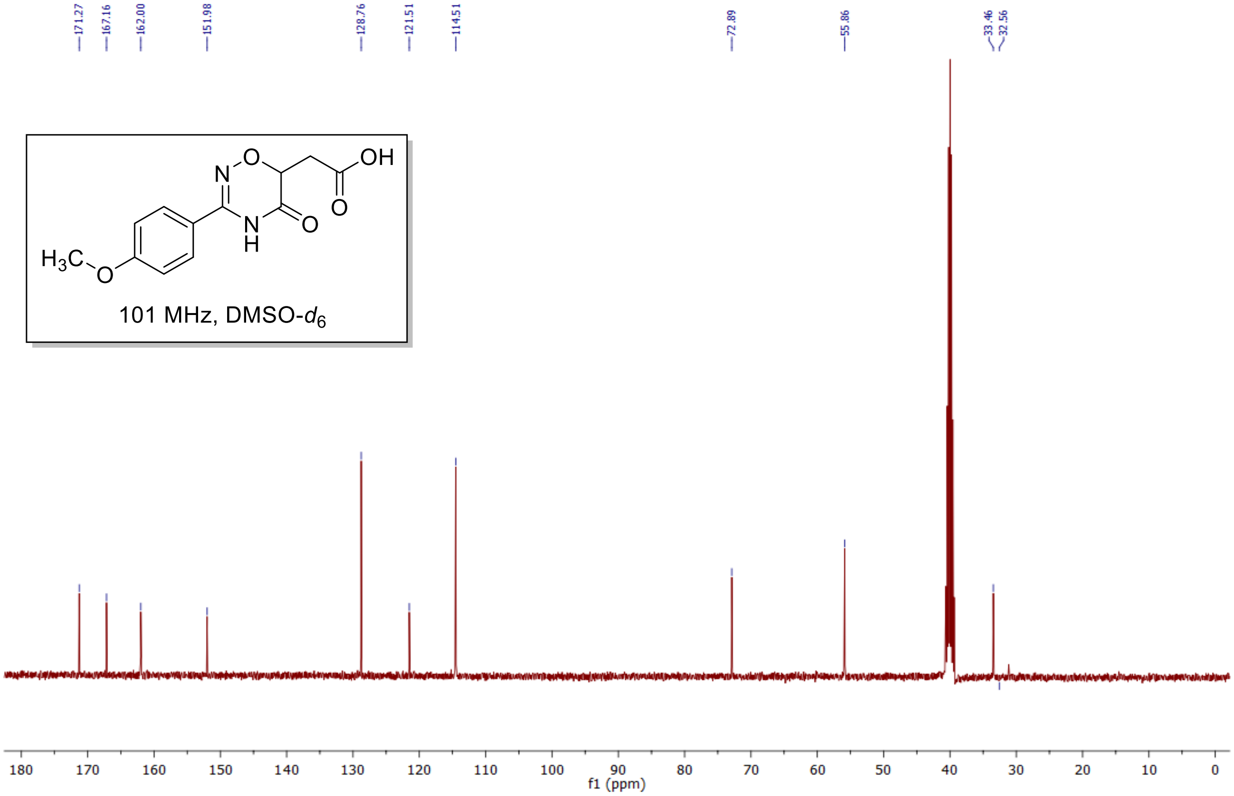

^{1}H and ^{13}C spectra of 2-(3-(4-nitrophenyl)-5-oxo-5,6-dihydro-4*H*-1,2,4-oxadiazin-6-yl)acetic acid **3d**

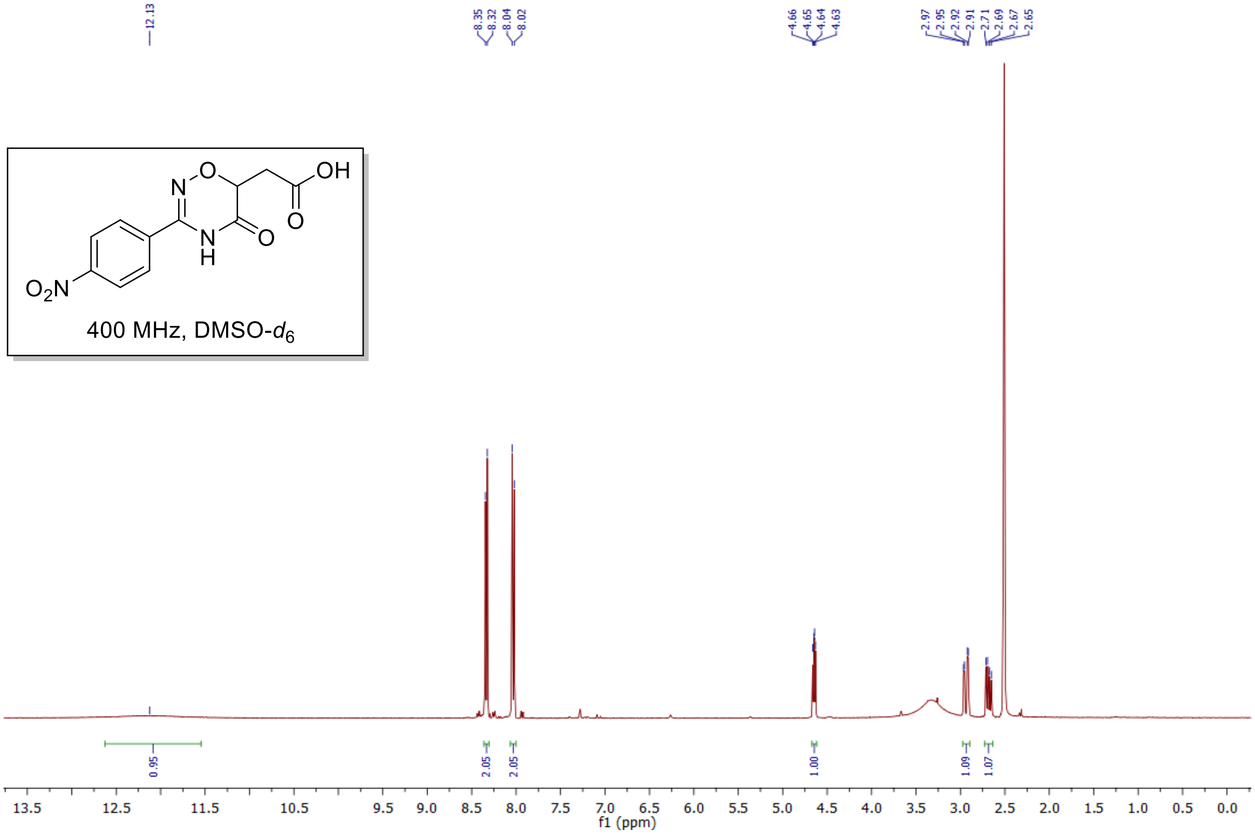

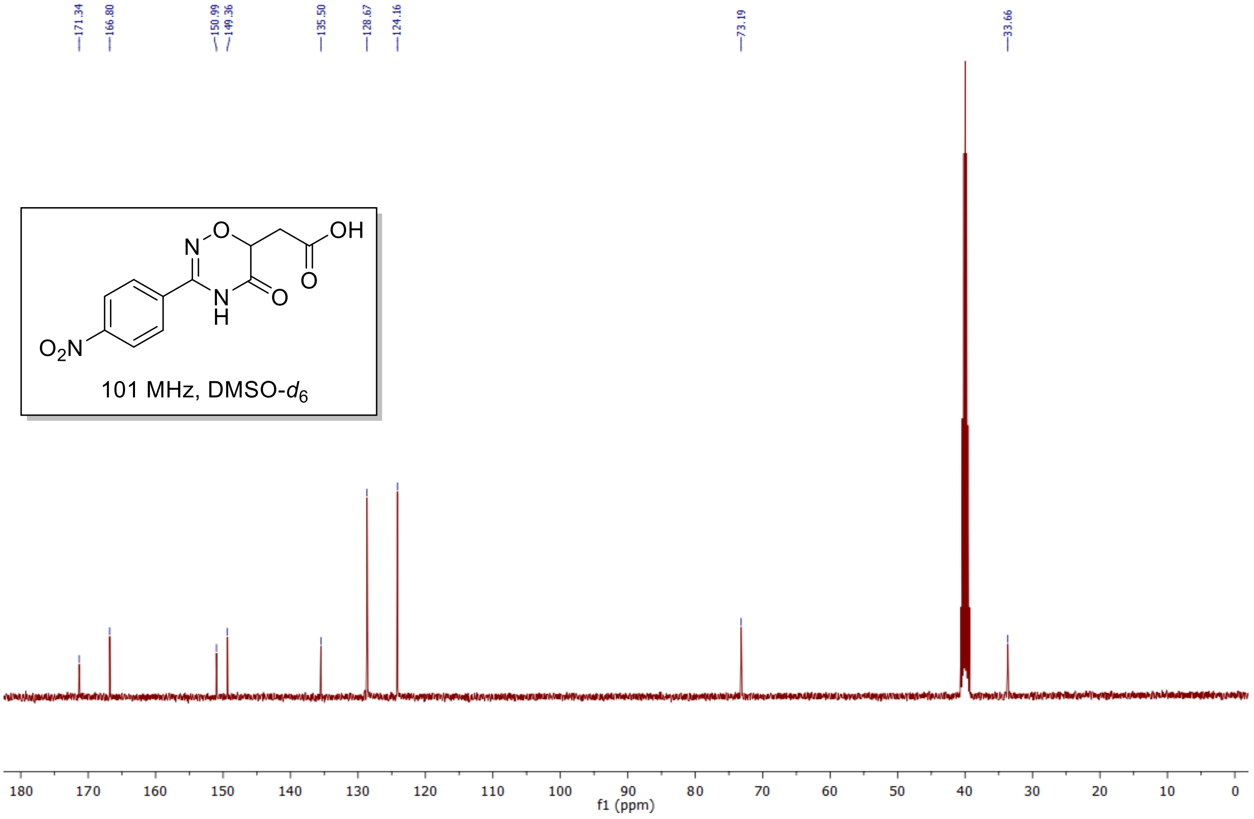

^{1}H, ^{13}C and ^{19}F spectra of 2-(5-oxo-3-(4-(trifluoromethyl)phenyl)-5,6-dihydro-4*H*-1,2,4-oxadiazin-6-yl)acetic acid **3e**

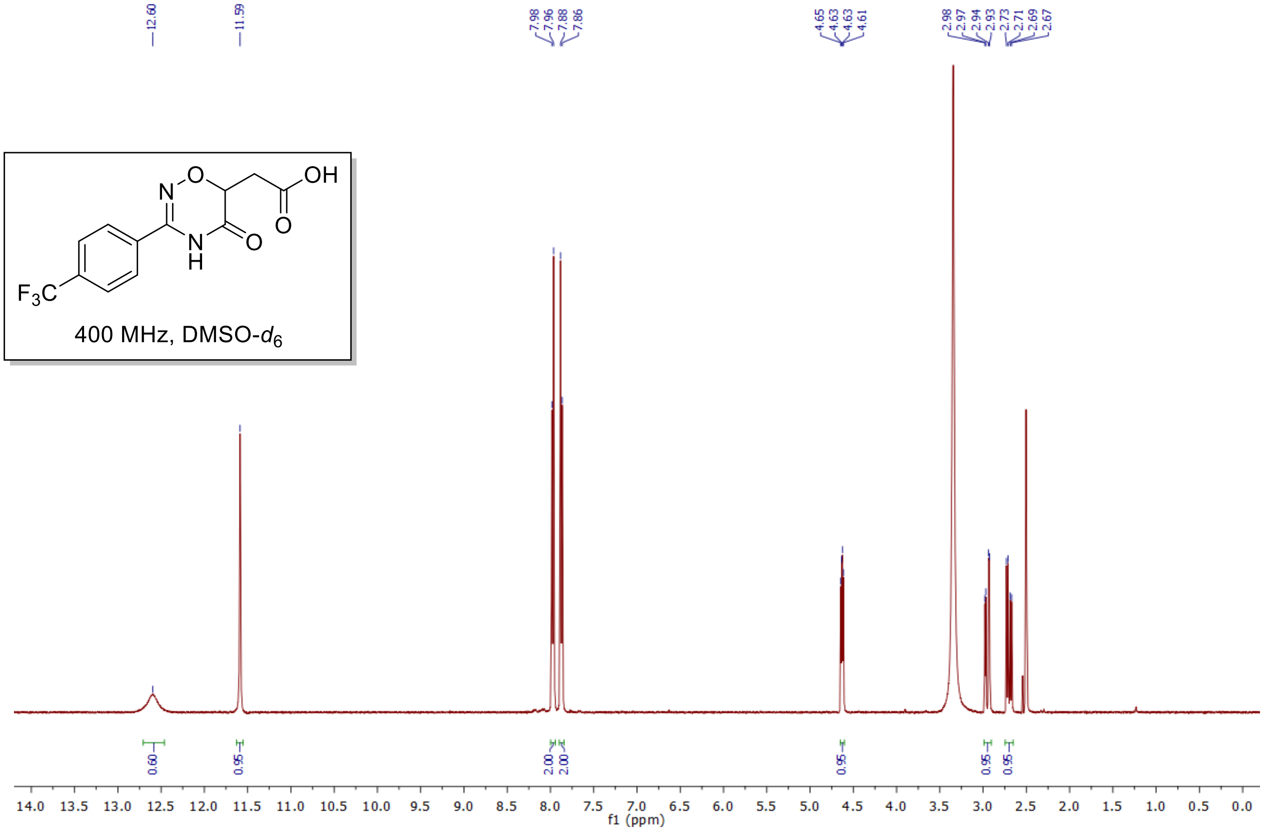

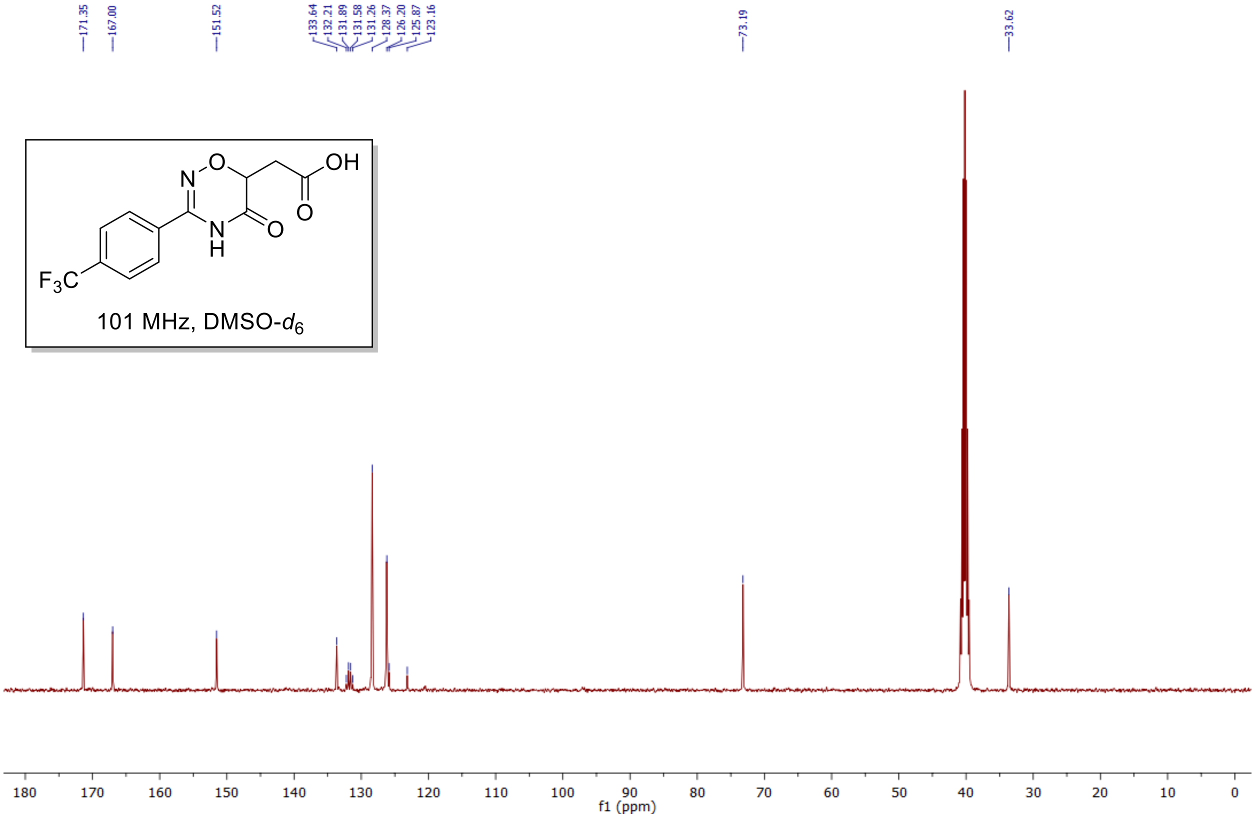

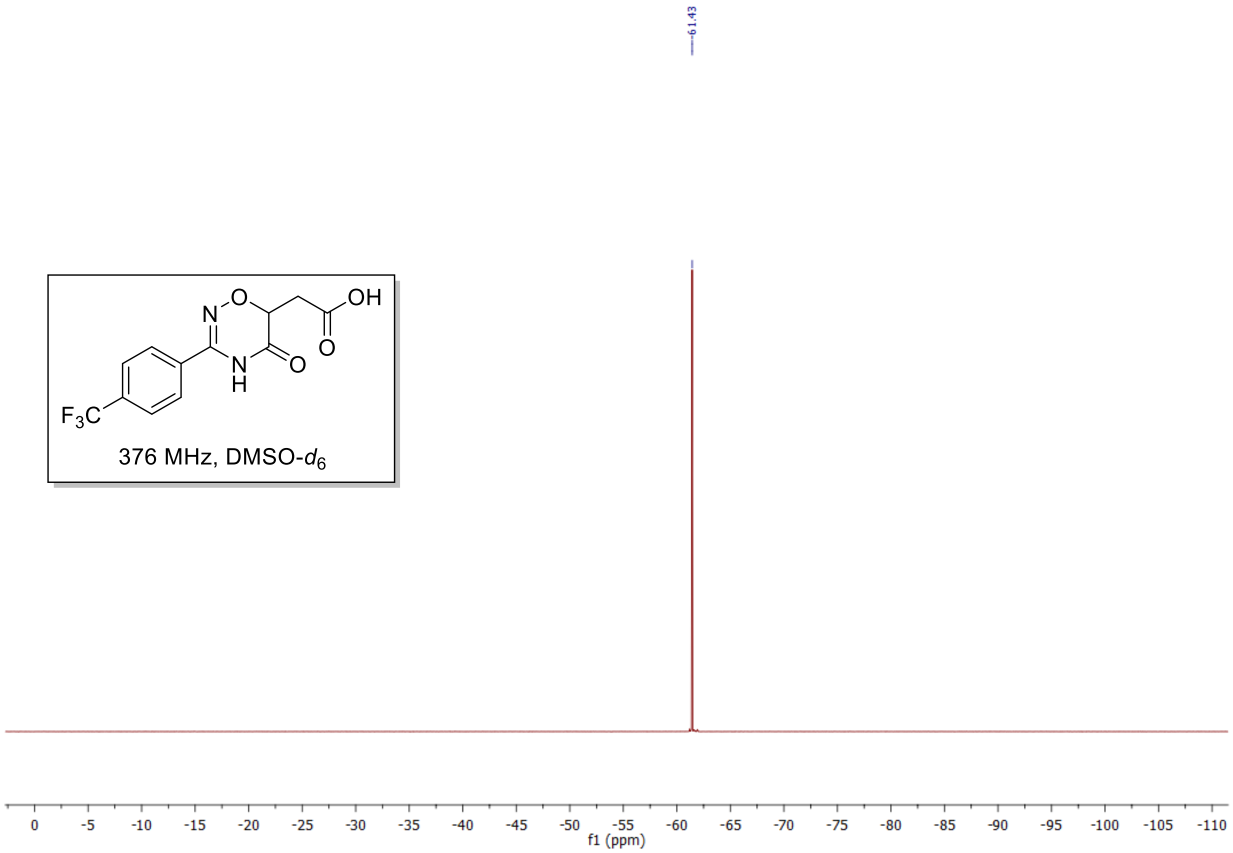
-61.43
376 MHz, DMSO-d6
0 -5 -10 -15 -20 -25 -30 -35 -40 -45 -50 -55 -60 -65 -70 -75 -80 -85 -90 -95 -100 -105 -110
f1 (ppm)

^{1}H, ^{13}C and ^{19}F spectra of 2-(3-(4-fluorophenyl)-5-oxo-5,6-dihydro-4*H*-1,2,4-oxadiazin-6-yl)acetic acid **3f**

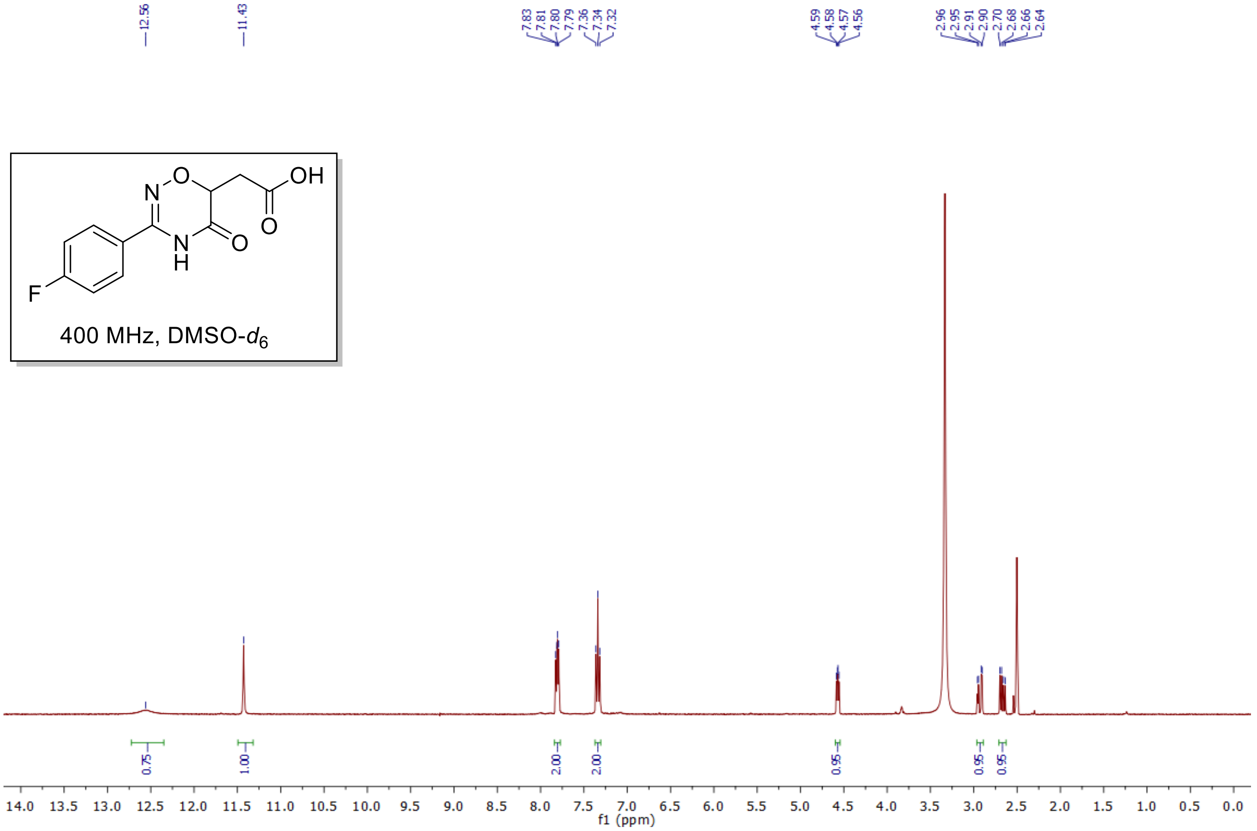

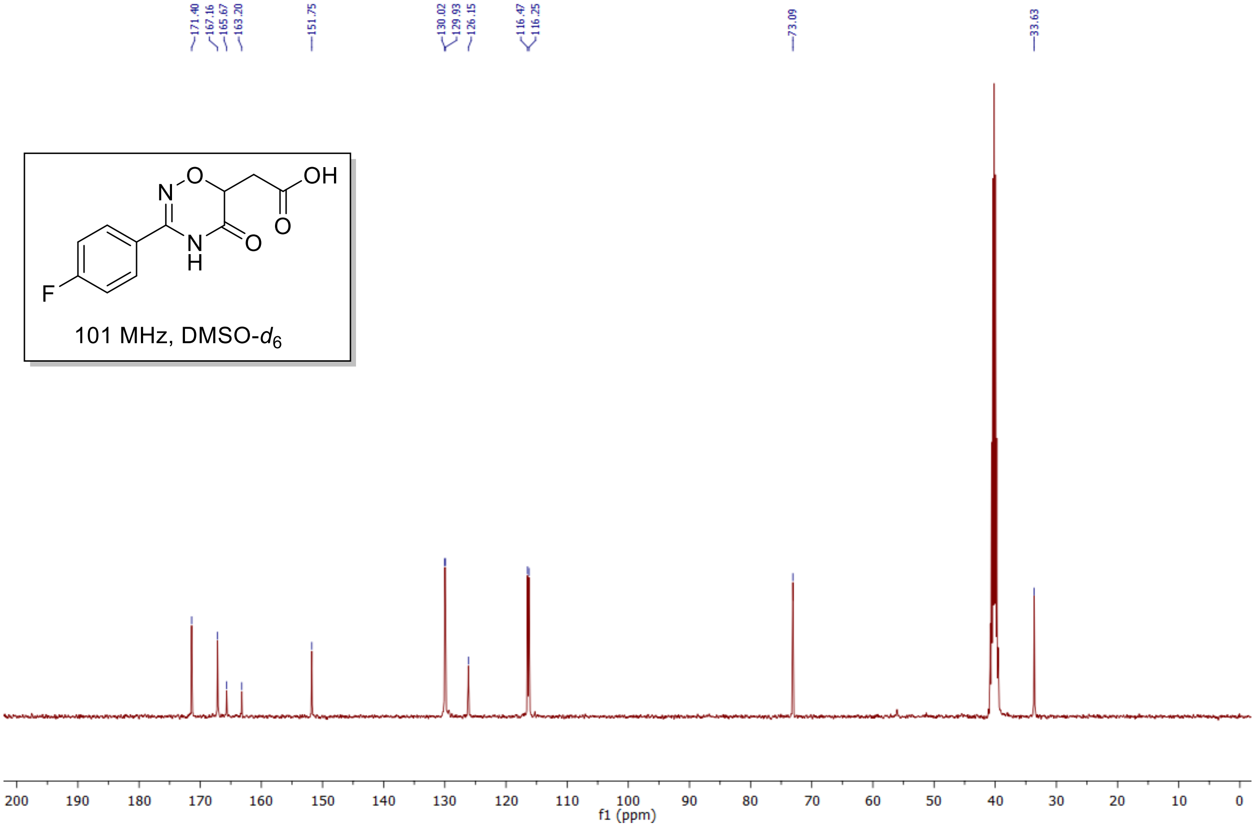

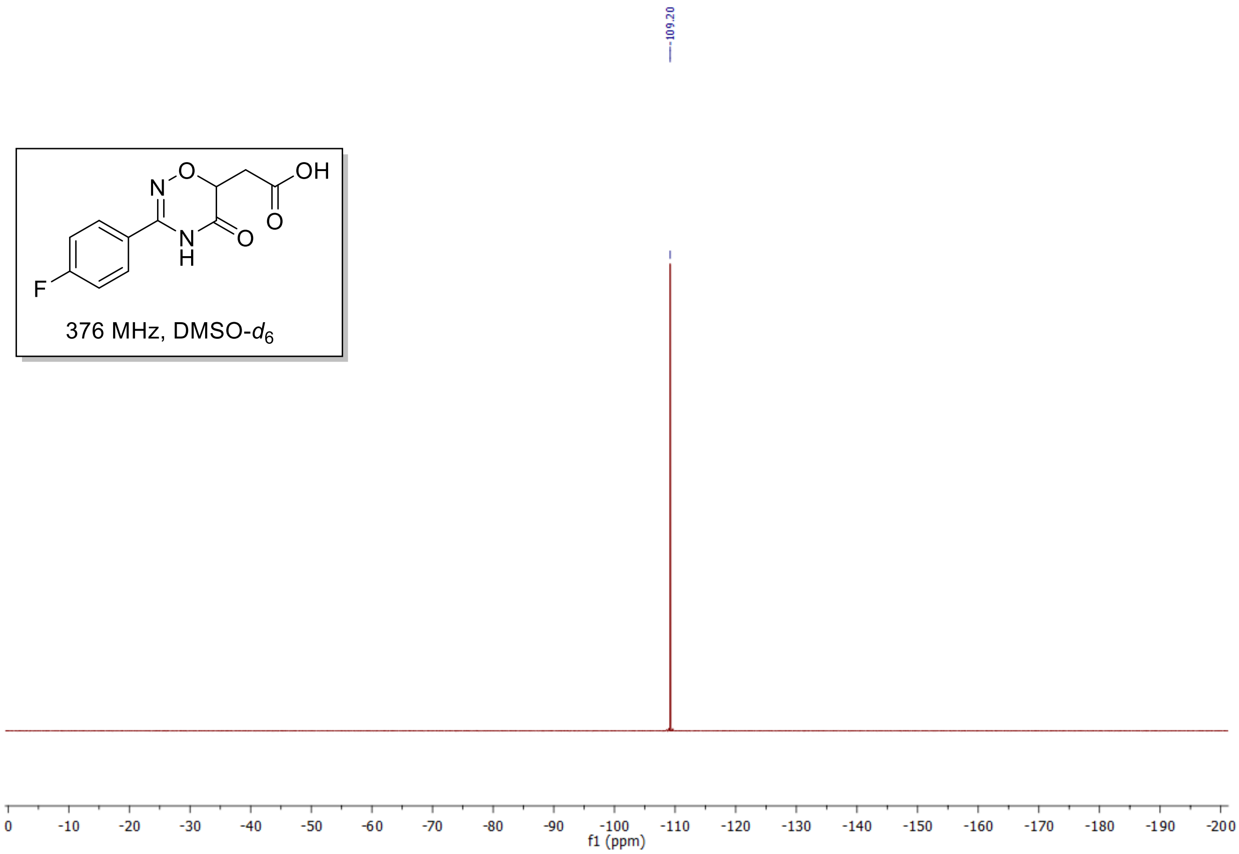
-109.20
376 MHz, DMSO-d6
0
-10
-20
-30
-40
-50
-60
-70
-80
-90
-100
-110
-120
-130
-140
-150
-160
-170
-180
-190
-200
f1 (ppm)

^{1}H and ^{13}C spectra of 2-(3-(4-bromophenyl)-5-oxo-5,6-dihydro-4*H*-1,2,4-oxadiazin-6-yl)acetic acid **3g**

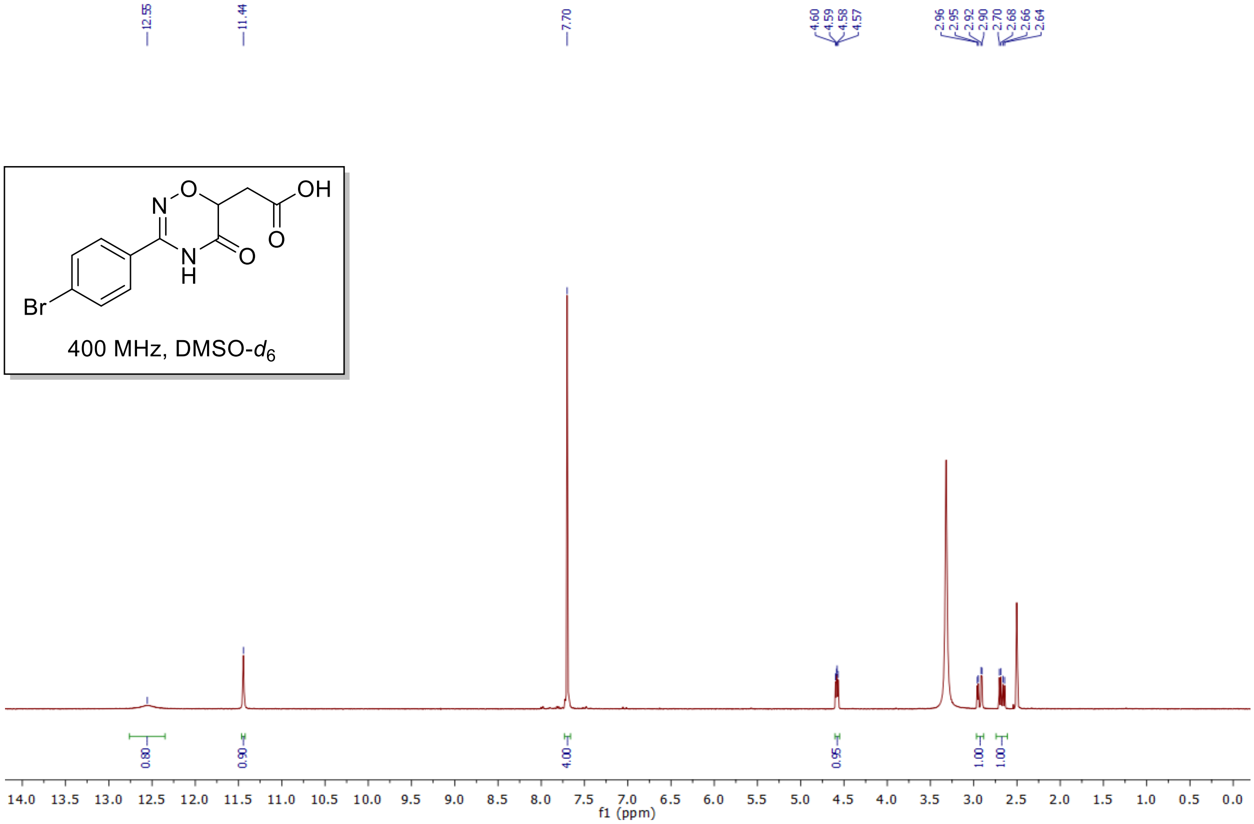

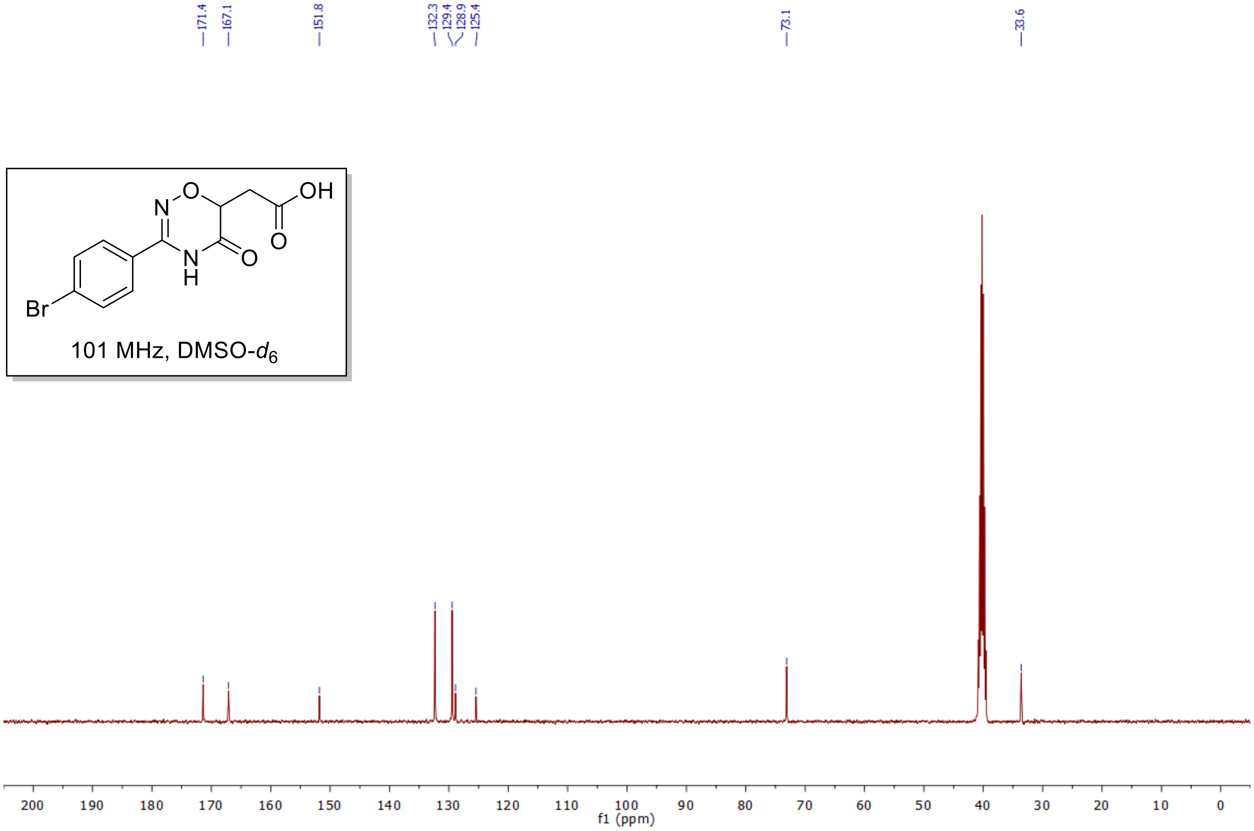

^{1}H and ^{13}C spectra of 2-(3-(4-chlorophenyl)-5-oxo-5,6-dihydro-4*H*-1,2,4-oxadiazin-6-yl)acetic acid **3h**

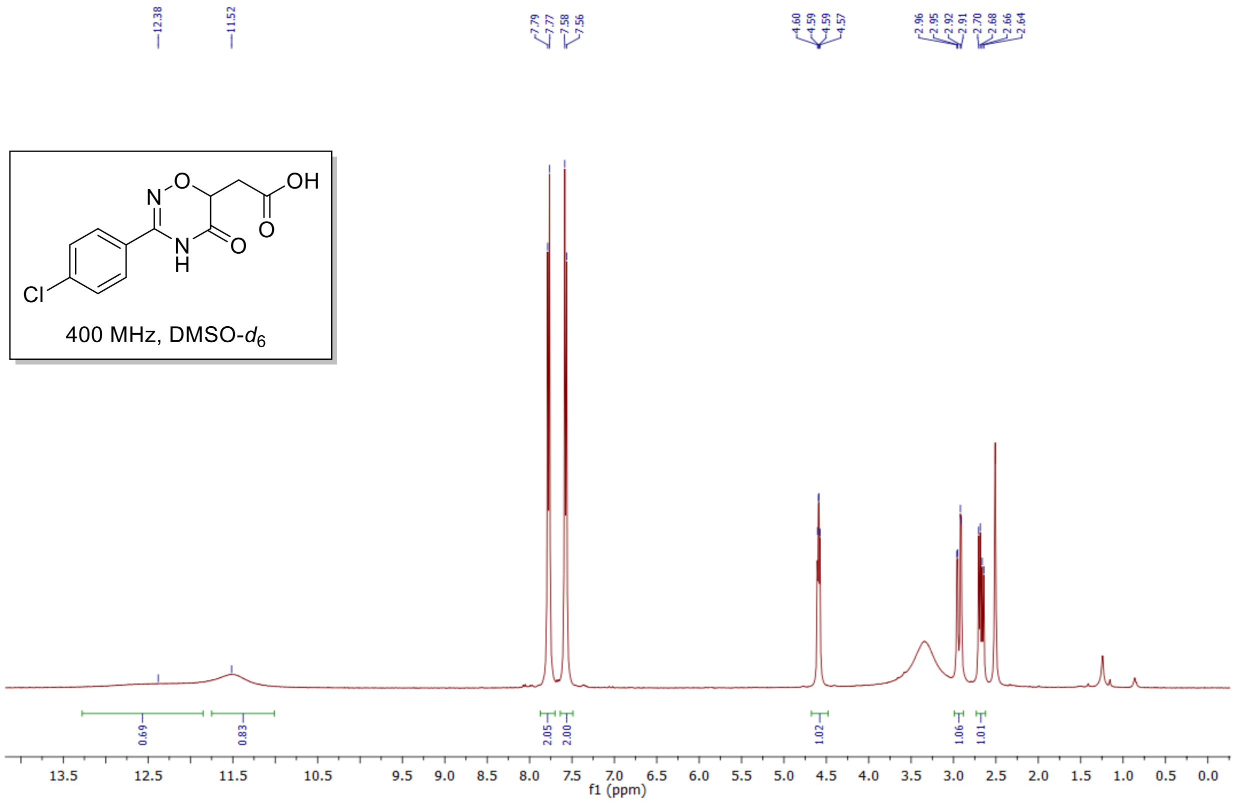

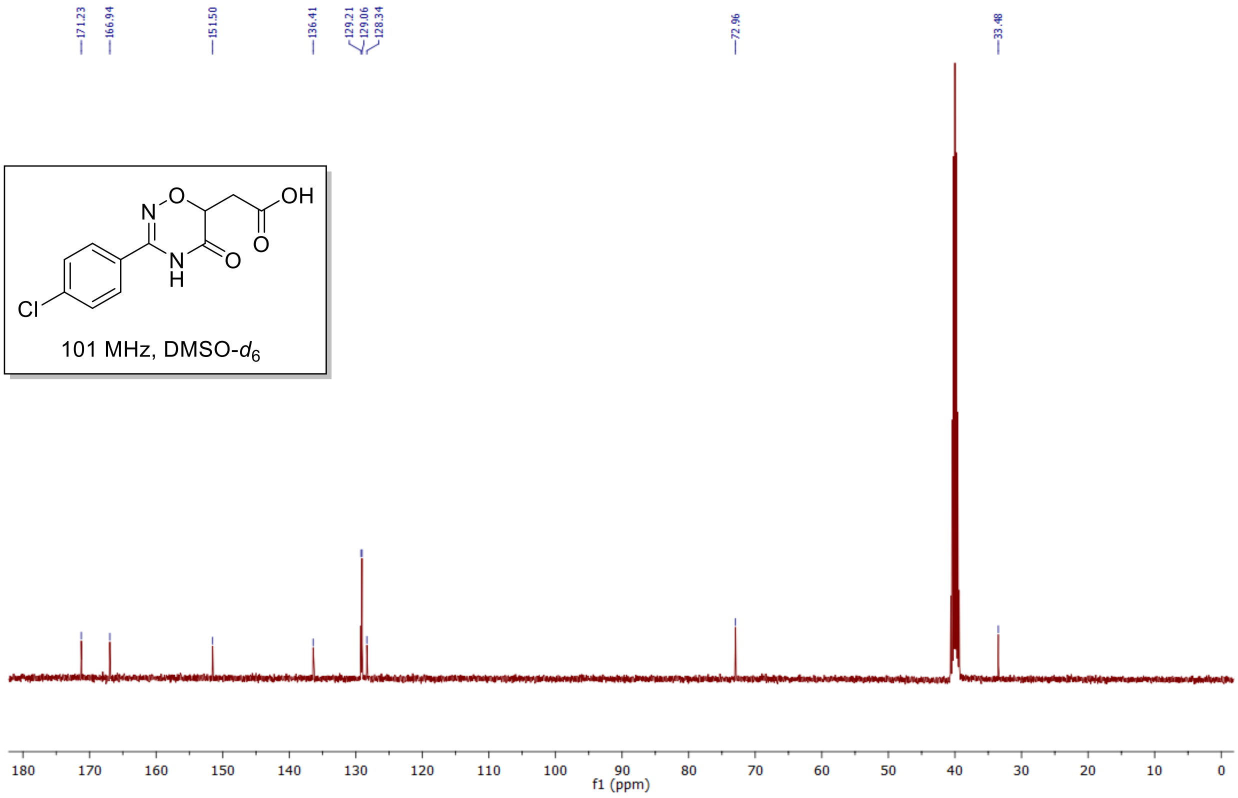

^{1}H and ^{13}C spectra of 2-(5-oxo-3-(4-phenoxyphenyl)-5,6-dihydro-4*H*-1,2,4-oxadiazin-6-yl)acetic acid **3i**

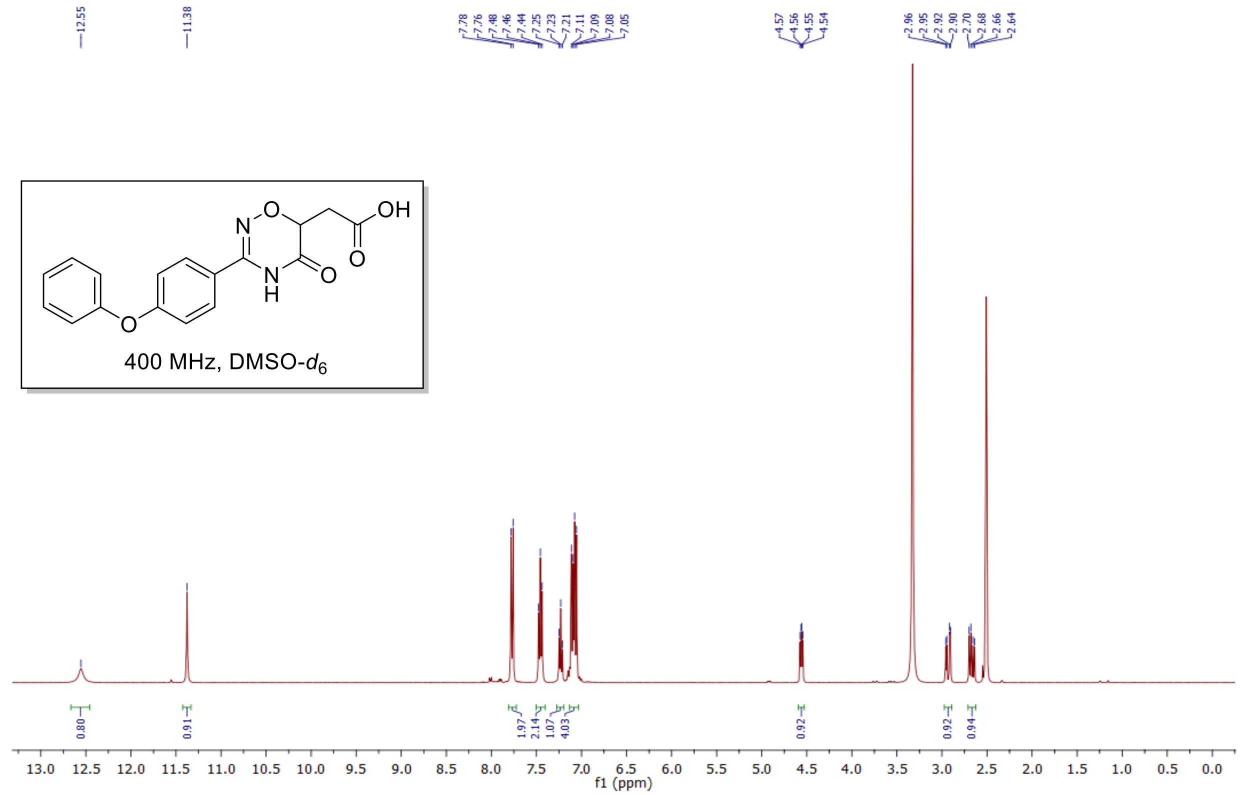

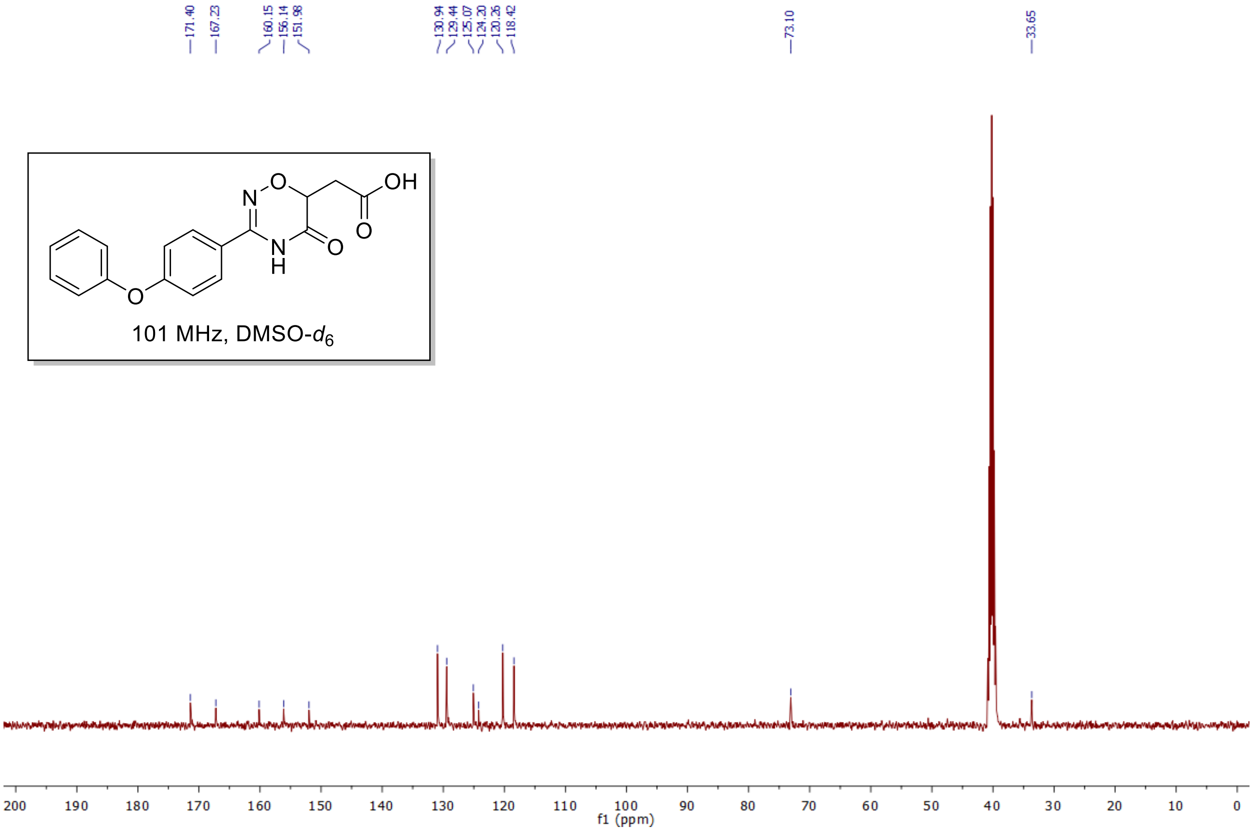

^{1}H and ^{13}C spectra of 2-(5-oxo-3-(2-methylphenyl)-5,6-dihydro-4*H*-1,2,4-oxadiazin-6-yl)acetic acid **3j**

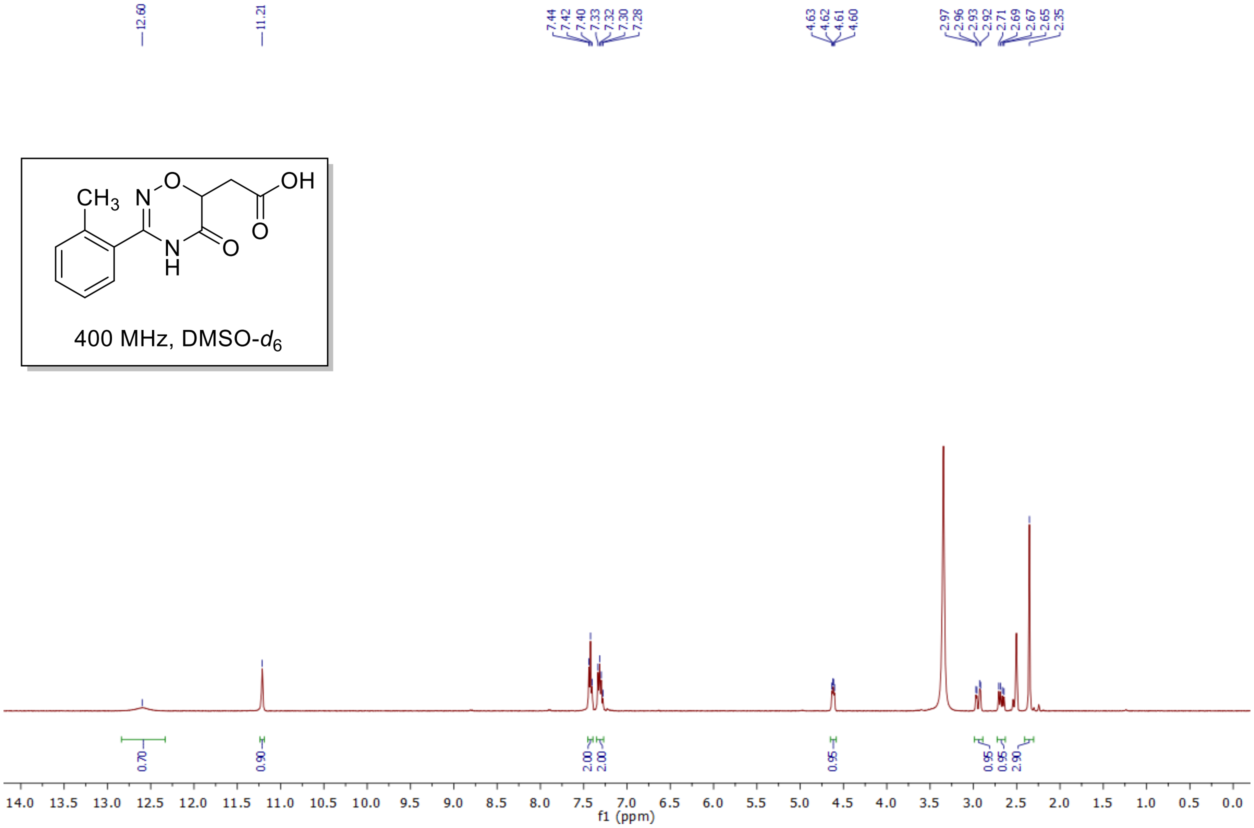

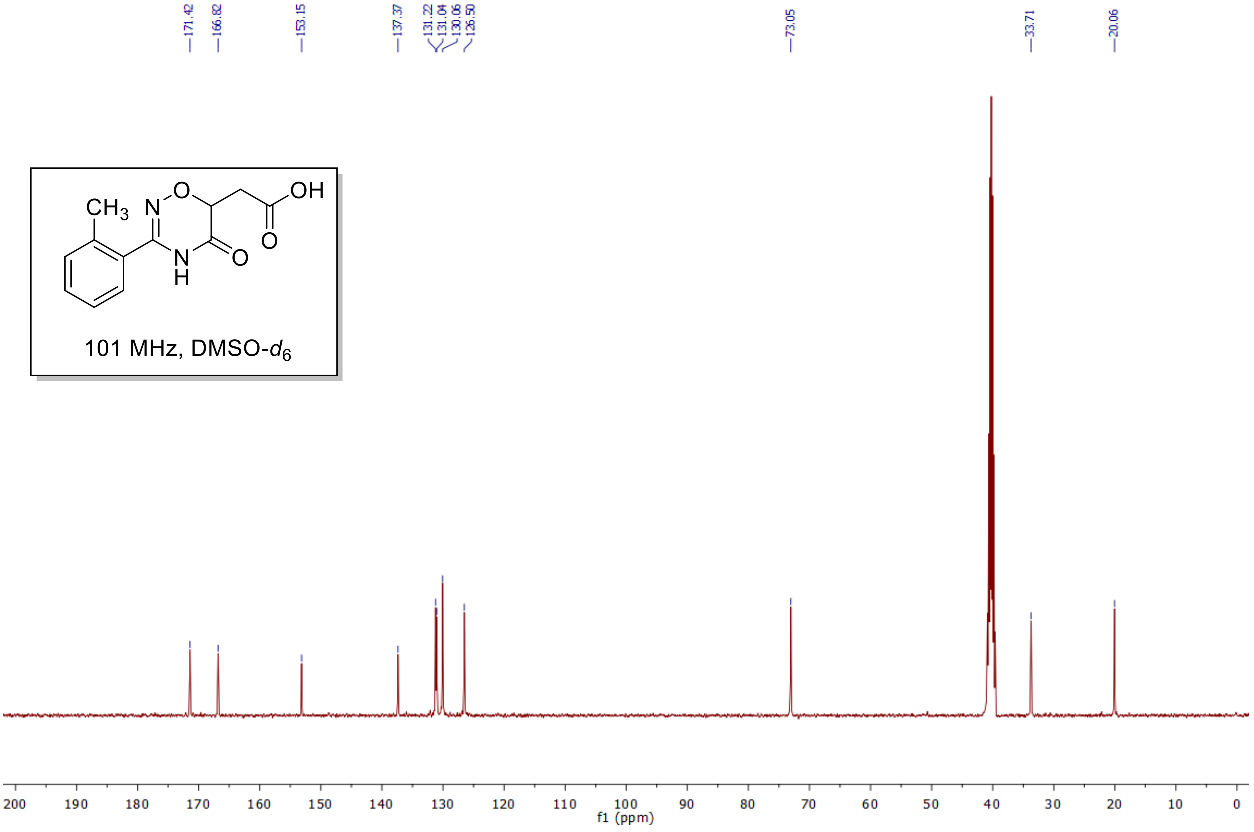

^{1}H and ^{13}C spectra of 2-(3-(2-chlorophenyl)-5-oxo-5,6-dihydro-4*H*-1,2,4-oxadiazin-6-yl)acetic acid **3k**

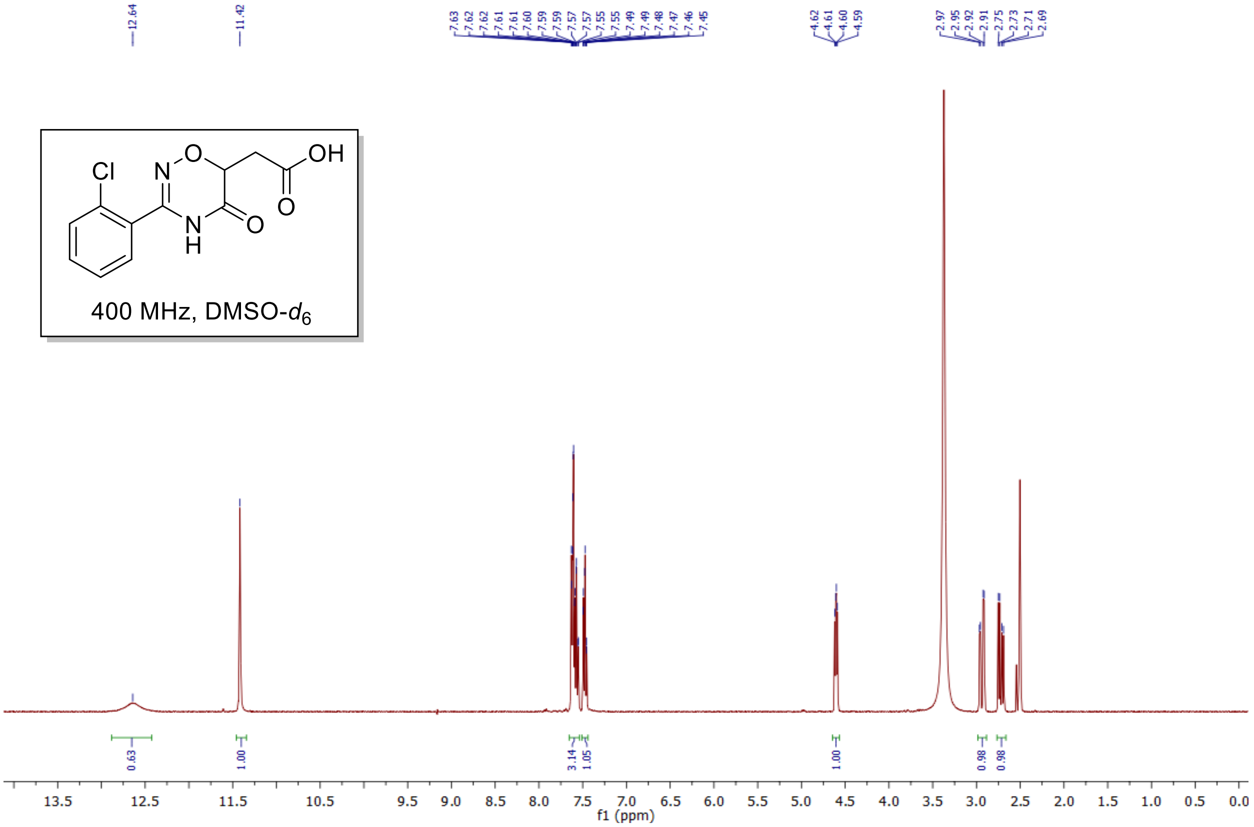

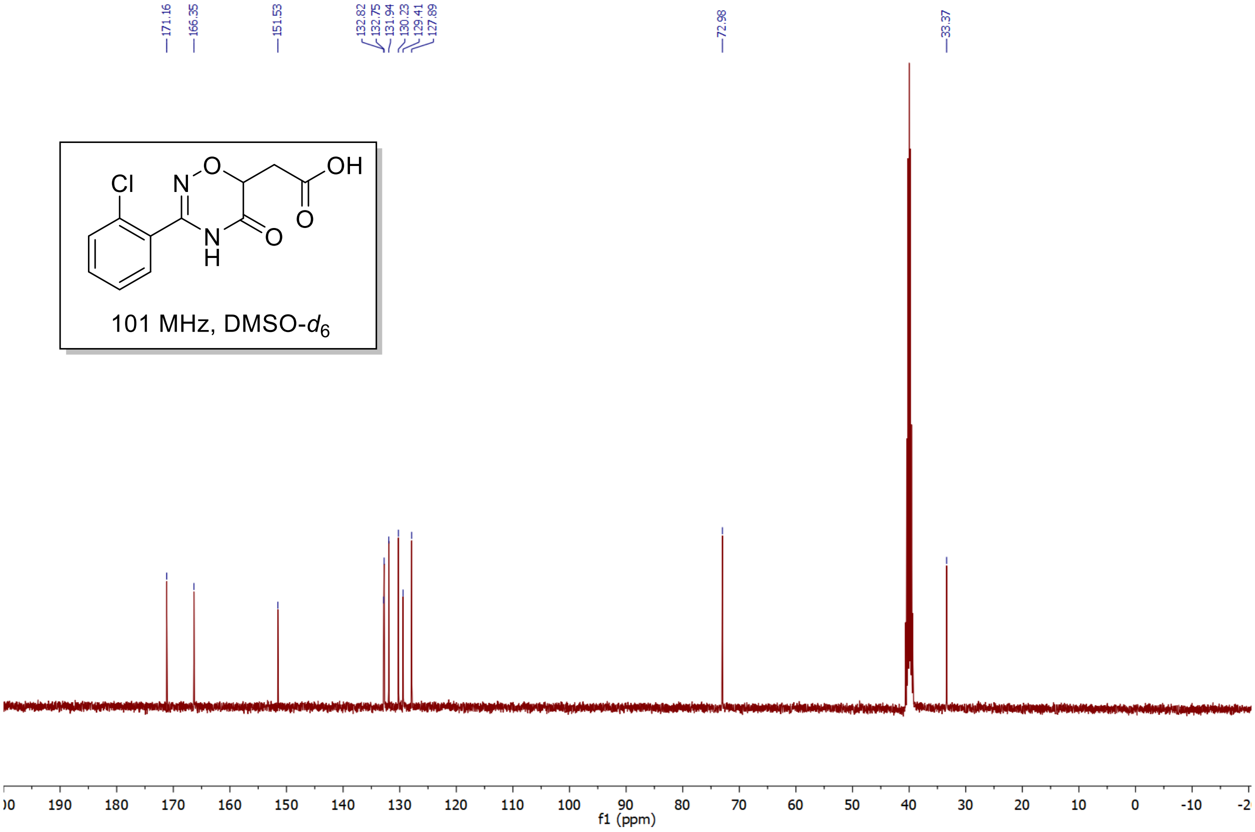

^{1}H and ^{13}C spectra of 2-(3-(3,4-Dichlorophenyl)-5-oxo-5,6-dihydro-4*H*-1,2,4-oxadiazin-6-yl)acetic acid **3l**

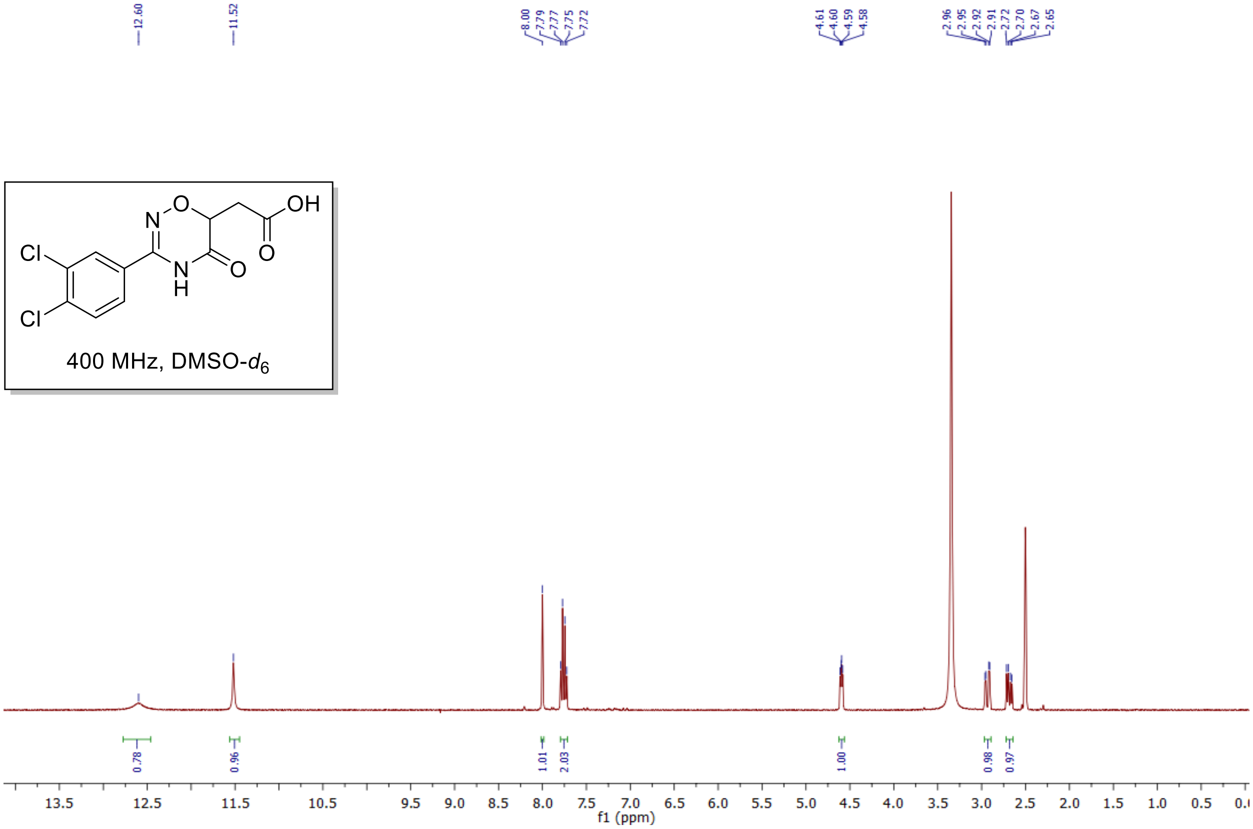

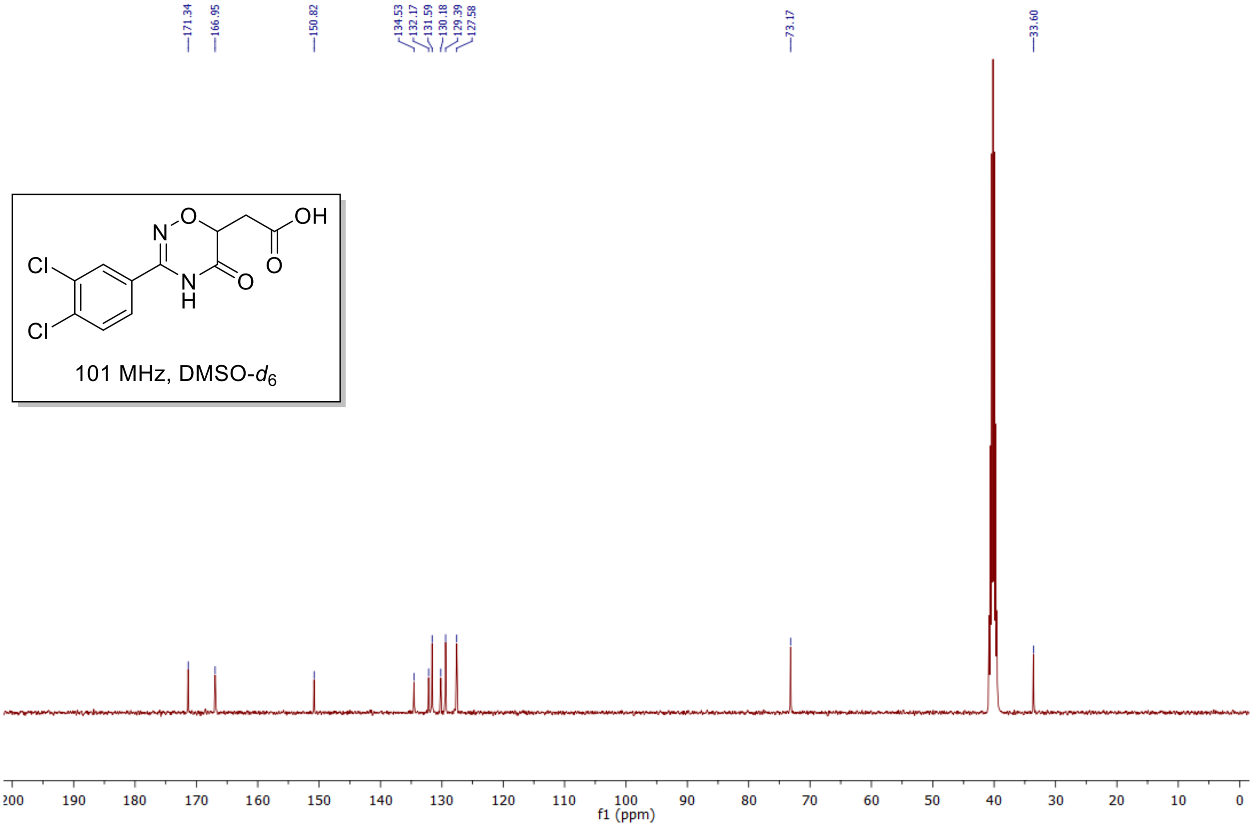

^{1}H and ^{13}C spectra of 2-(3-(3,5-dichlorophenyl)-5-oxo-5,6-dihydro-4*H*-1,2,4-oxadiazin-6-yl)acetic acid **3m**

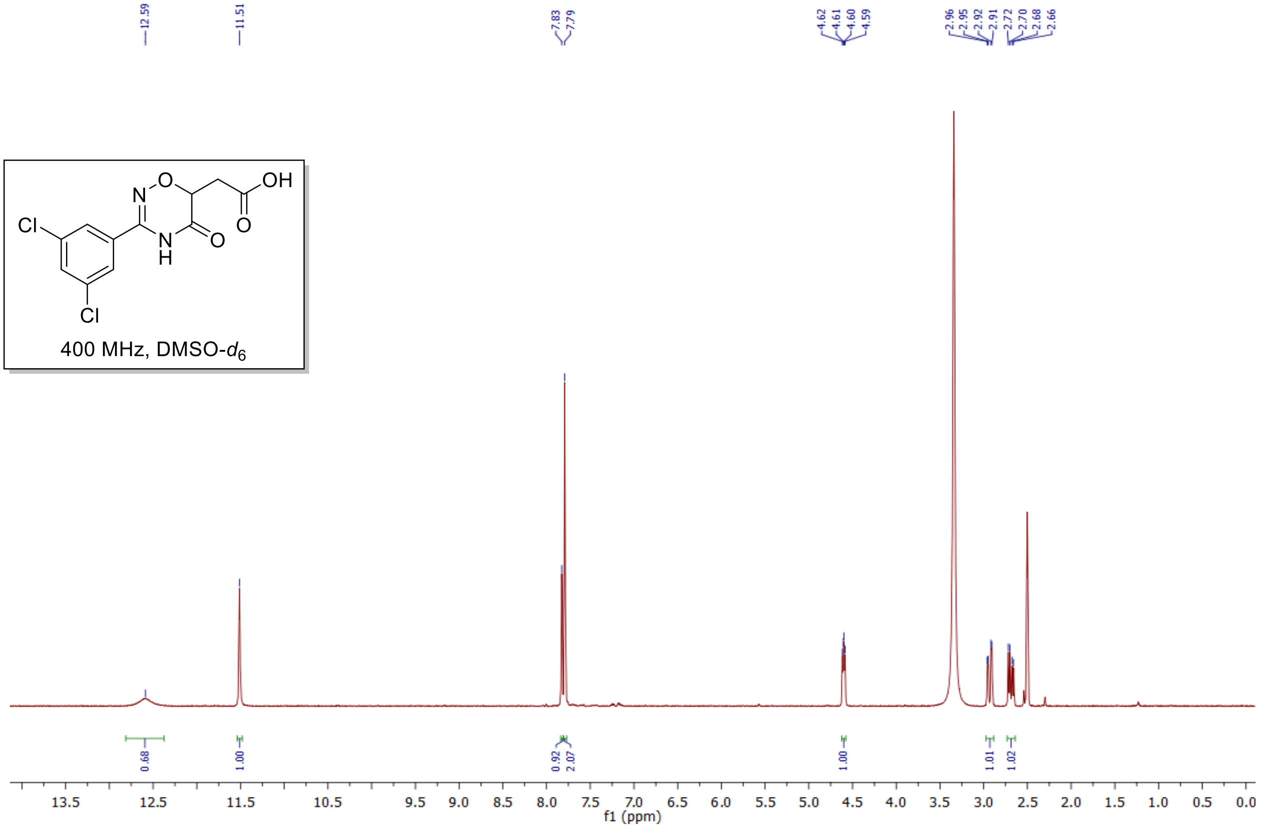

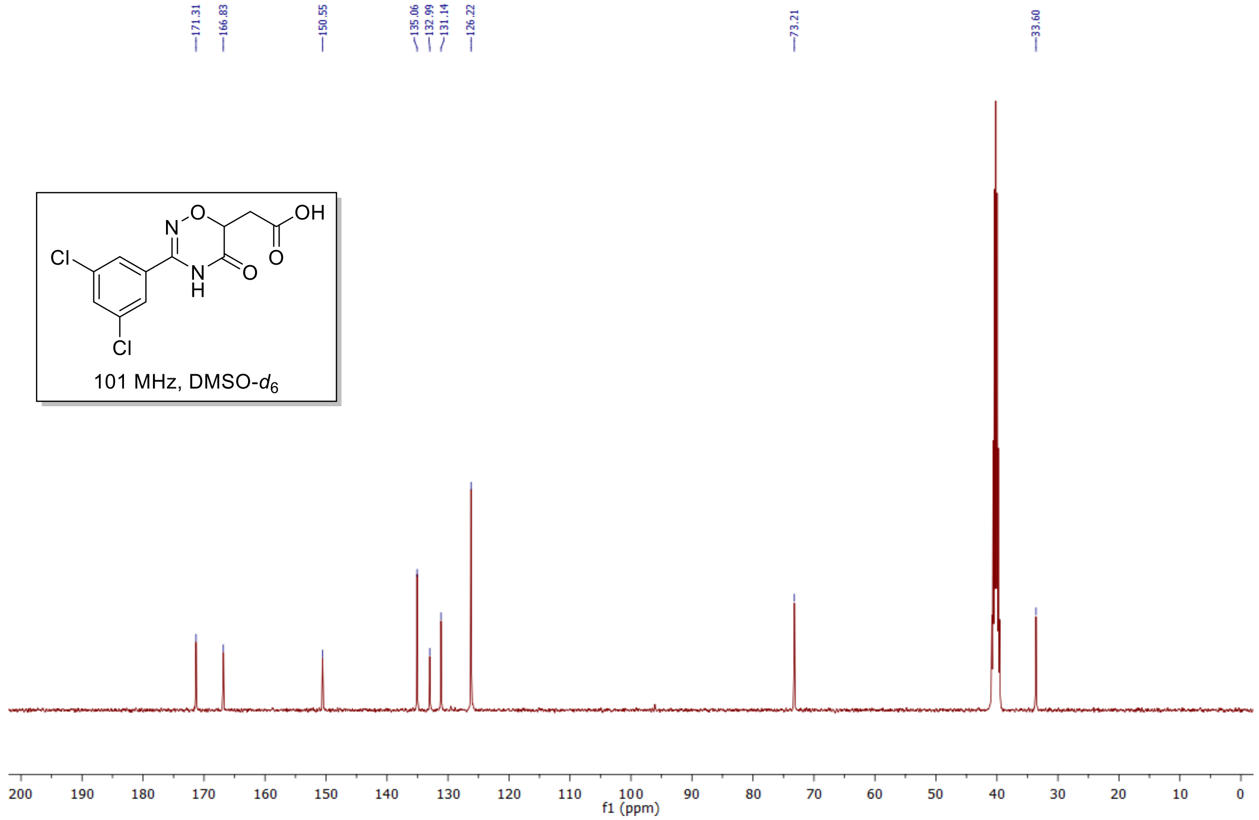

^{1}H and ^{13}C spectra of 2-(3-(5-methylthiophen-2-yl)-5-oxo-5,6-dihydro-4*H*-1,2,4-oxadiazin-6-yl)acetic acid **3n**

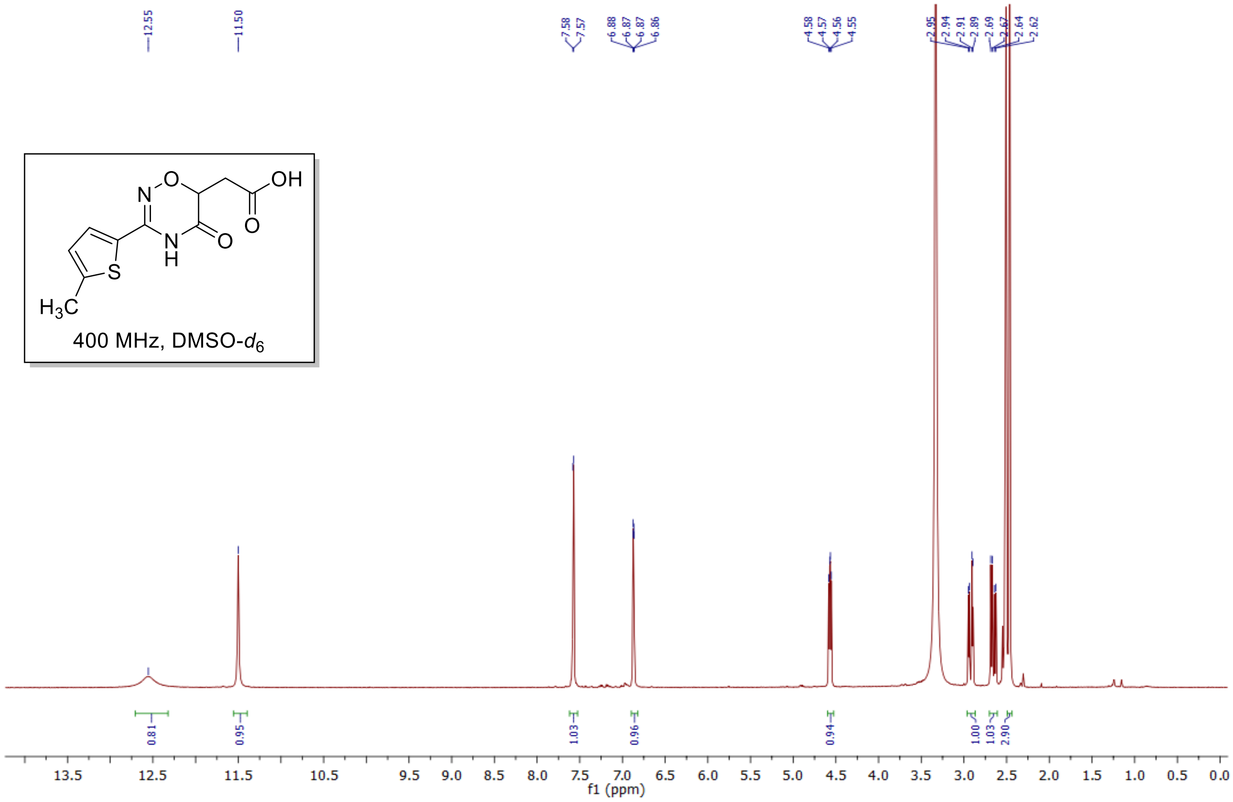

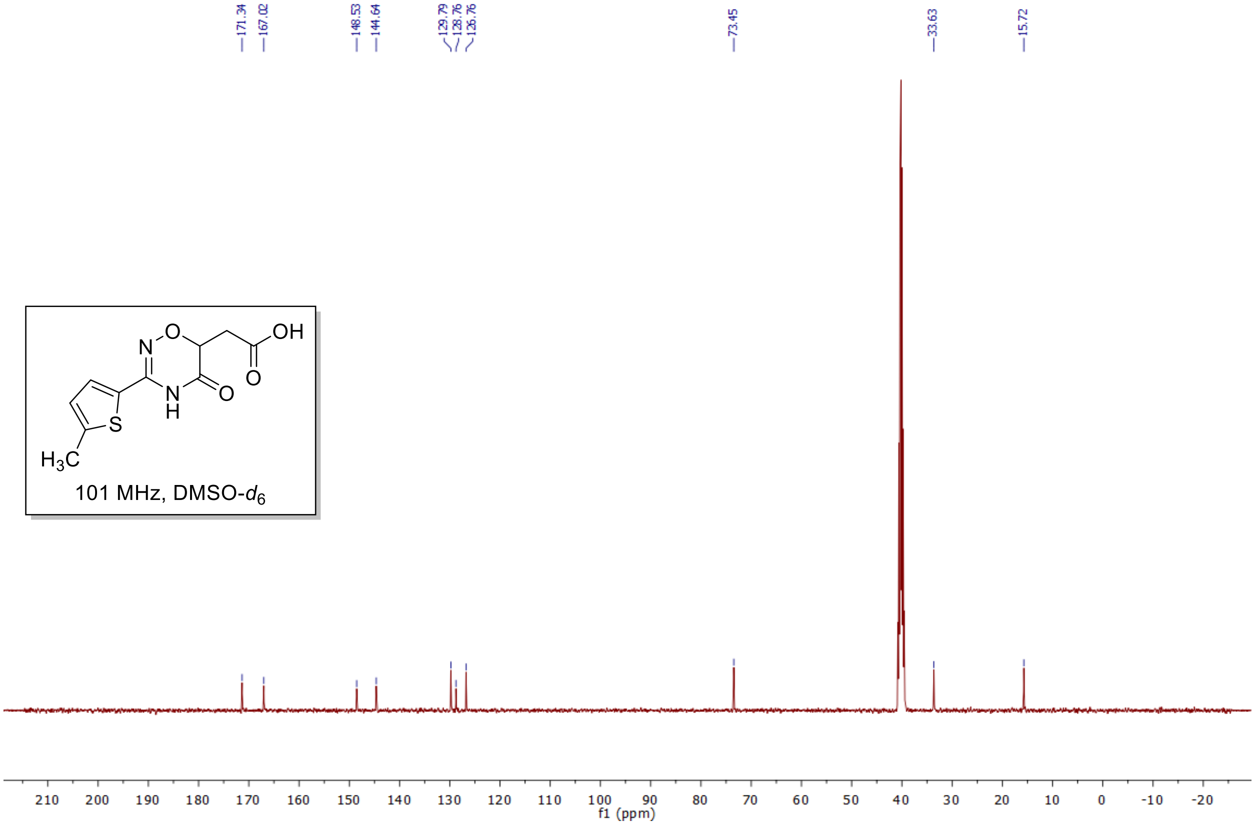

^{1}H and ^{13}C spectra of methyl 2-(3-(4-methylphenyl)-5-oxo-5,6-dihydro-4*H*-1,2,4-oxadiazin-6-yl)acetate **4a**

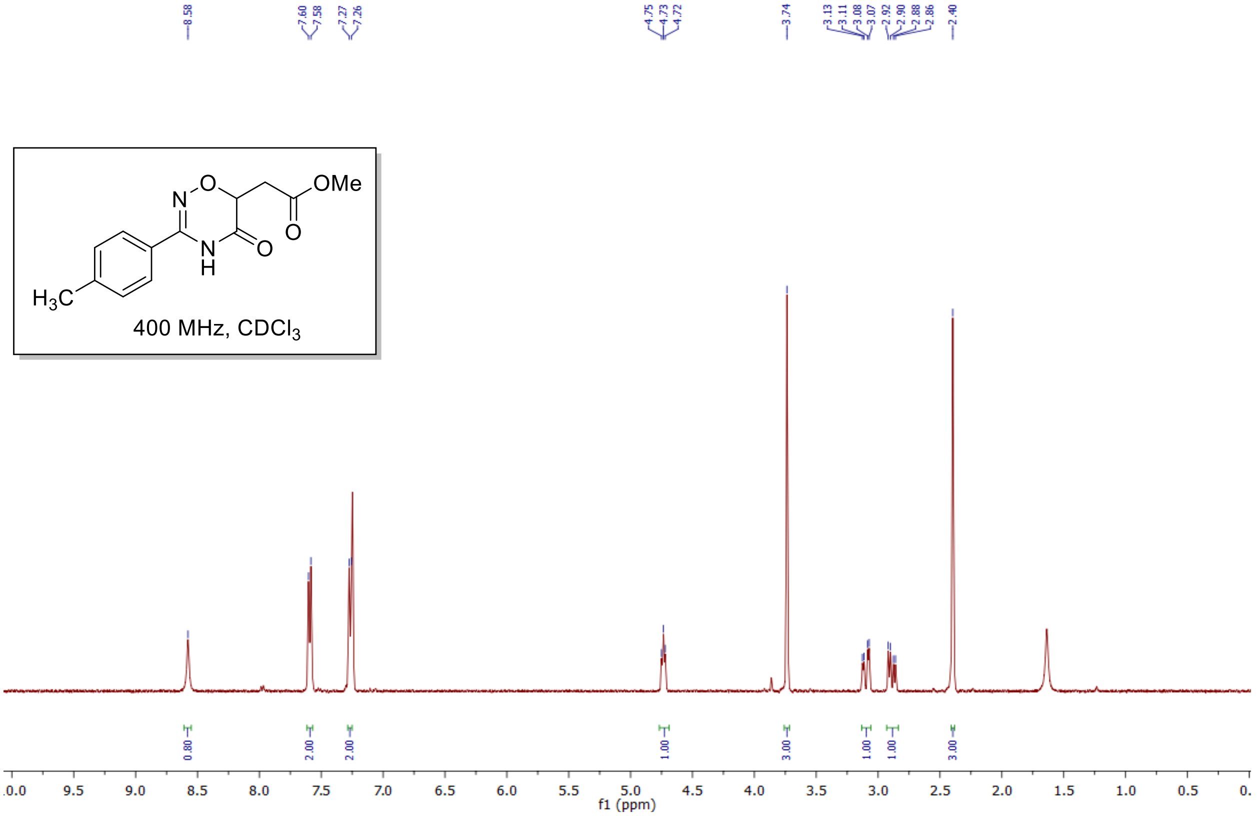

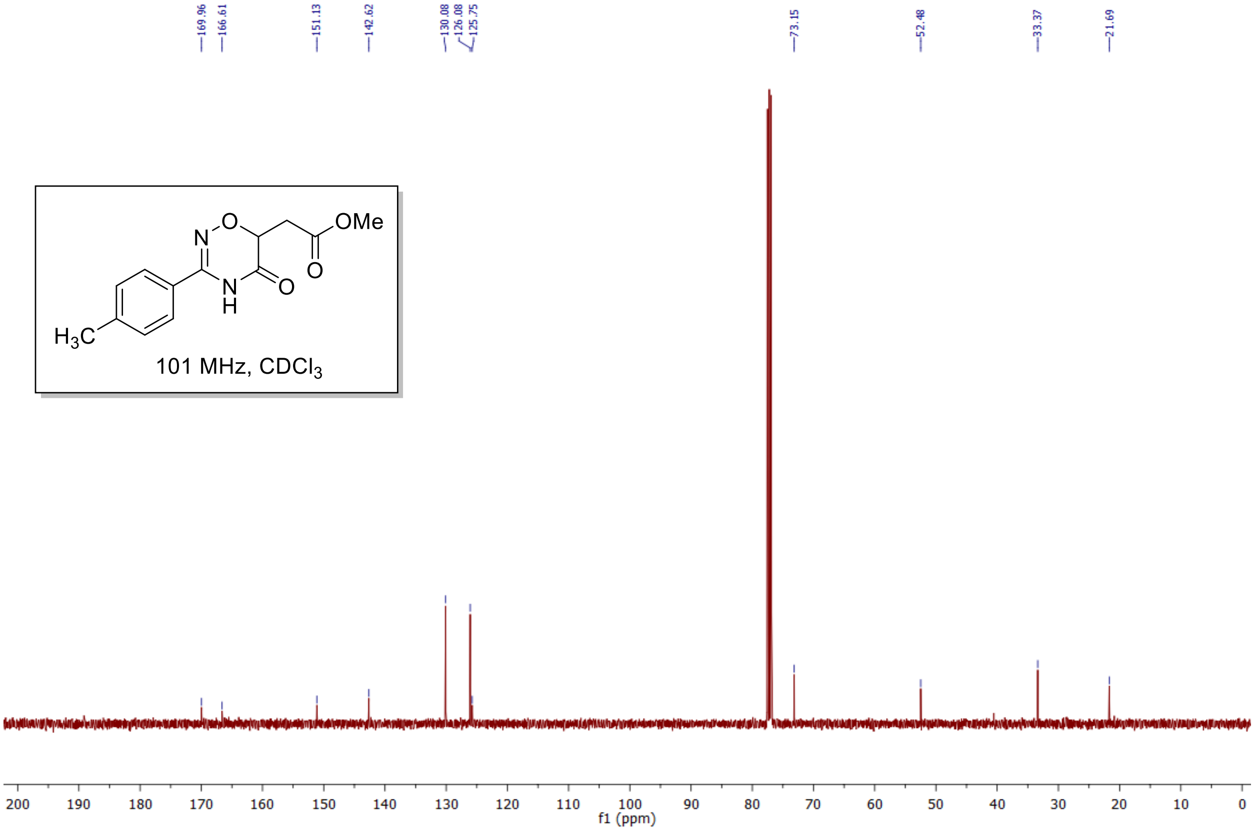

^{1}H and ^{13}C spectra of methyl 2-(5-oxo-3-phenyl-5,6-dihydro-4*H*-1,2,4-oxadiazin-6-yl)acetate **4b**

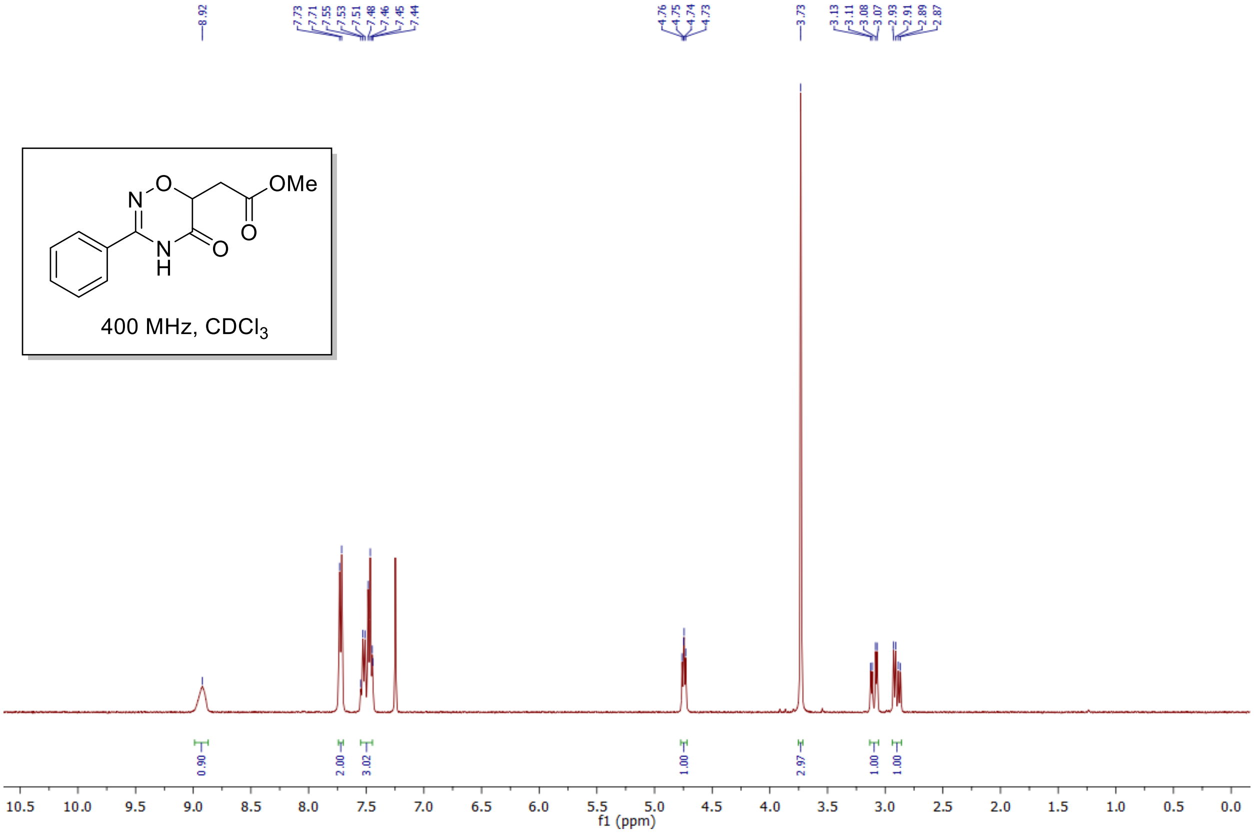

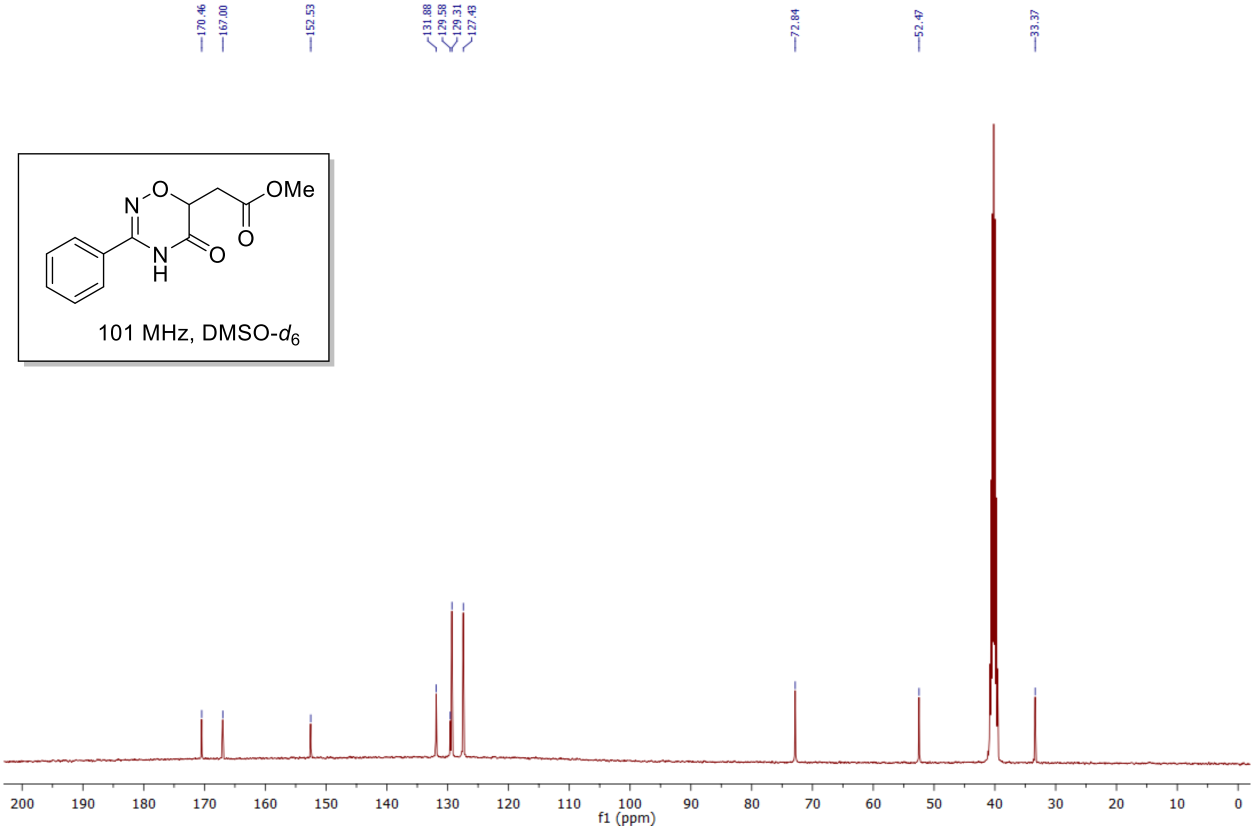

^{1}H and ^{13}C spectra of methyl 2-(3-(4-methoxyphenyl)-5-oxo-5,6-dihydro-4*H*-1,2,4-oxadiazin-6-yl)acetate **4c**

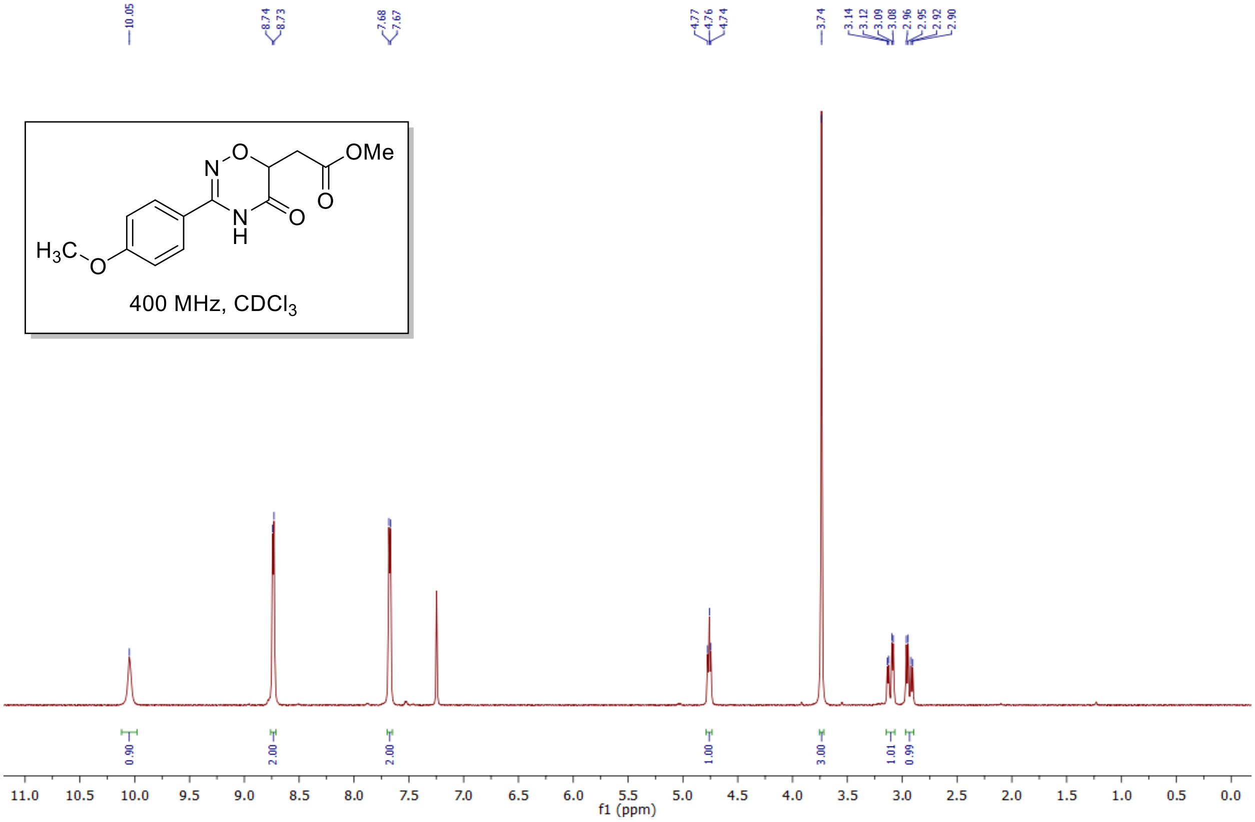

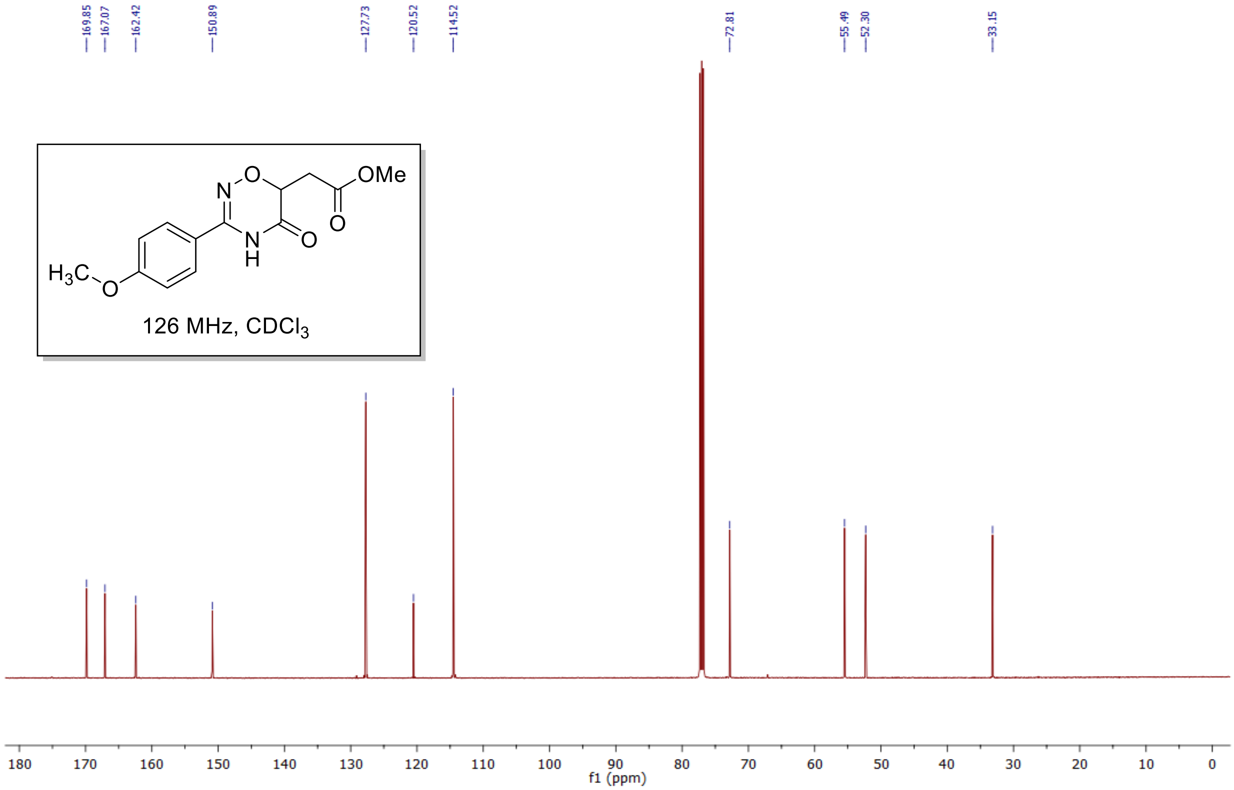

^{1}H and ^{13}C spectra of methyl 2-(3-(4-nitrophenyl)-5-oxo-5,6-dihydro-4*H*-1,2,4-oxadiazin-6-yl)acetate **4d**

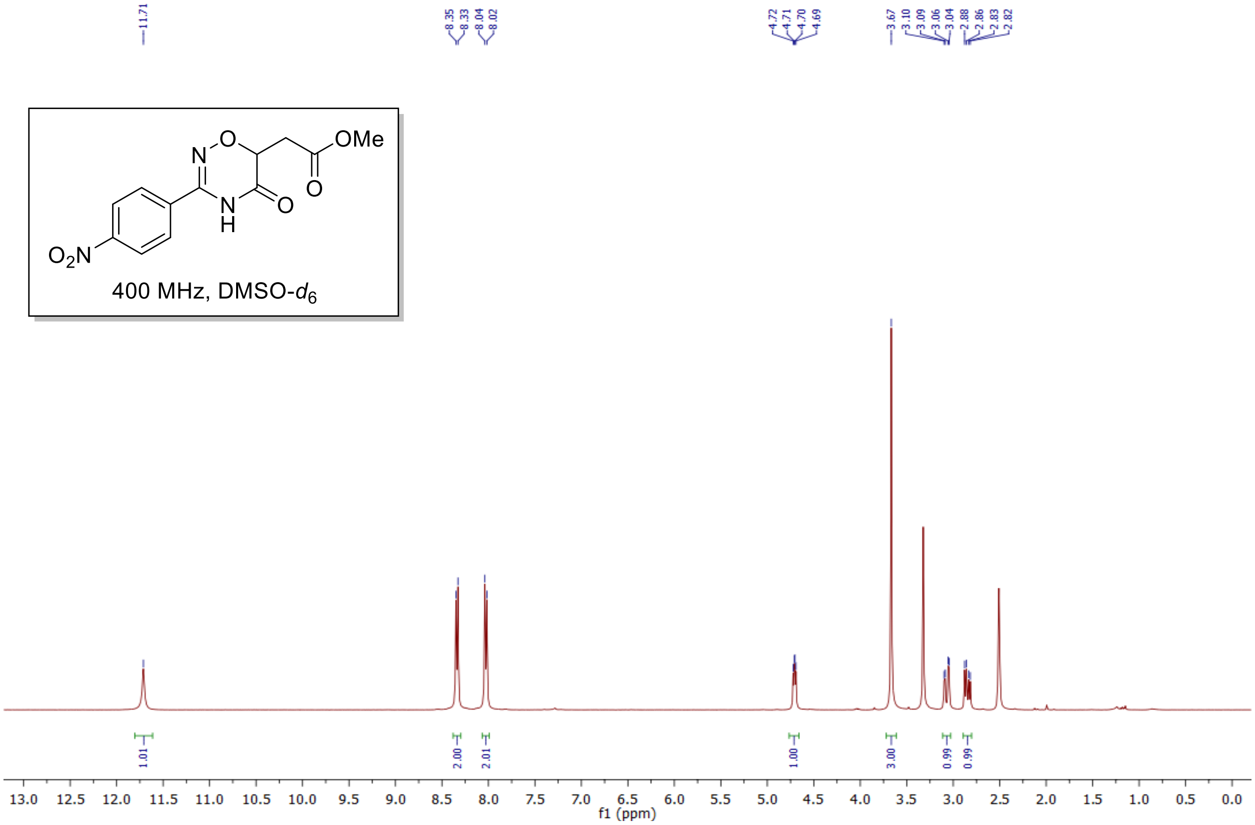

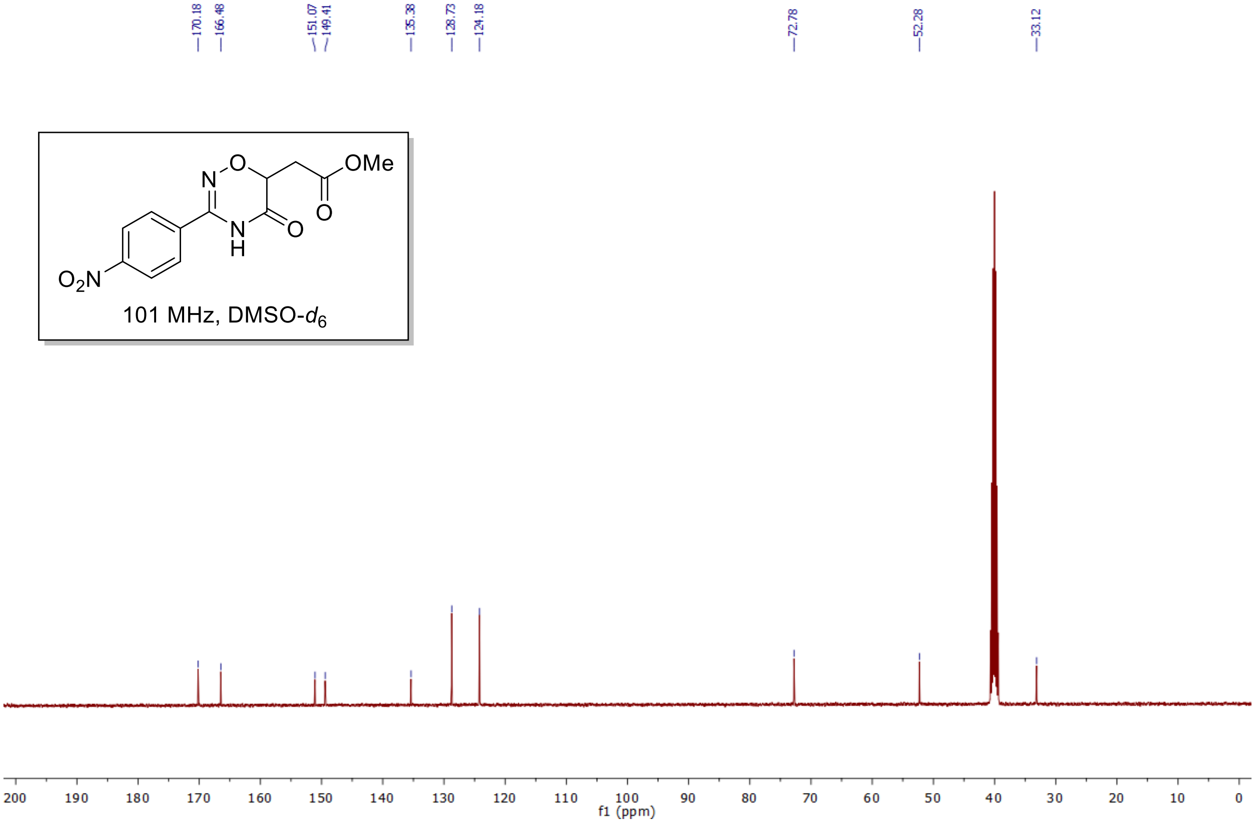

^{1}H, ^{13}C and ^{19}F spectra of methyl 2-(5-oxo-3-(4-(trifluoromethyl)phenyl)-5,6-dihydro-4*H*-1,2,4-oxadiazin-6-yl)acetate **4e**

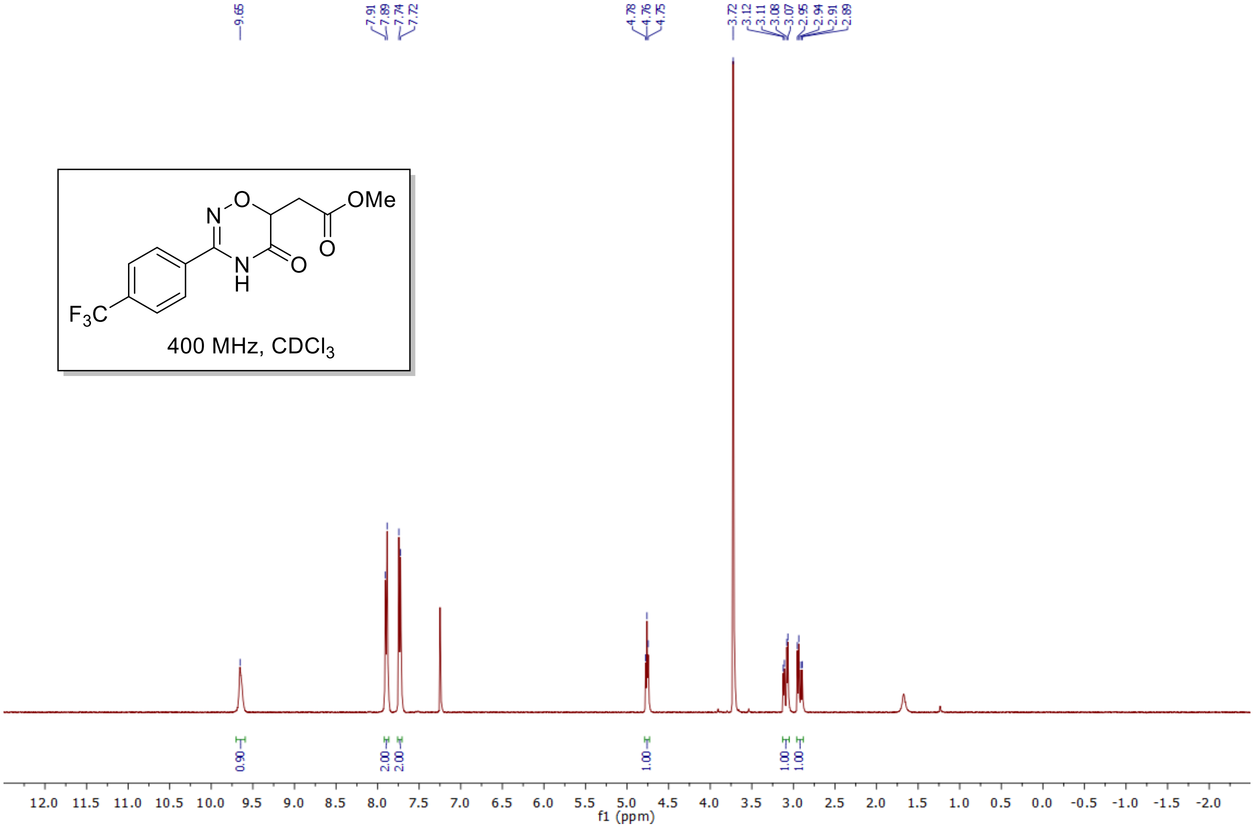

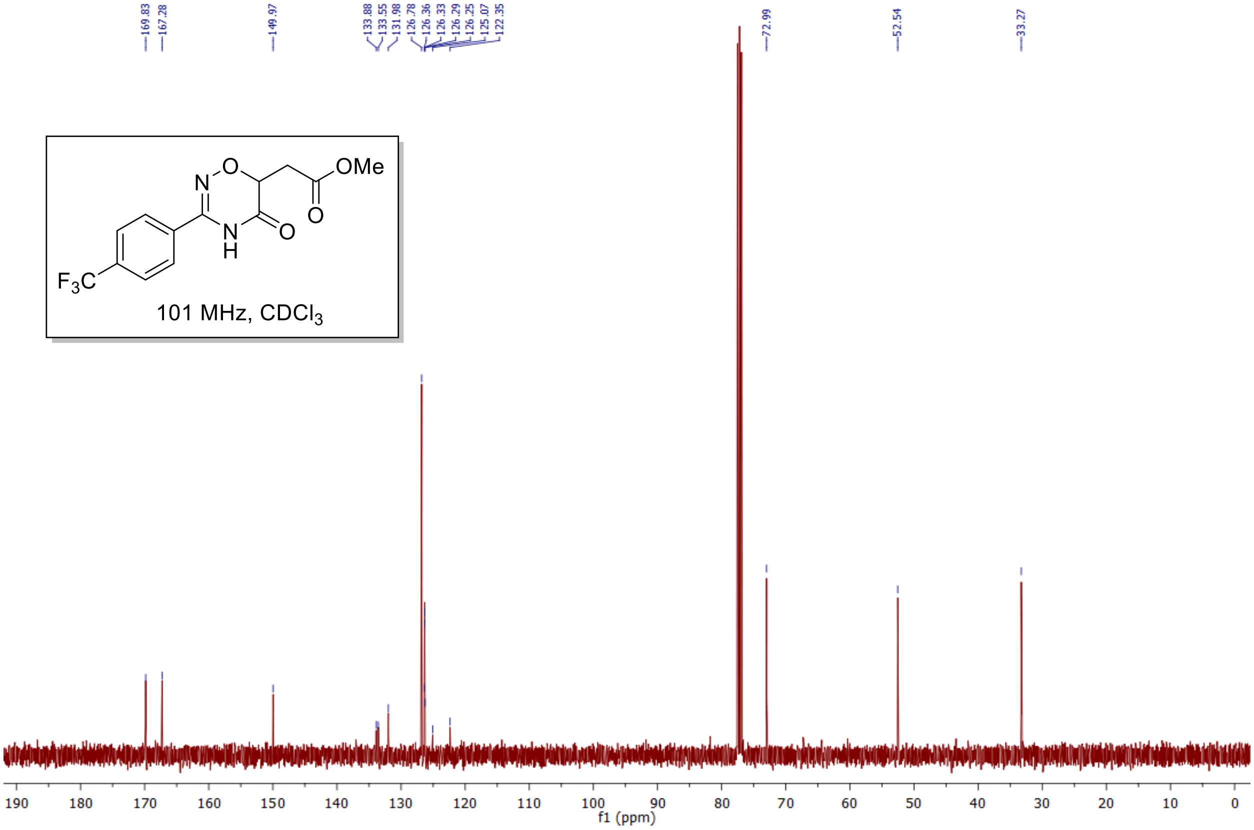

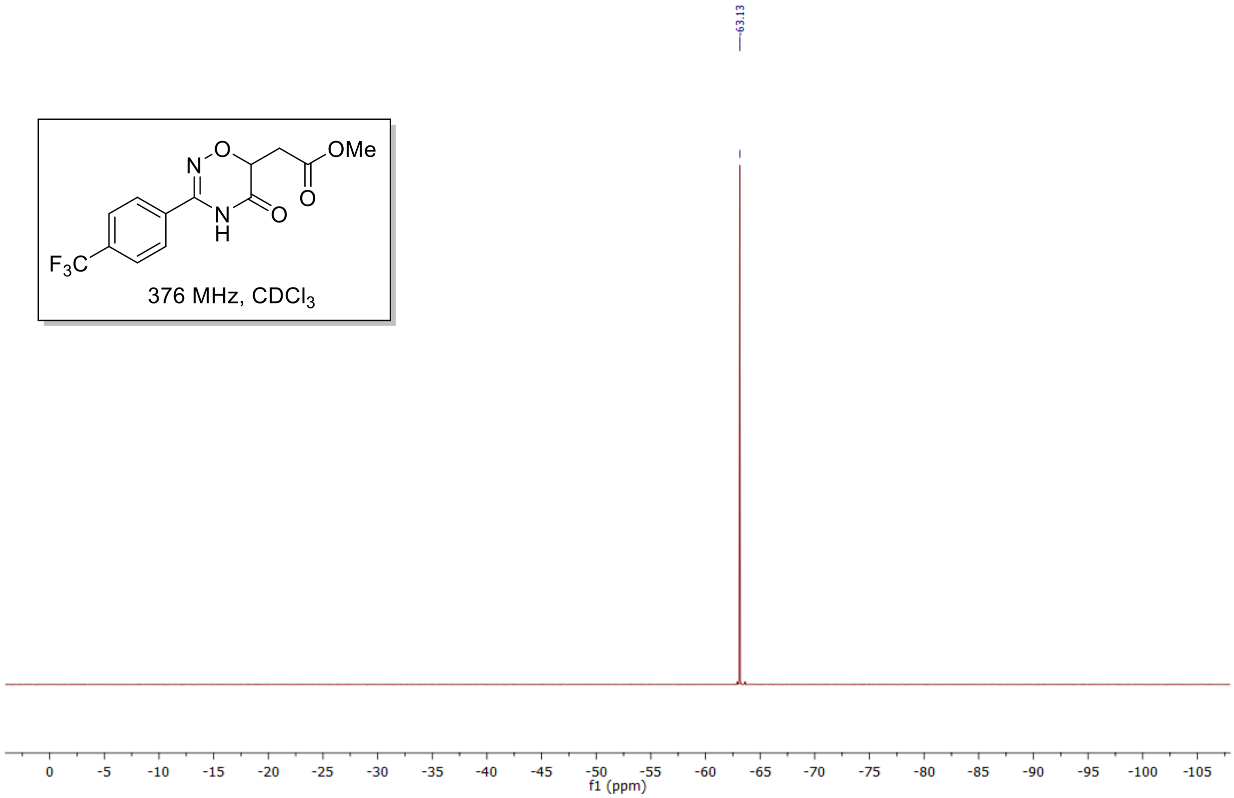

-63.13
OMe
O
N
O
N
H
O
F3C
376 MHz, CDCl3
0
-5
-10
-15
-20
-25
-30
-35
-40
-45
-50
-55
-60
-65
-70
-75
-80
-85
-90
-95
-100
-105
f1 (ppm)

^{1}H, ^{13}C and ^{19}F spectra of methyl 2-(3-(4-fluorophenyl)-5-oxo-5,6-dihydro-4*H*-1,2,4-oxadiazin-6-yl)acetate **4f**

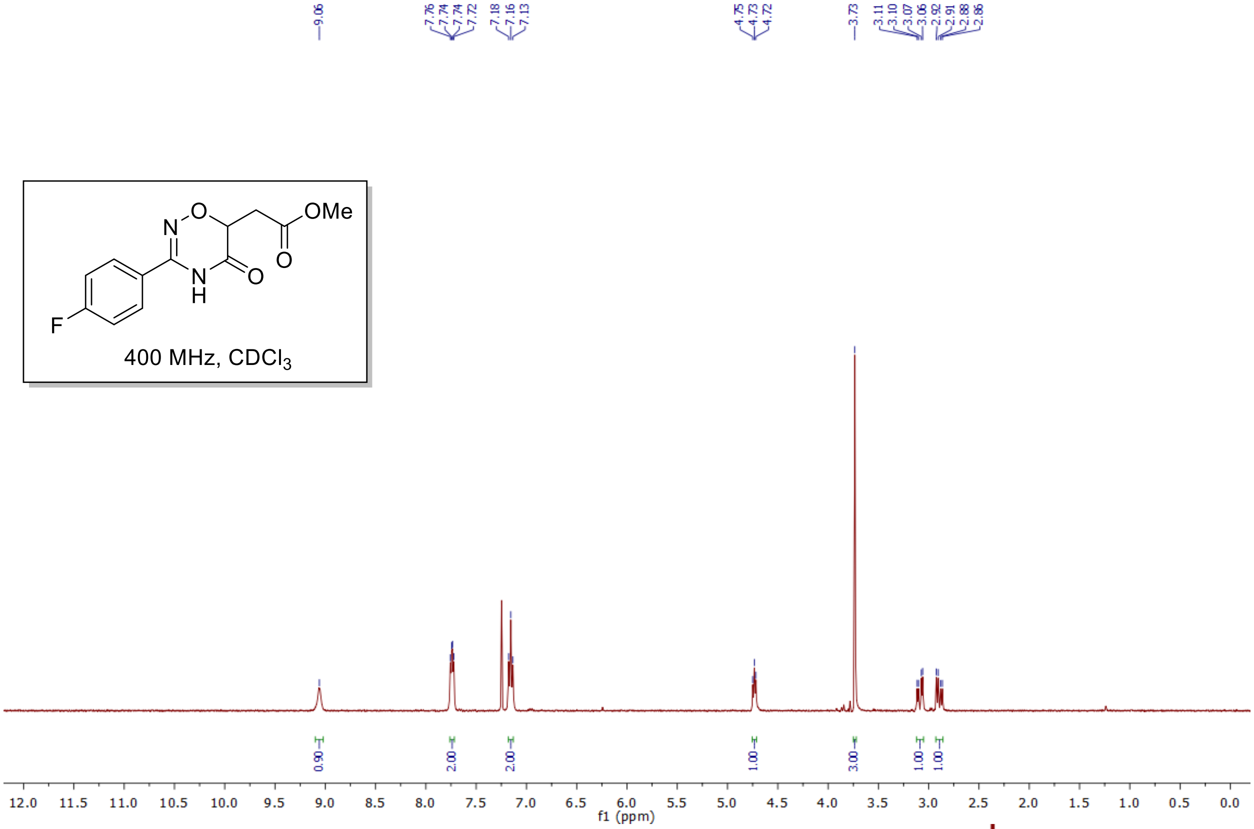

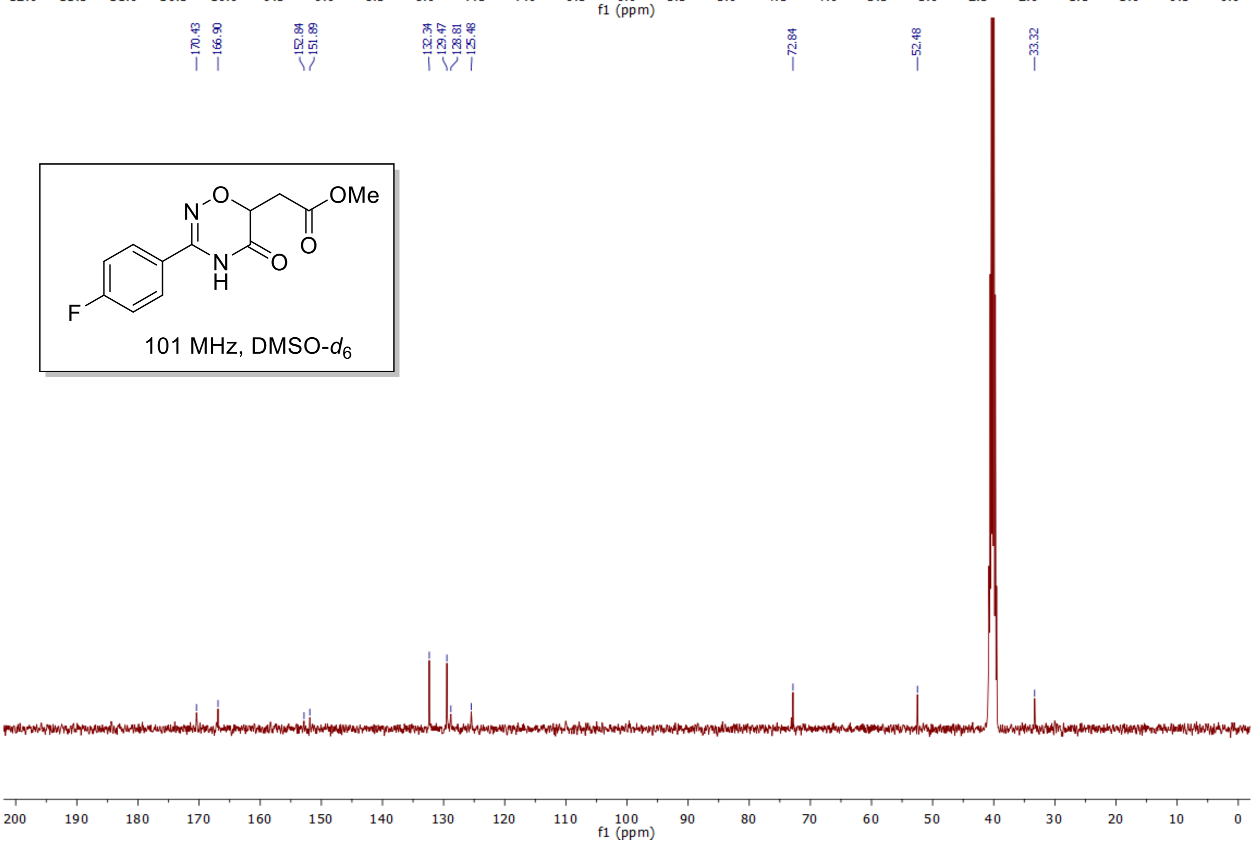

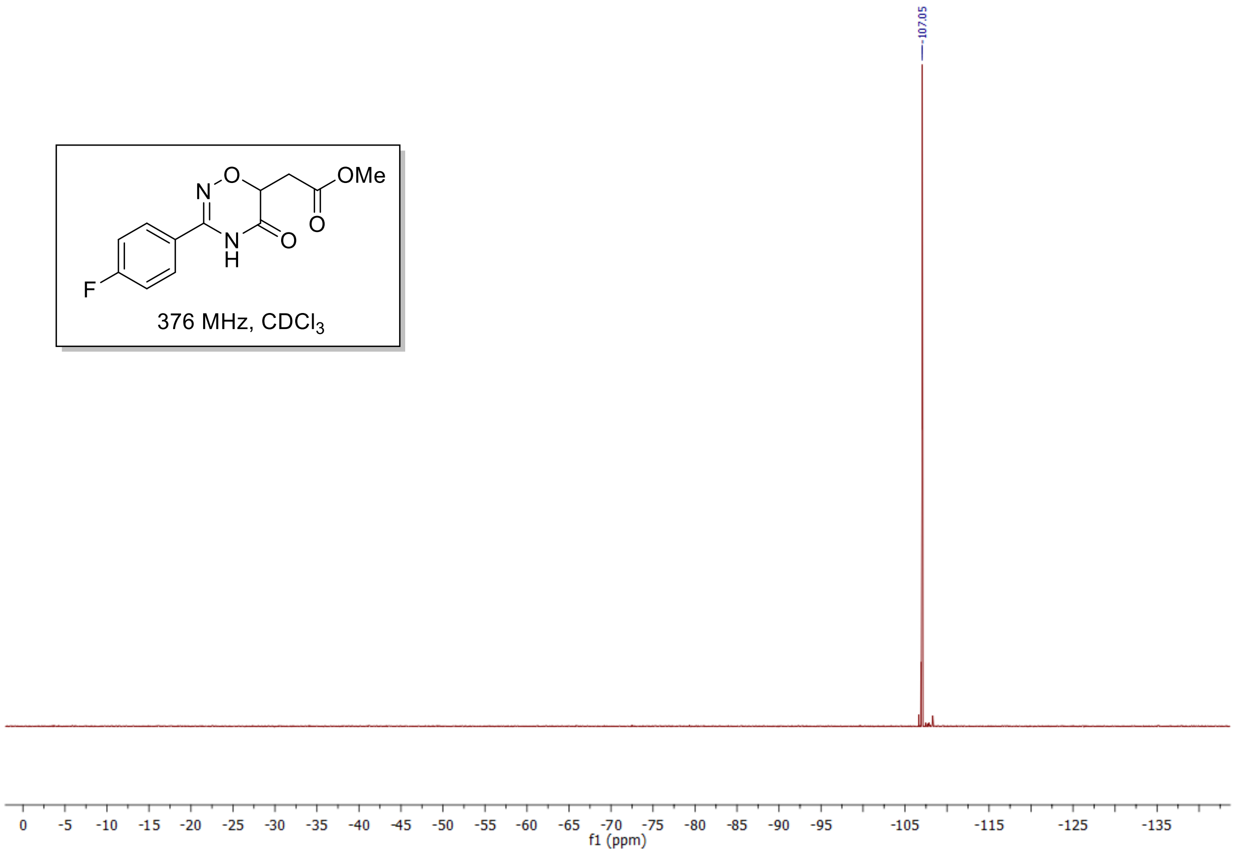
-107.05
OMe
376 MHz, CDCl3
0 -5 -10 -15 -20 -25 -30 -35 -40 -45 -50 -55 -60 -65 -70 -75 -80 -85 -90 -95 -105 -115 -125 -135
f1 (ppm)

^{1}H and ^{13}C spectra of methyl -(3-(4-bromophenyl)-5-oxo-5,6-dihydro-4*H*-1,2,4-oxadiazin-6-yl)acetate **4g**

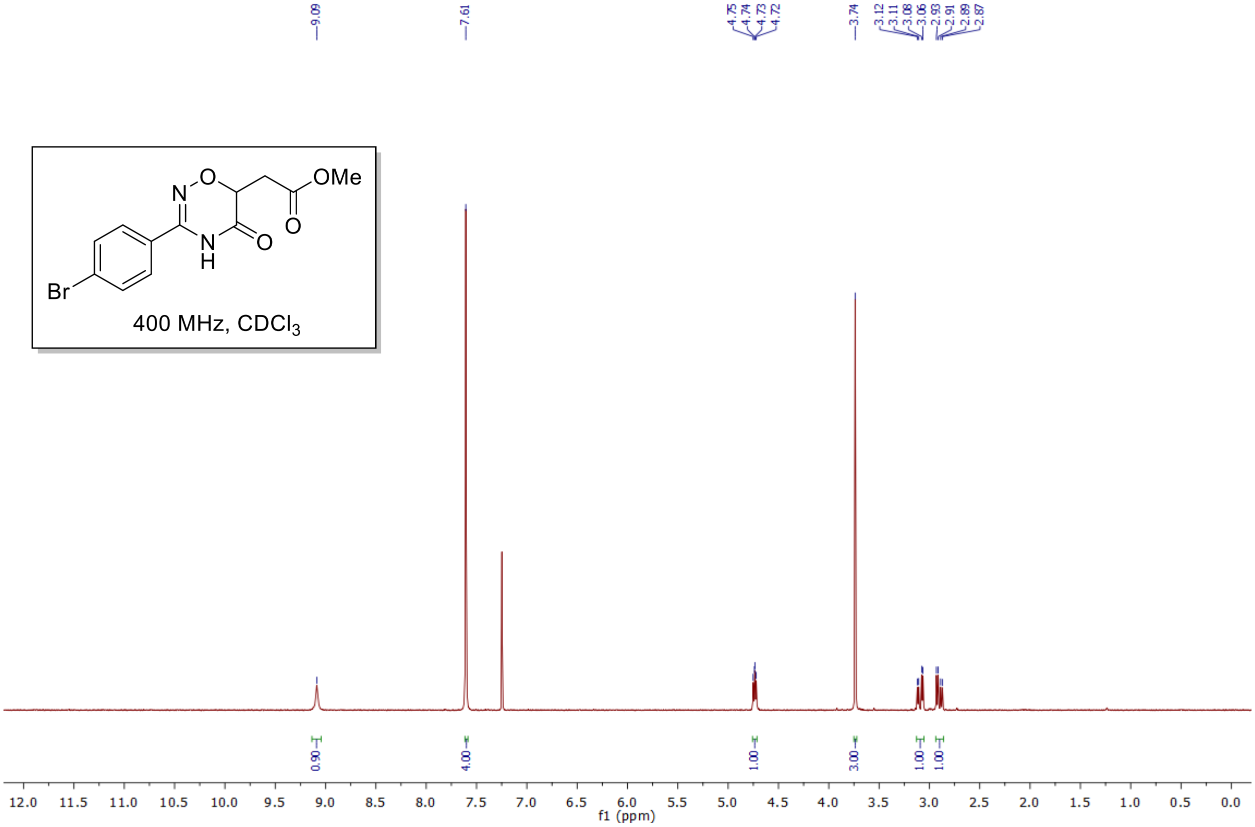

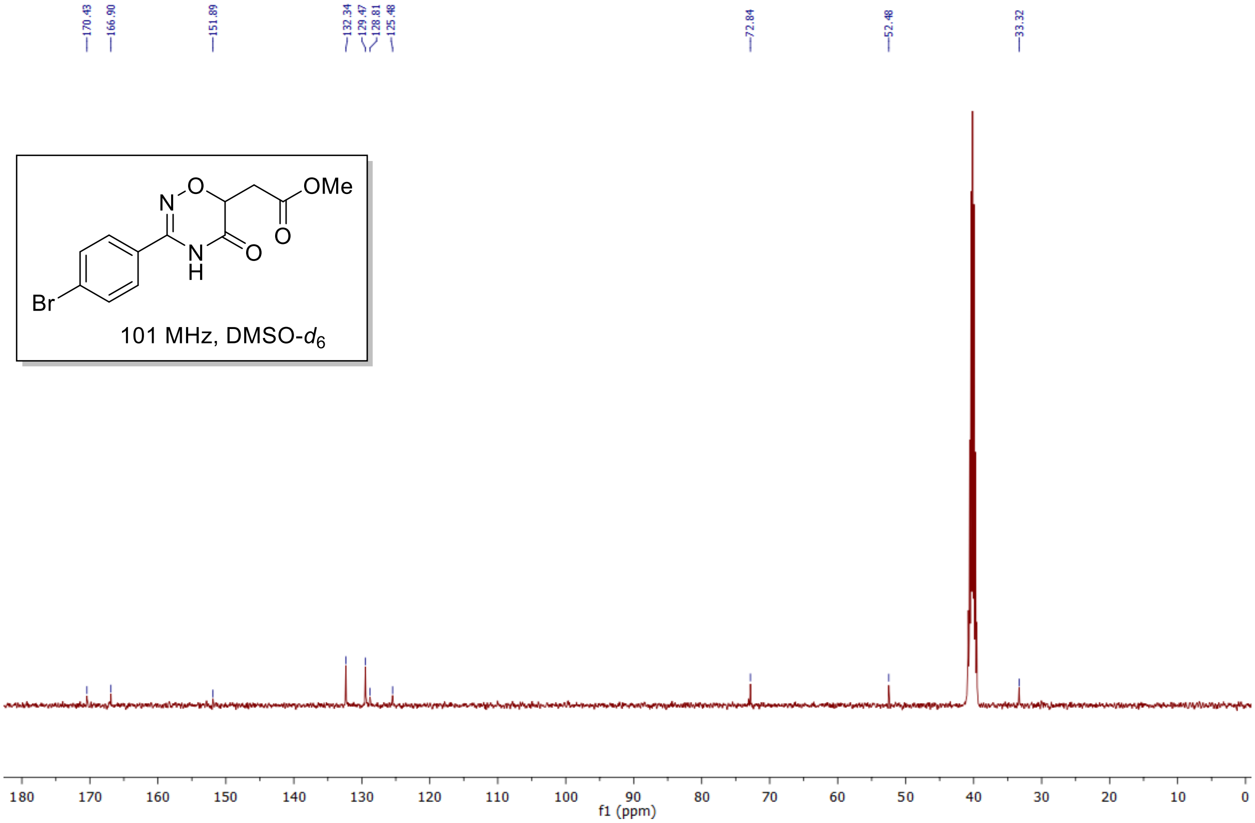

^{1}H and ^{13}C spectra of methyl 2-(3-(4-chlorophenyl)-5-oxo-5,6-dihydro-4*H*-1,2,4-oxadiazin-6-yl)acetate **4h**

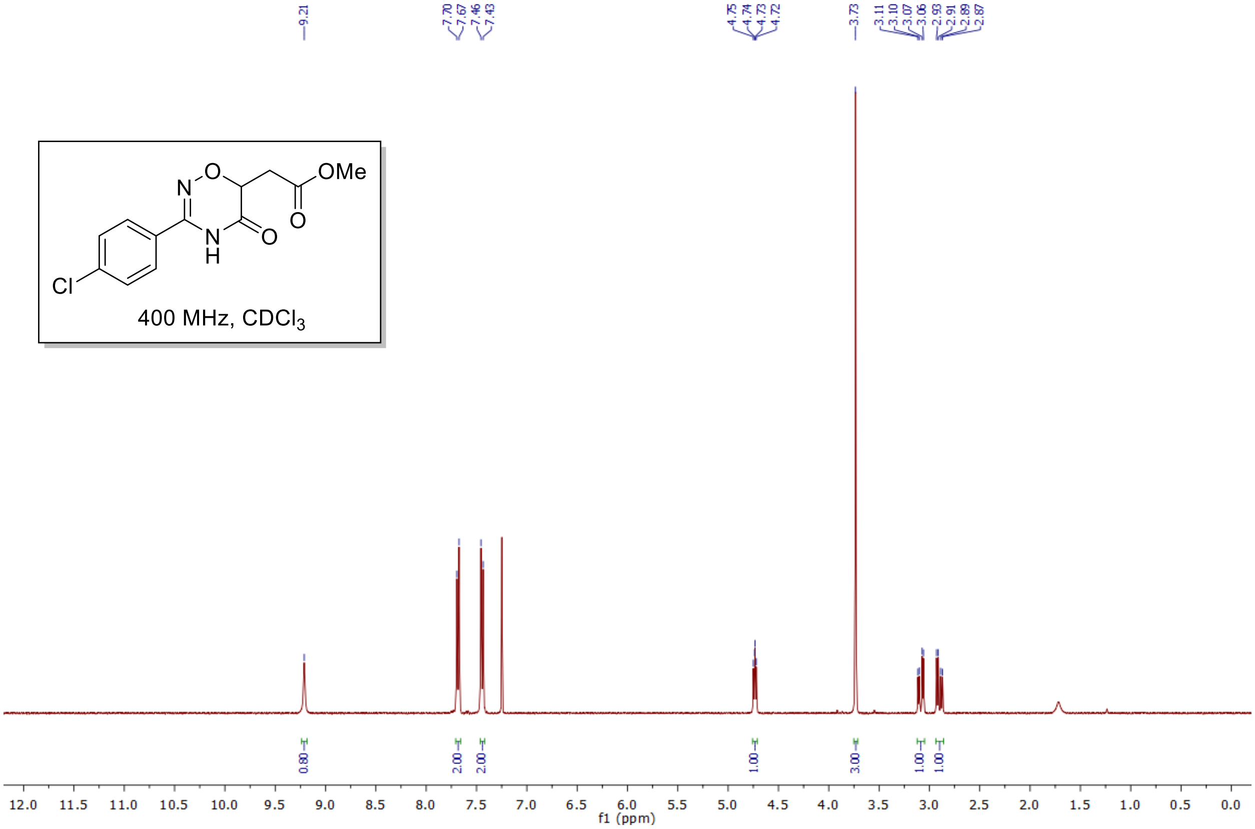

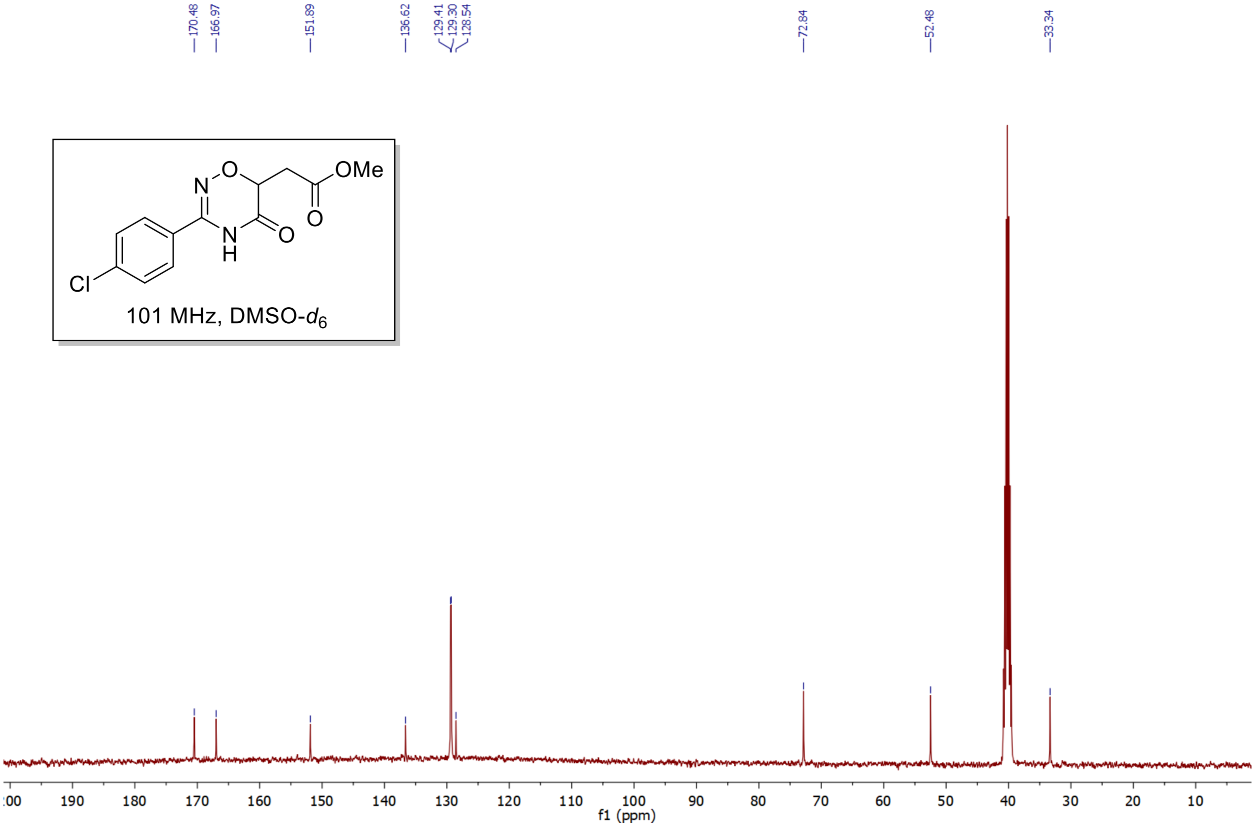

^{1}H and ^{13}C spectra of methyl 2-(3-(2-methylphenyl)-5-oxo-5,6-dihydro-4*H*-1,2,4-oxadiazin-6-yl)acetate **4i**

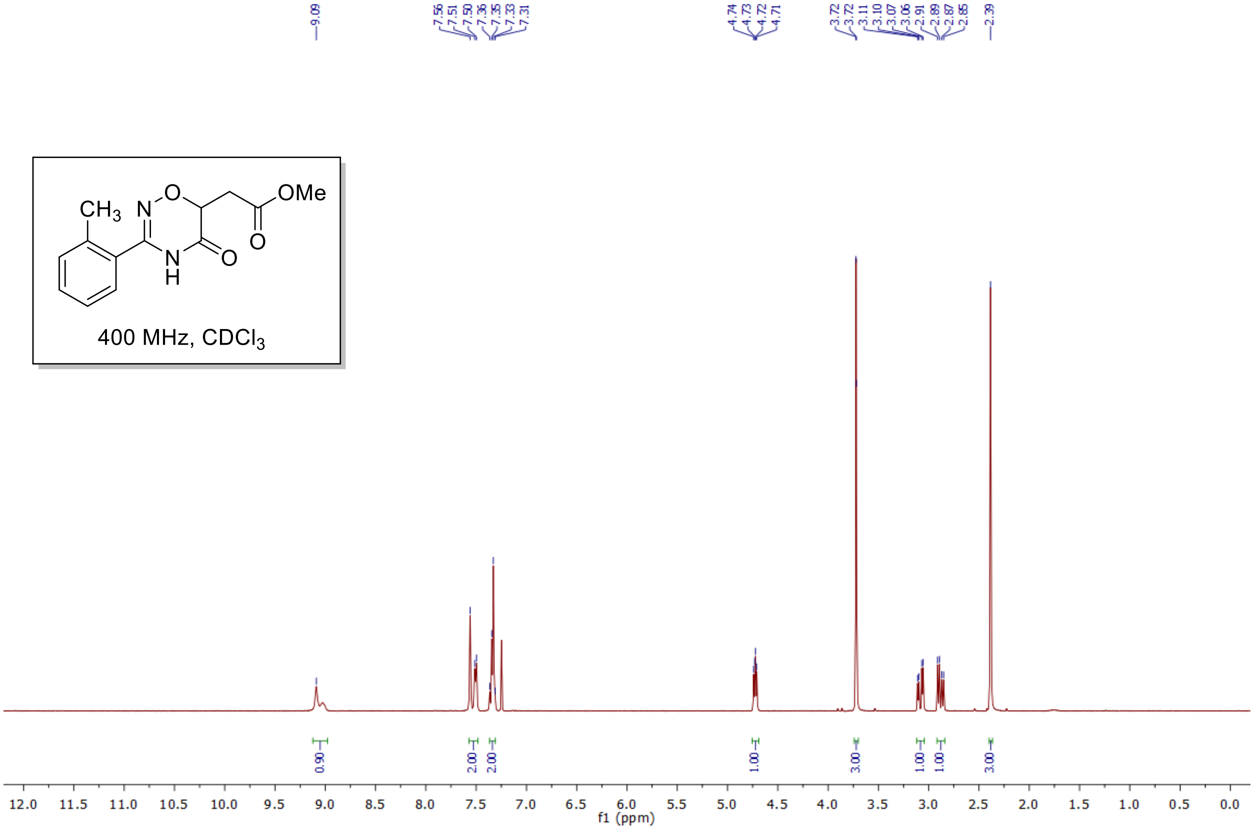

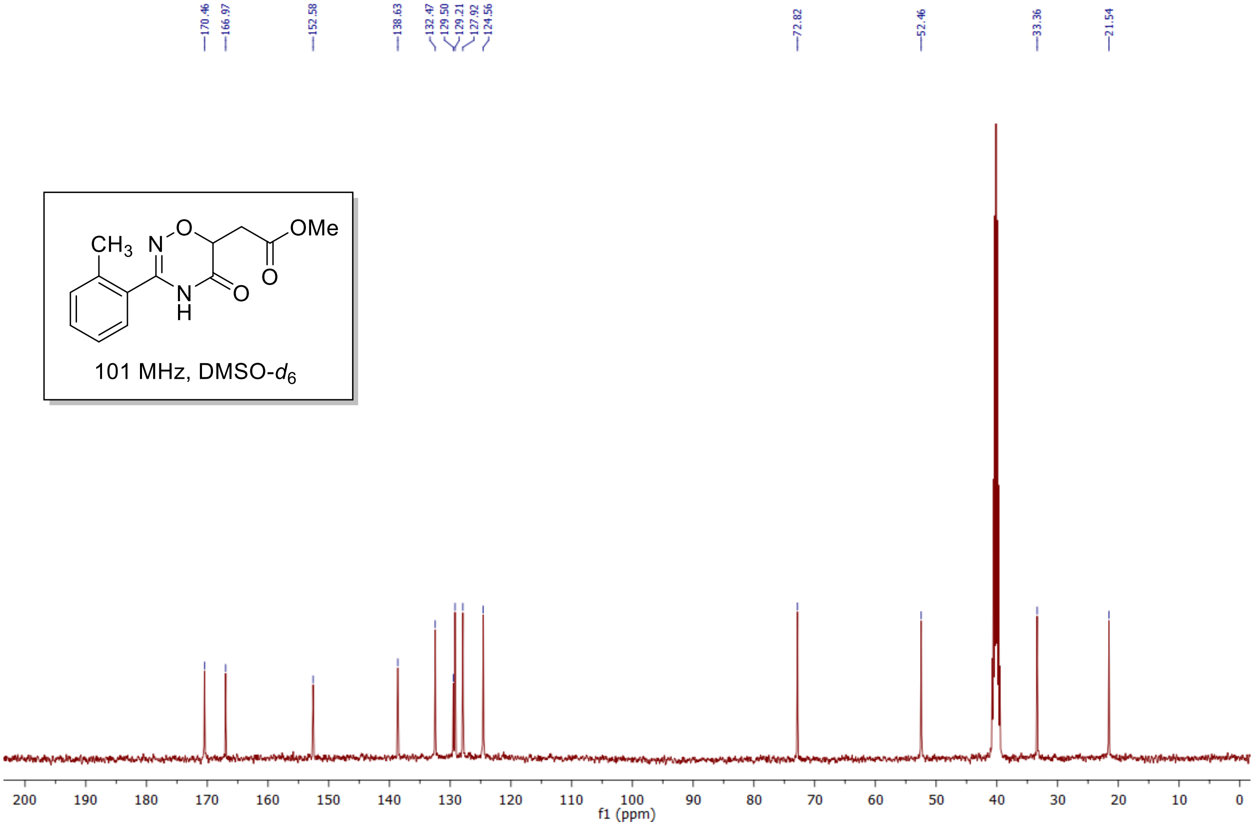

^{1}H and ^{13}C spectra of methyl 2-(3-(2-methoxyphenyl)-5-oxo-5,6-dihydro-4*H*-1,2,4-oxadiazin-6-yl)acetate **4j**

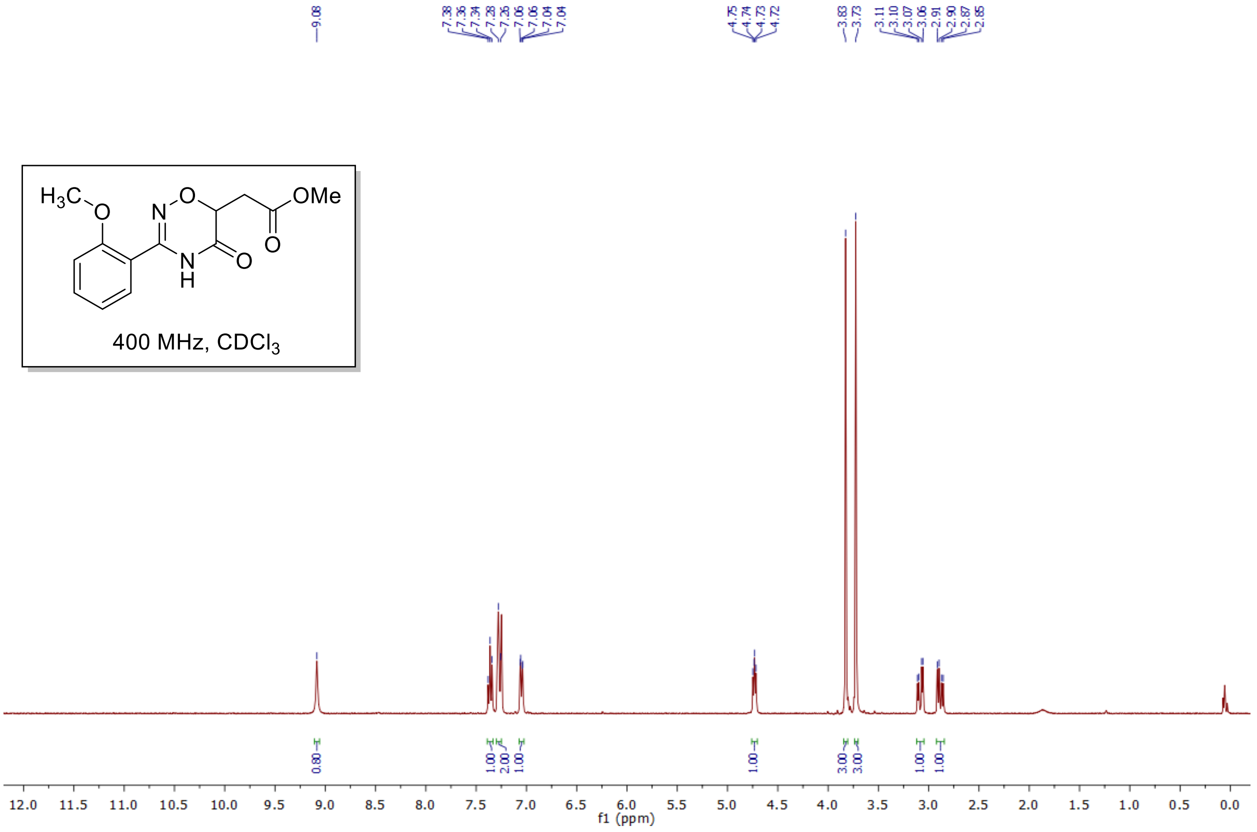

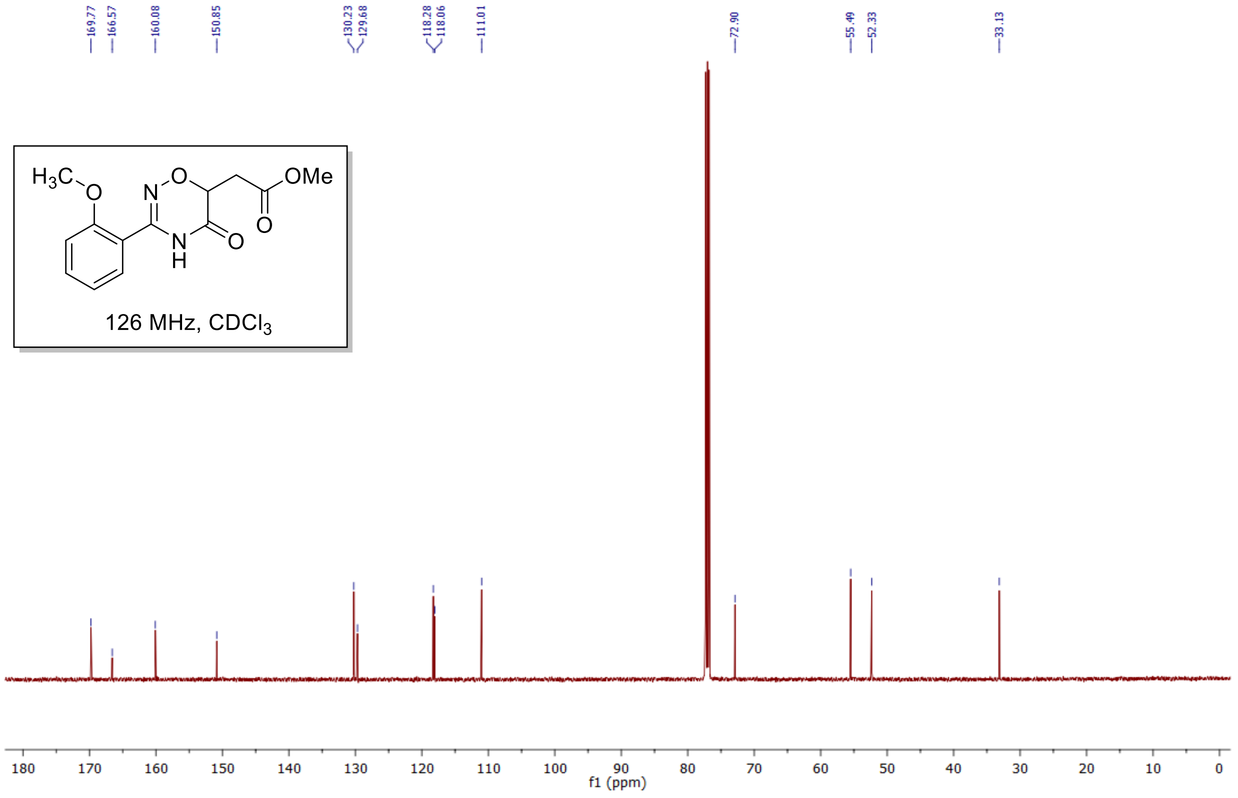

^{1}H and ^{13}C spectra of methyl 2-(3-(2-chlorophenyl)-5-oxo-5,6-dihydro-4*H*-1,2,4-oxadiazin-6-yl)acetate **4k**

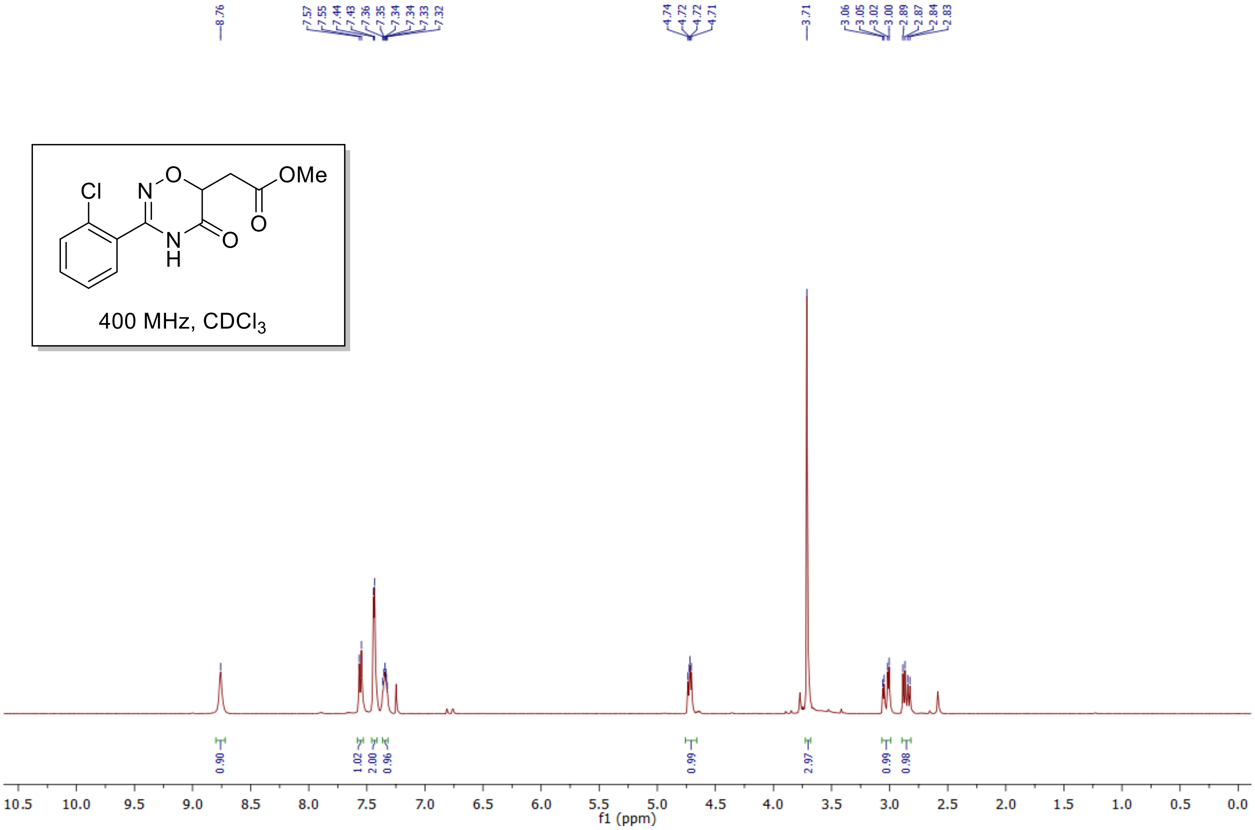

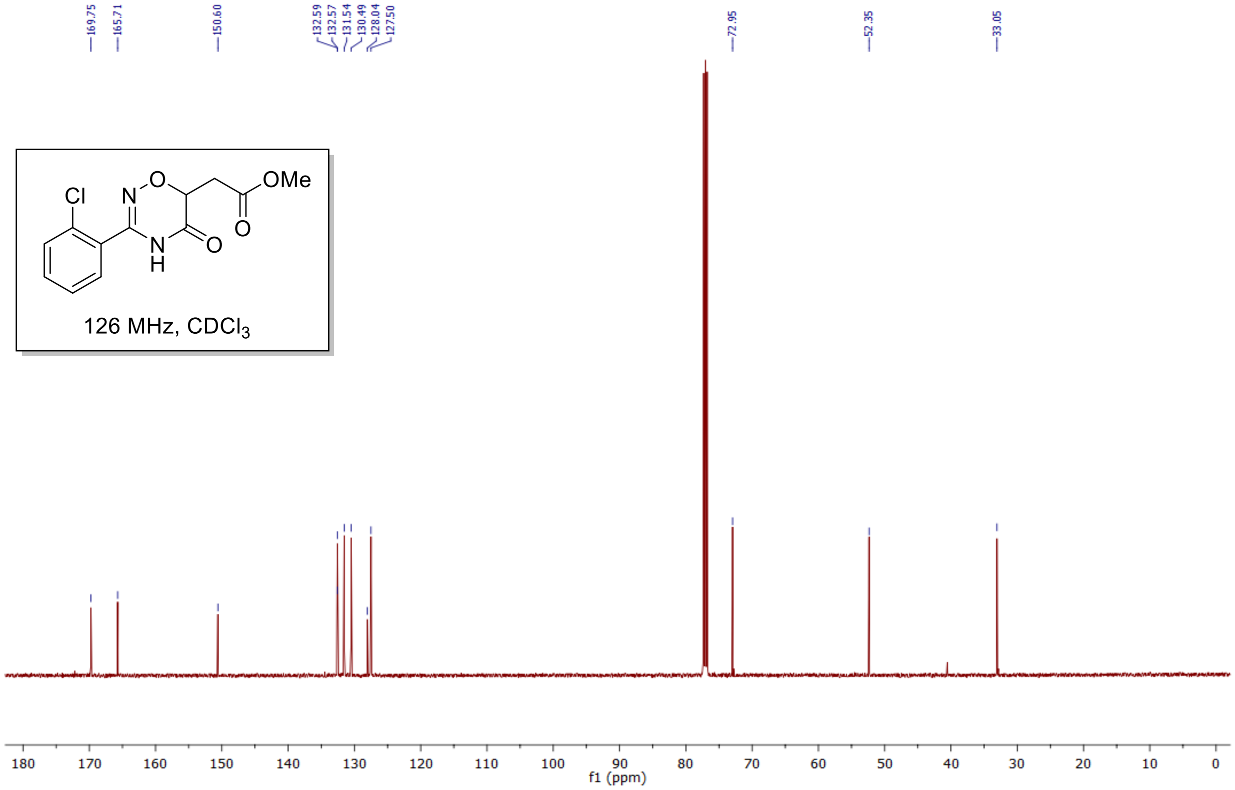

^{1}H and ^{13}C spectra of methyl 2-(3-(3,4-dichlorophenyl)-5-oxo-5,6-dihydro-4*H*-1,2,4-oxadiazin-6-yl)acetate **4l**

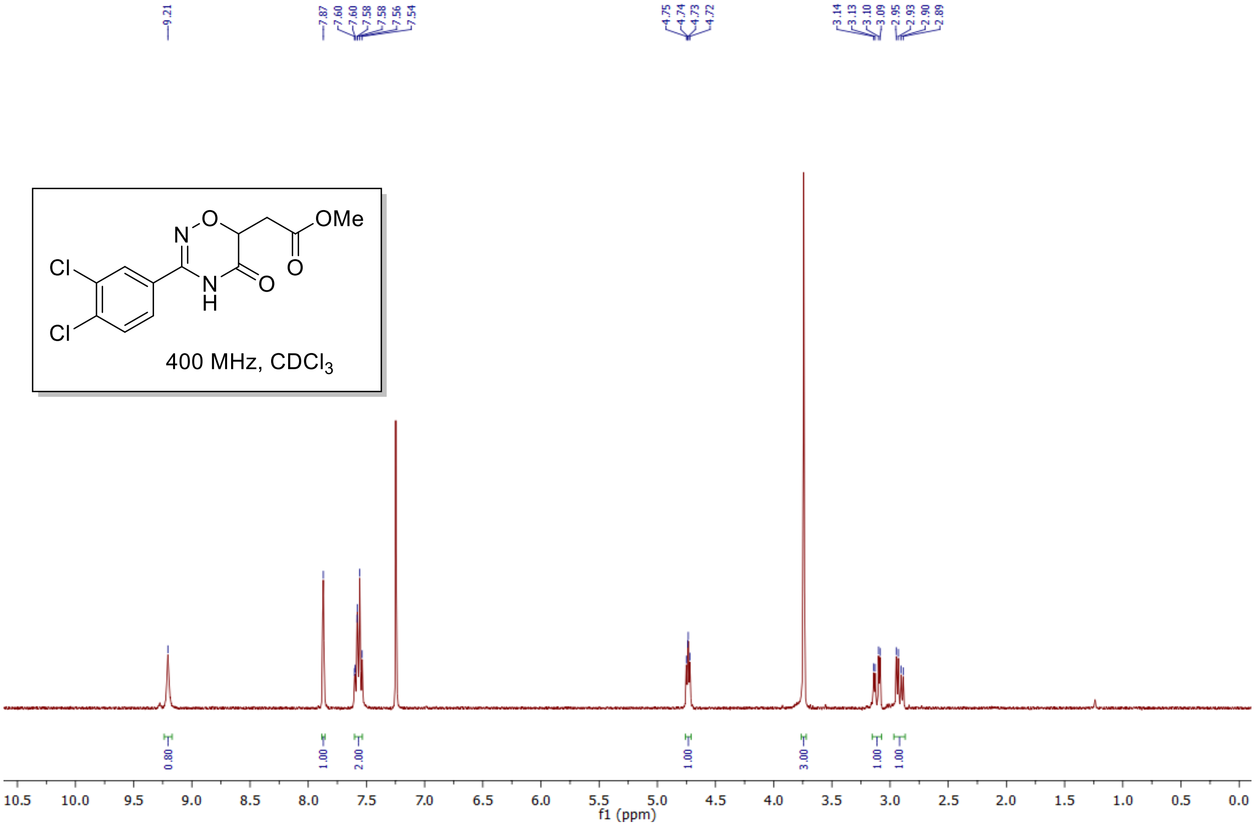

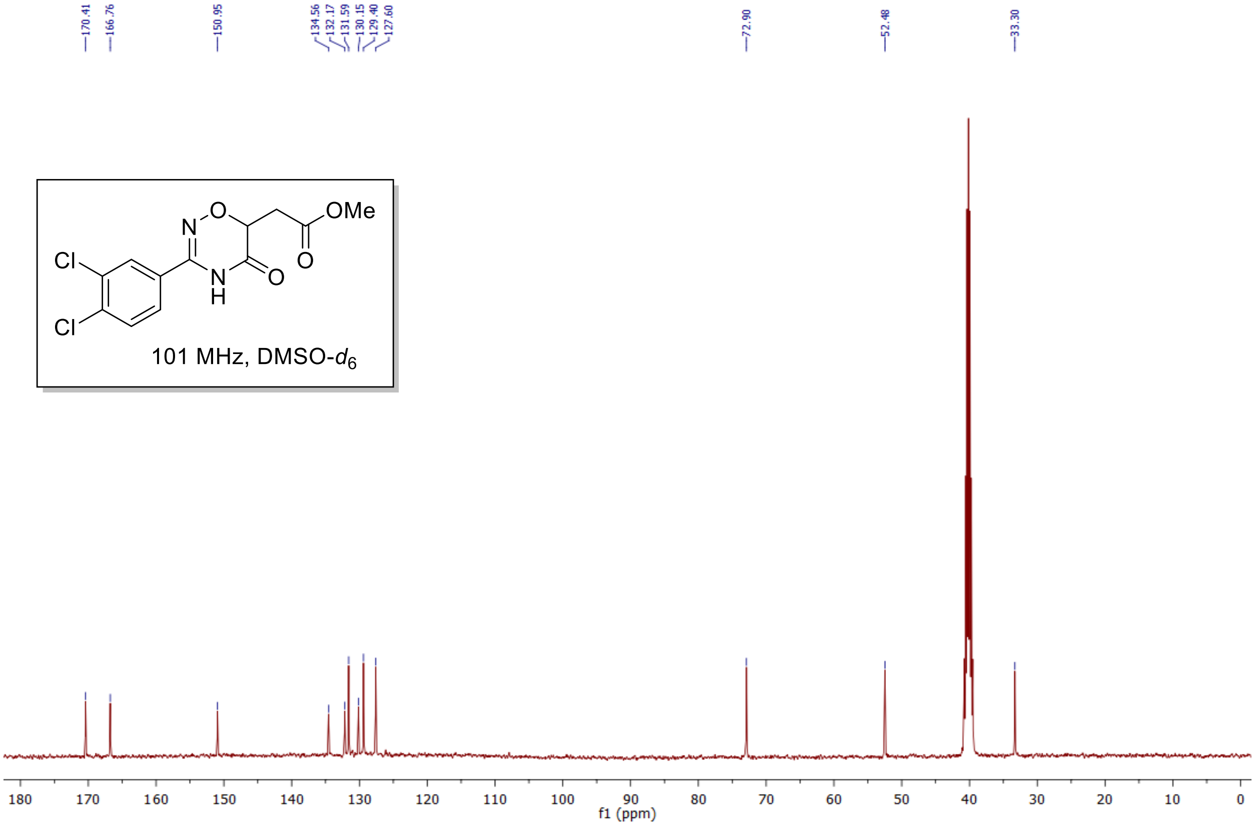

^{1}H and ^{13}C spectra of methyl 2-(3-(3,5-dichlorophenyl)-5-oxo-5,6-dihydro-4*H*-1,2,4-oxadiazin-6-yl)acetate **4m**

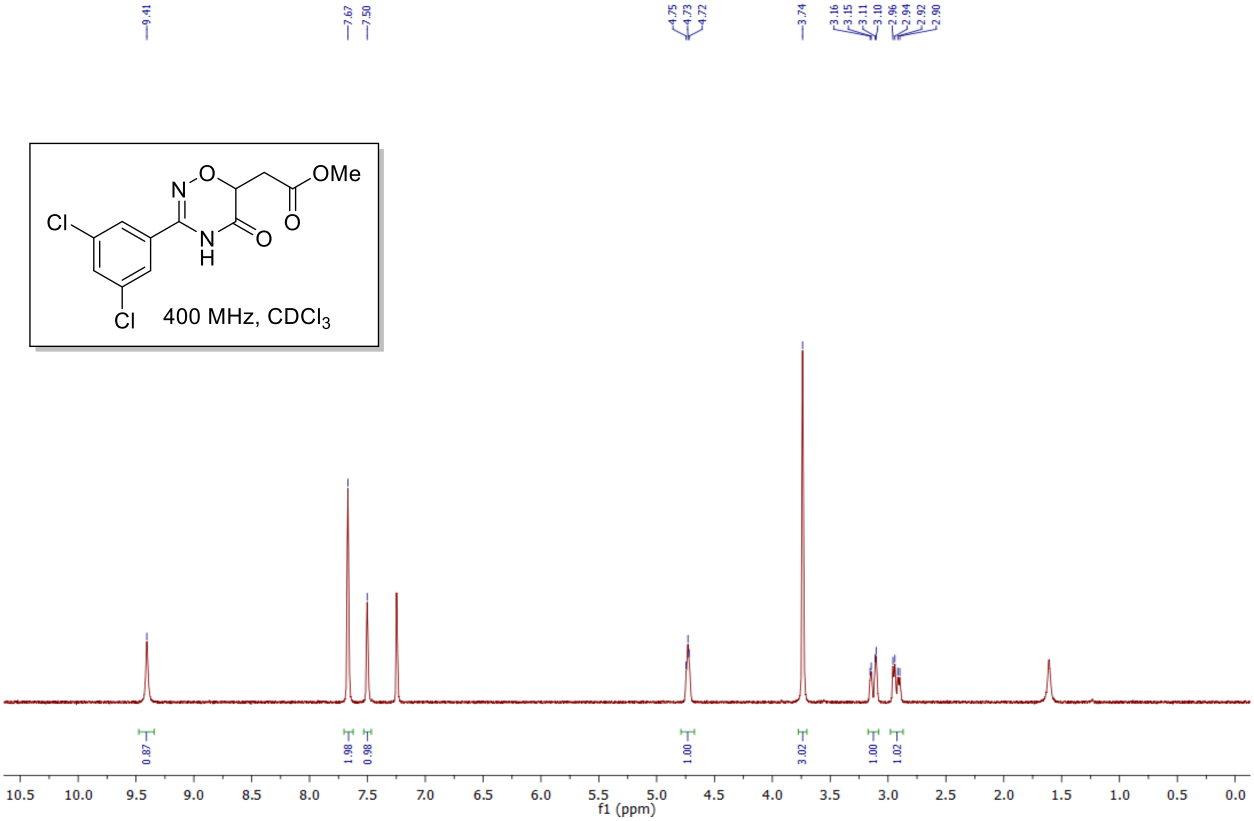

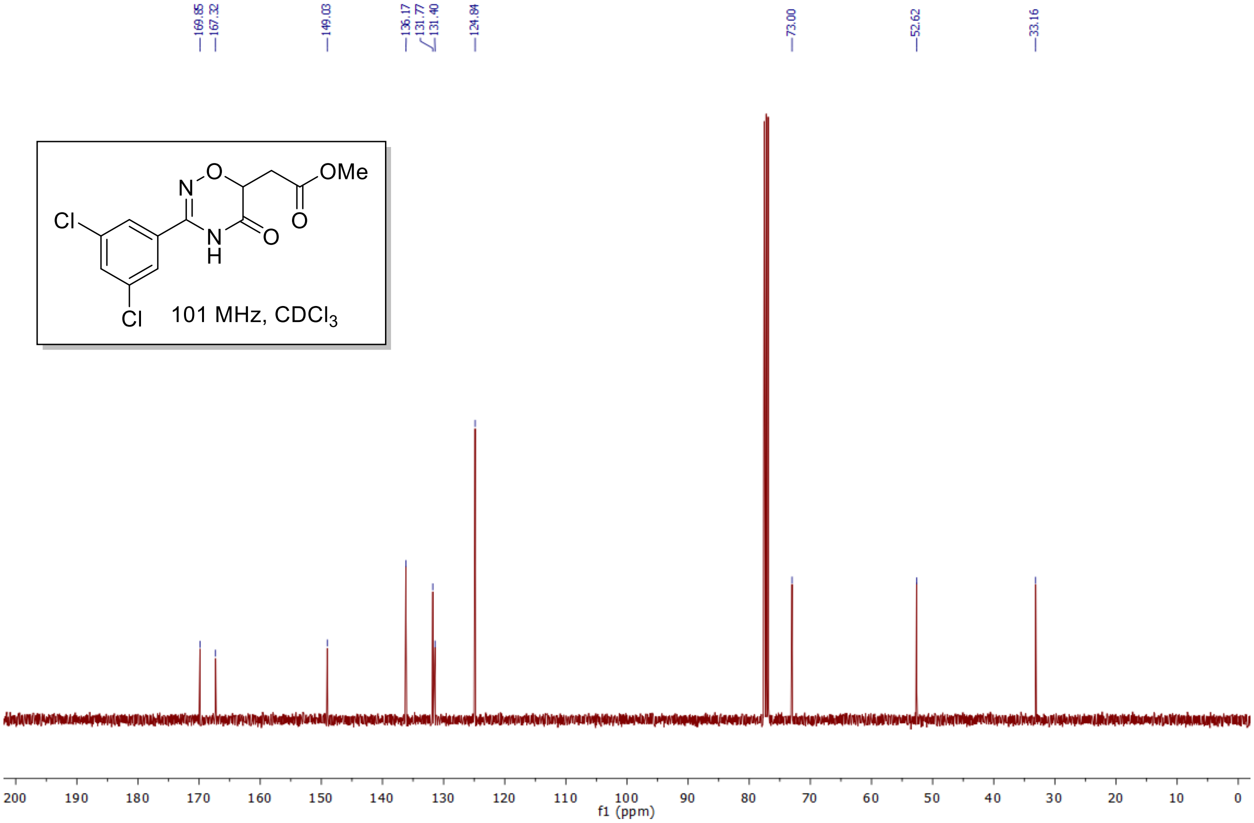

^{1}H and ^{13}C spectra of methyl 2-(3-(5-methylthiophen-2-yl)-5-oxo-5,6-dihydro-4*H*-1,2,4-oxadiazin-6-yl)acetate **4n**

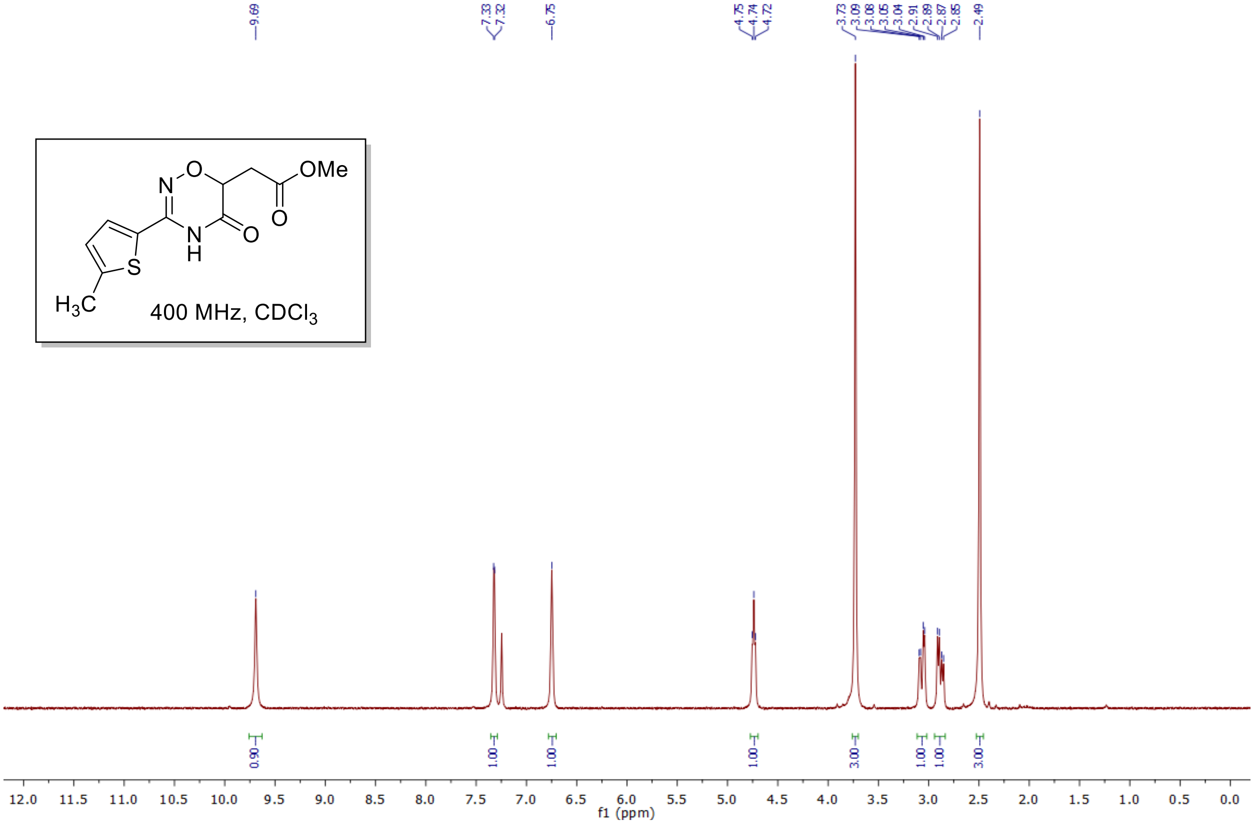

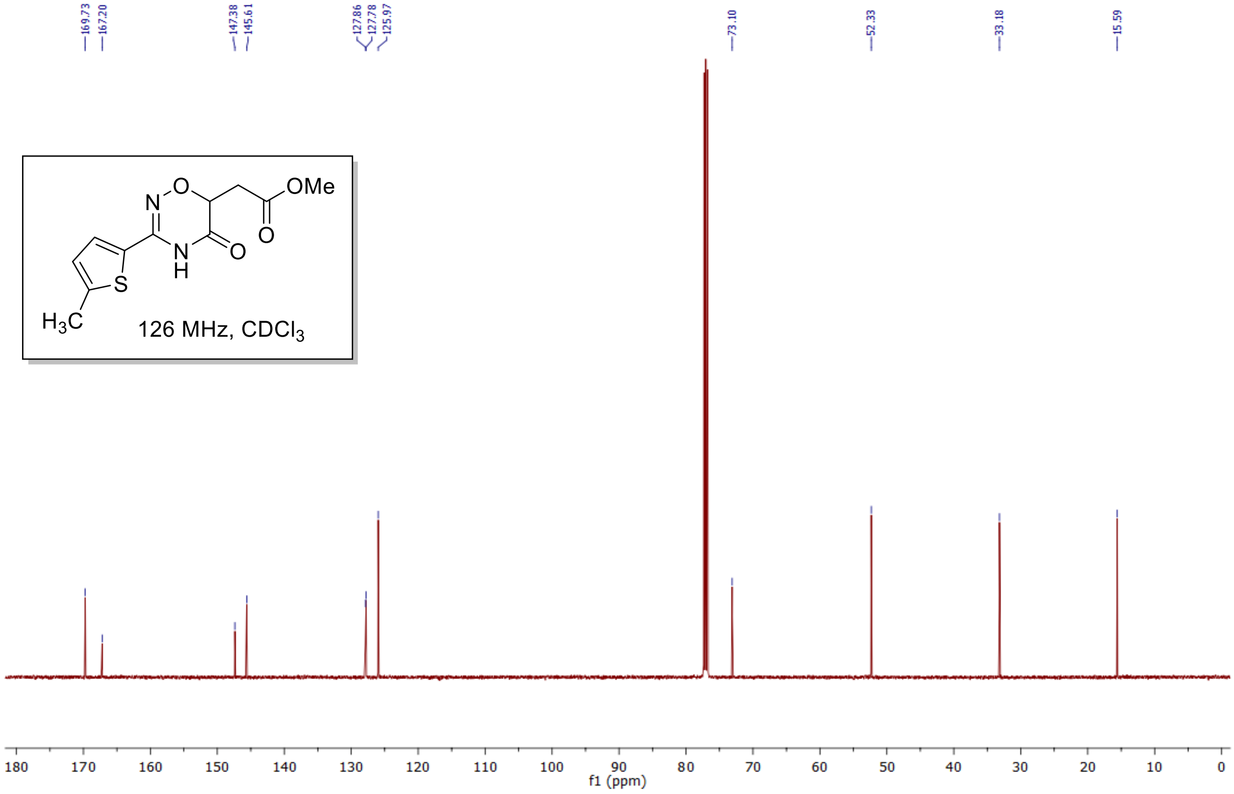

^{1}H and ^{13}C spectra of methyl 2-(5-oxo-3-(pyridin-4-yl)-5,6-dihydro-4*H*-1,2,4-oxadiazin-6-yl)acetate **4o**

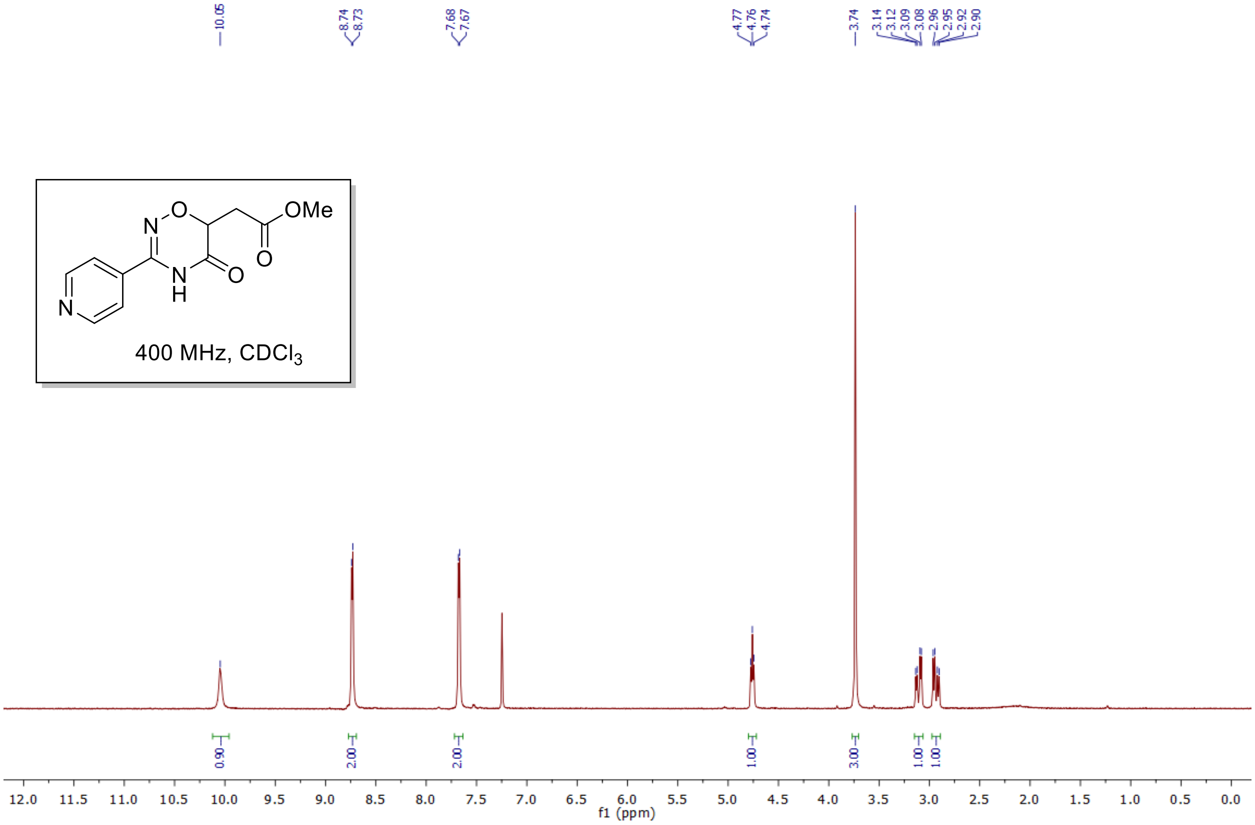

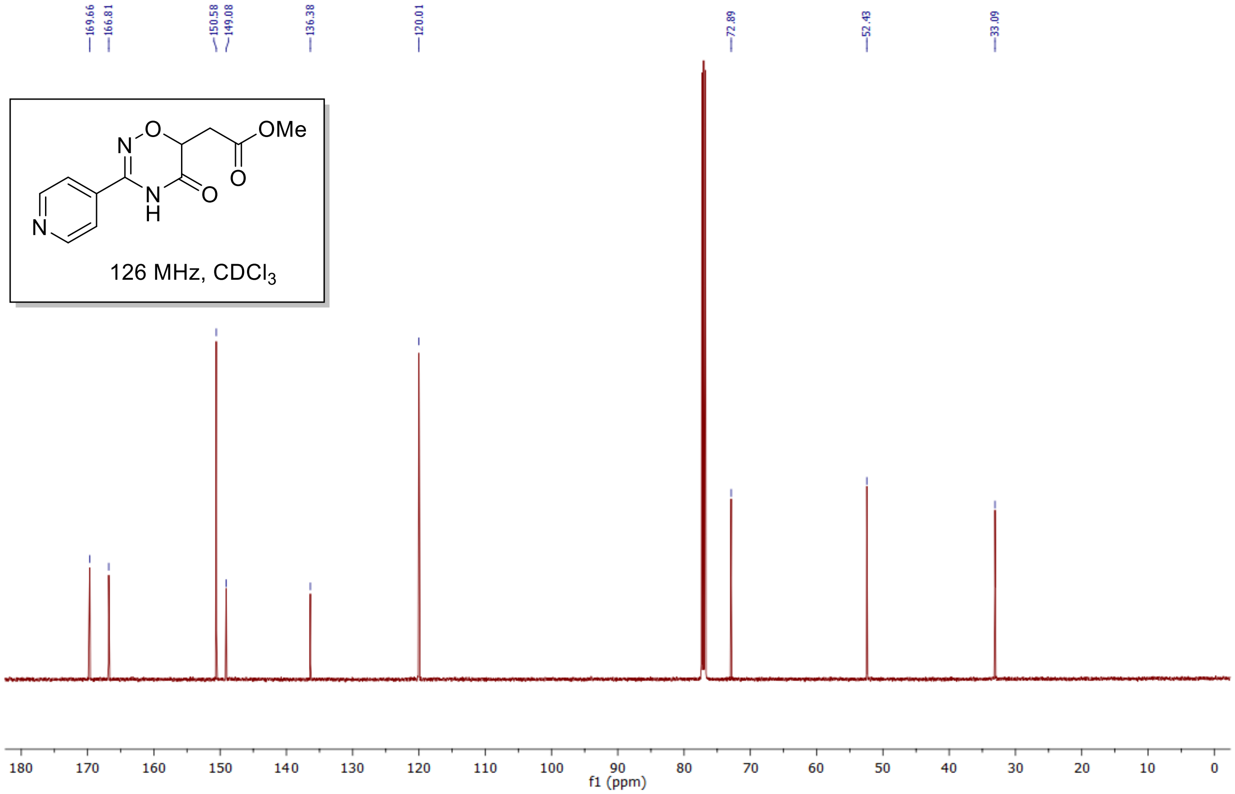

^{1}H and ^{13}C spectra of methyl 2-(5-oxo-3-(pyridin-2-yl)-5,6-dihydro-4*H*-1,2,4-oxadiazin-6-yl)acetate **4p**

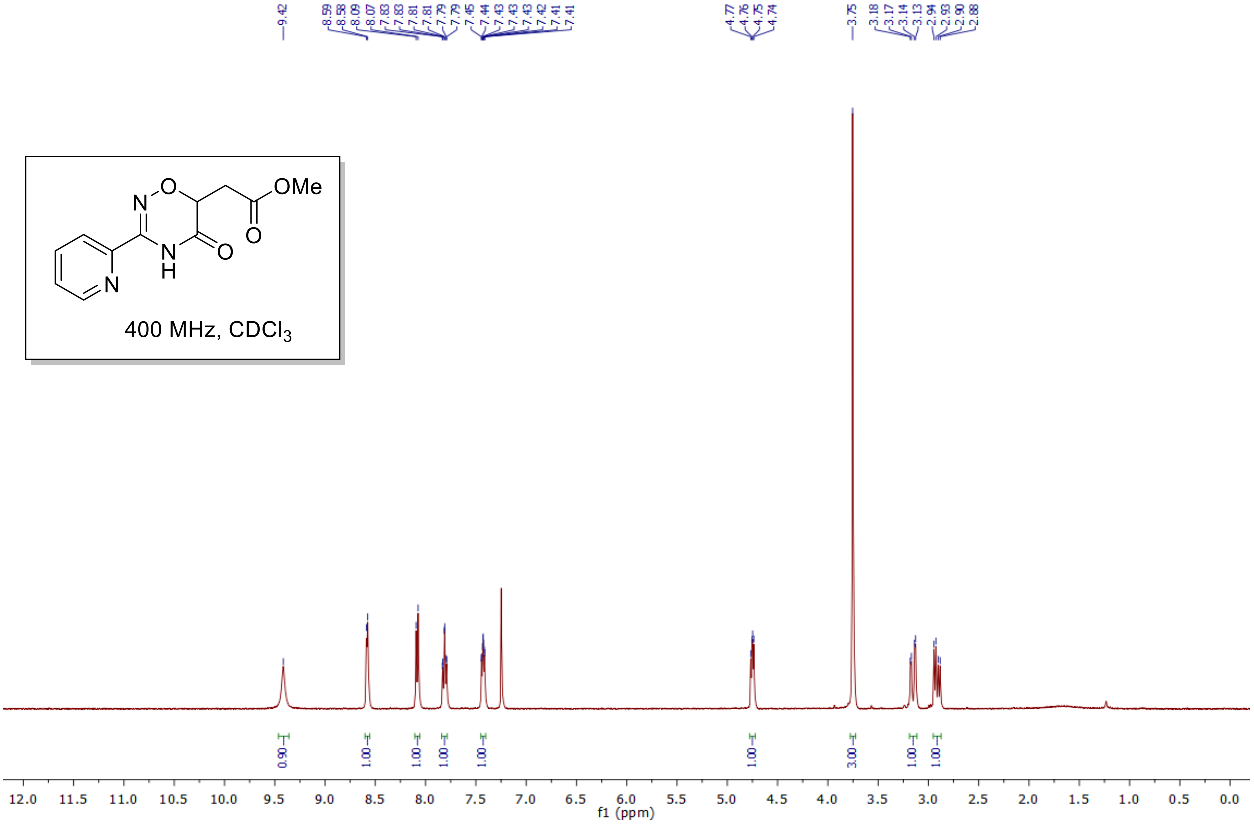

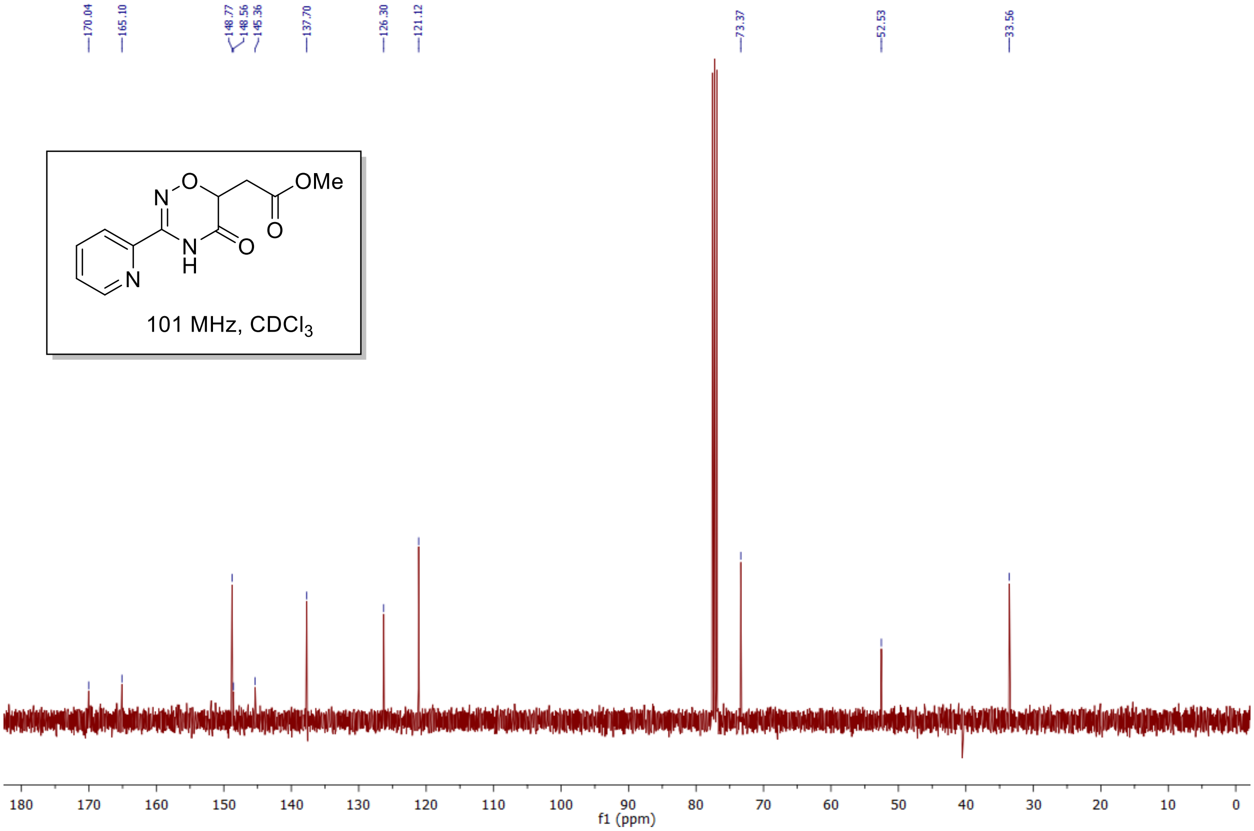

^{1}H and ^{13}C spectra of 3-(4-methylphenyl)-6-((3-(4-methylphenyl)-1,2,4-oxadiazol-5-yl)methyl)-4*H*-1,2,4-oxadiazin-5(6*H*)-one **5a**

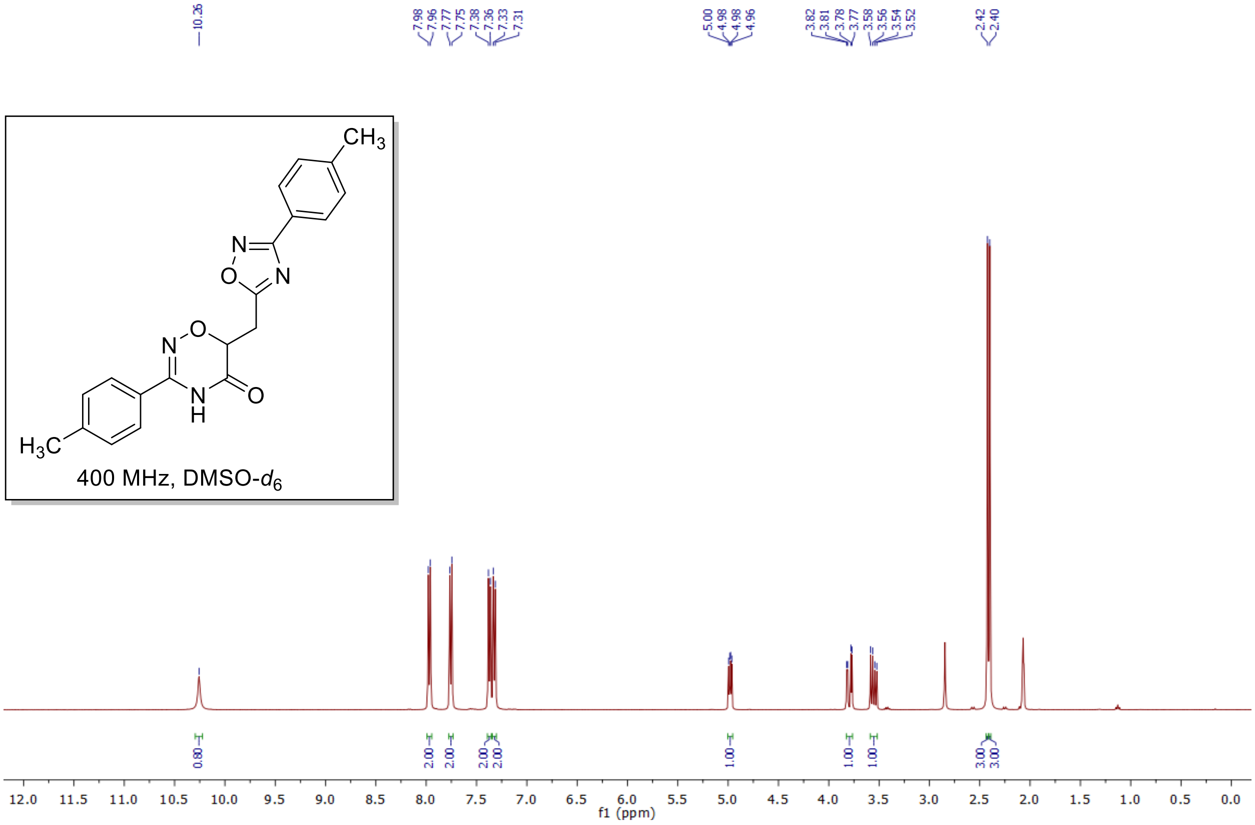

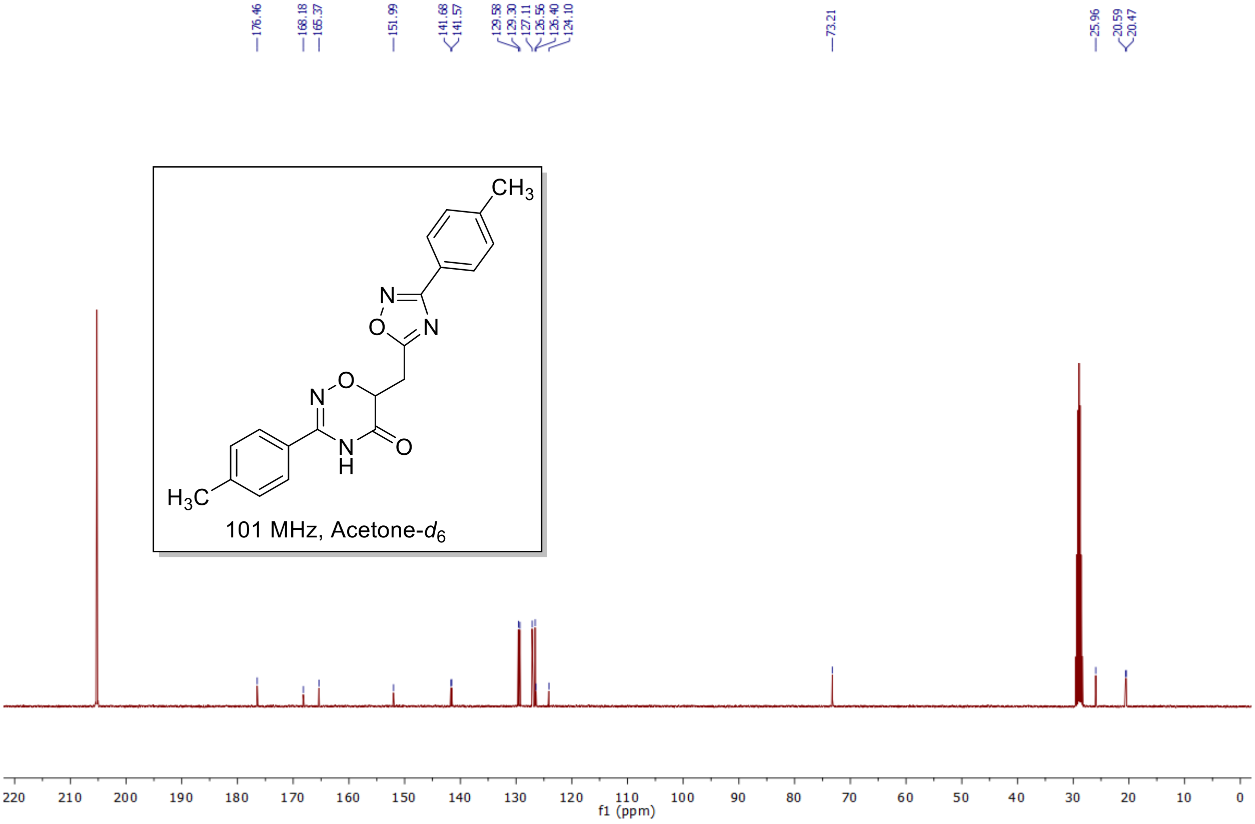

^{1}H and ^{13}C spectra of 3-phenyl-6-((3-phenyl-1,2,4-oxadiazol-5-yl)methyl)-4*H*-1,2,4-oxadiazin-5(6*H*)-one **5b**

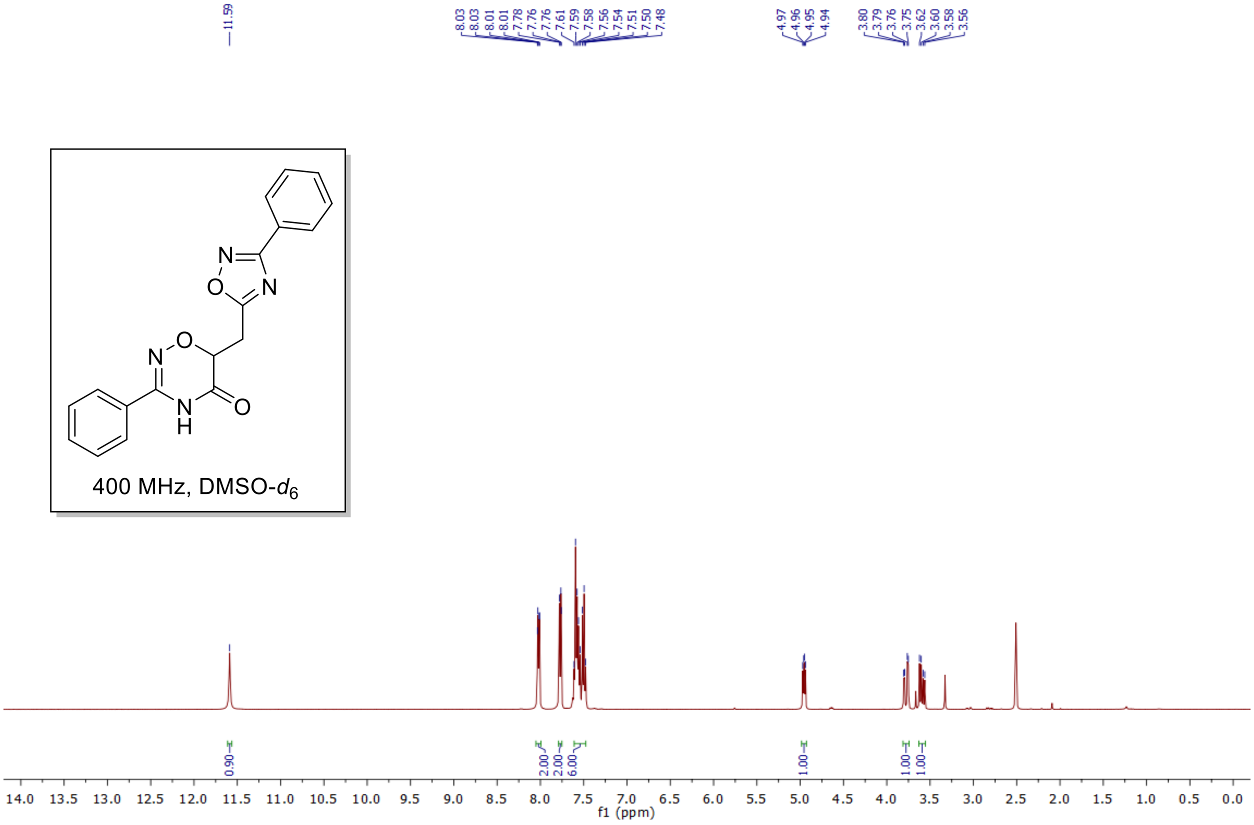

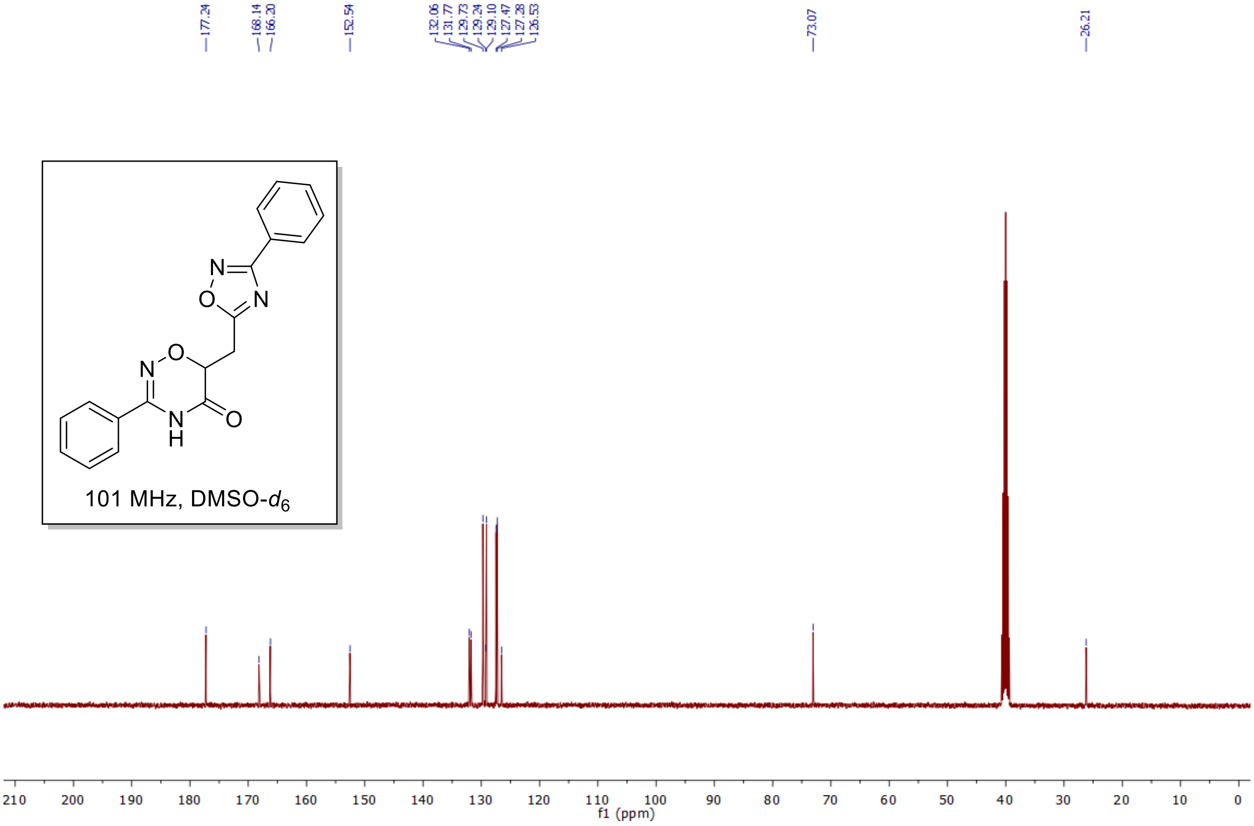

^{1}H and ^{13}C spectra of 3-(4-methoxyphenyl)-6-((3-(4-methoxyphenyl)-1,2,4-oxadiazol-5-yl)methyl)-4*H*-1,2,4-oxadiazin-5(6*H*)-one **5c**

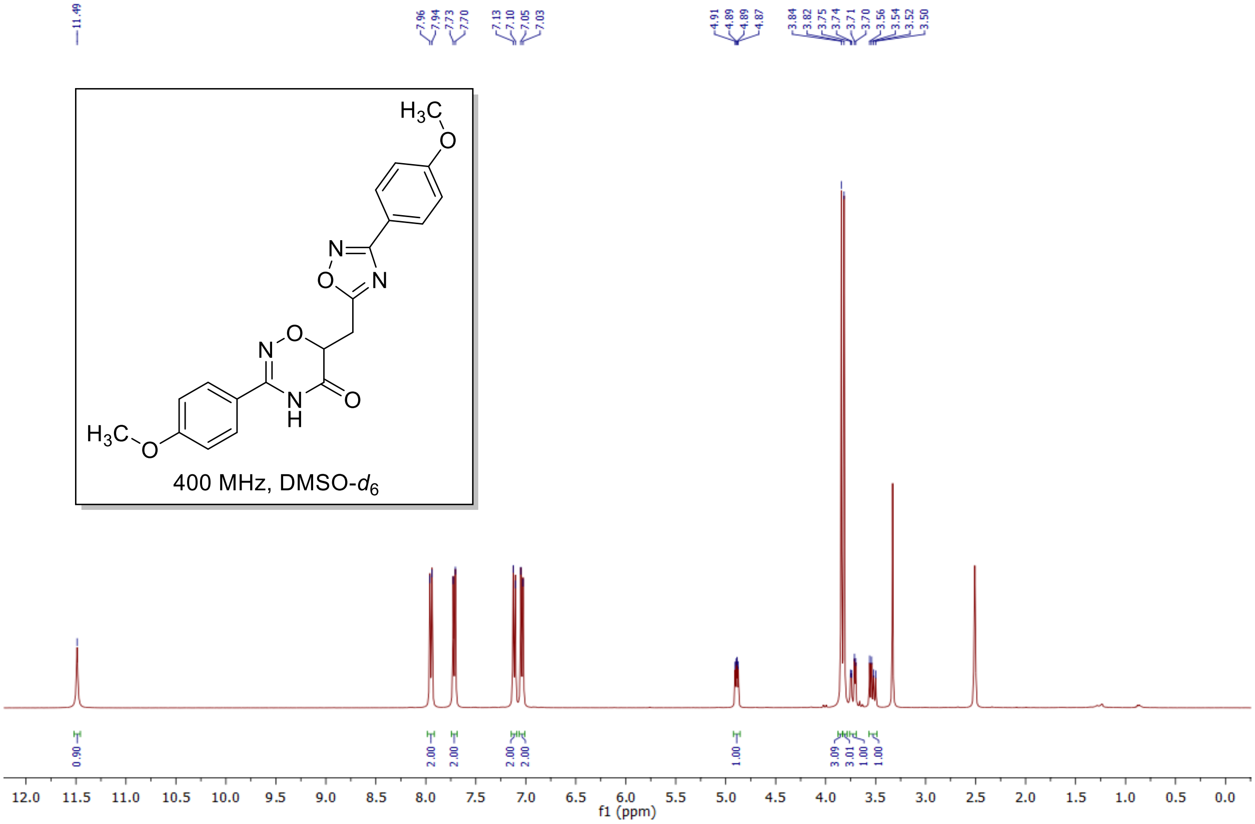

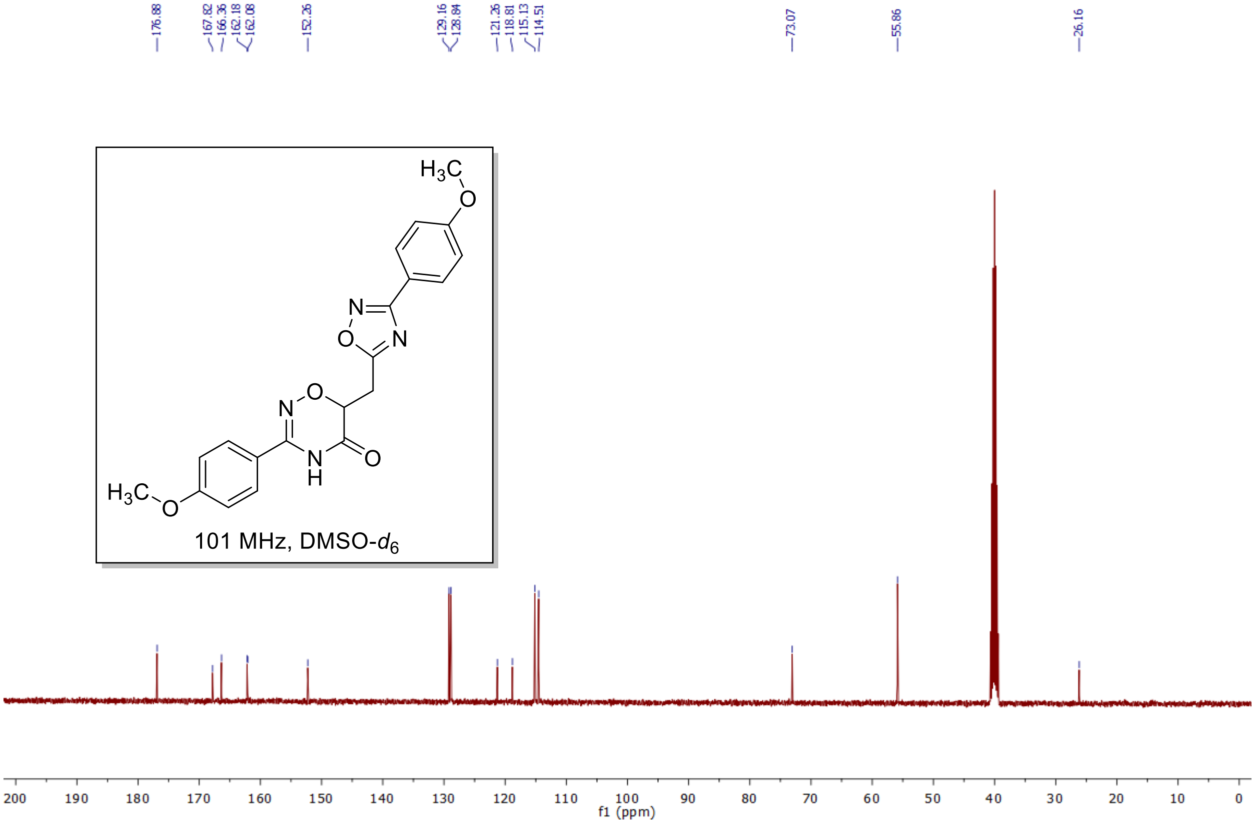

^{1}H and ^{13}C spectra of 3-(4-nitrophenyl)-6-((3-(4-nitrophenyl)-1,2,4-oxadiazol-5-yl)methyl)-4*H*-1,2,4-oxadiazin-5(6*H*)-one **5d**

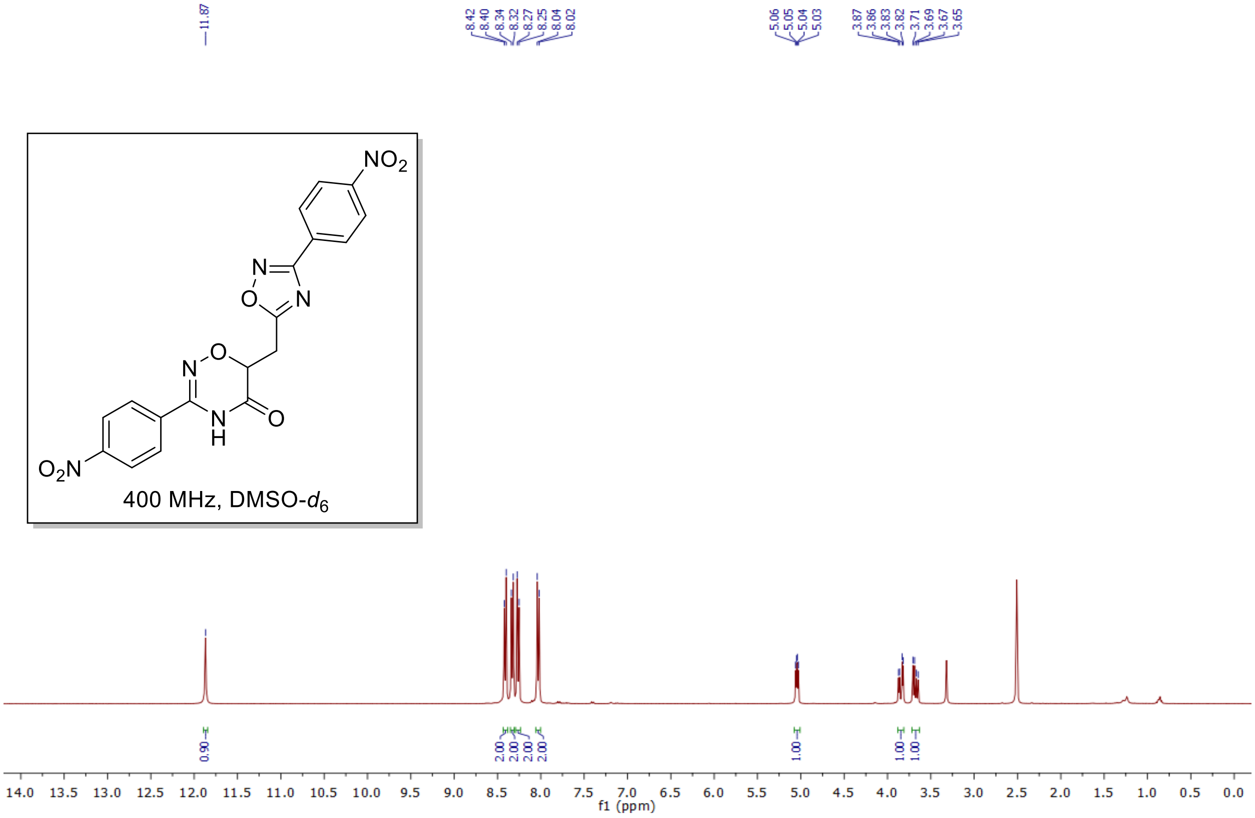

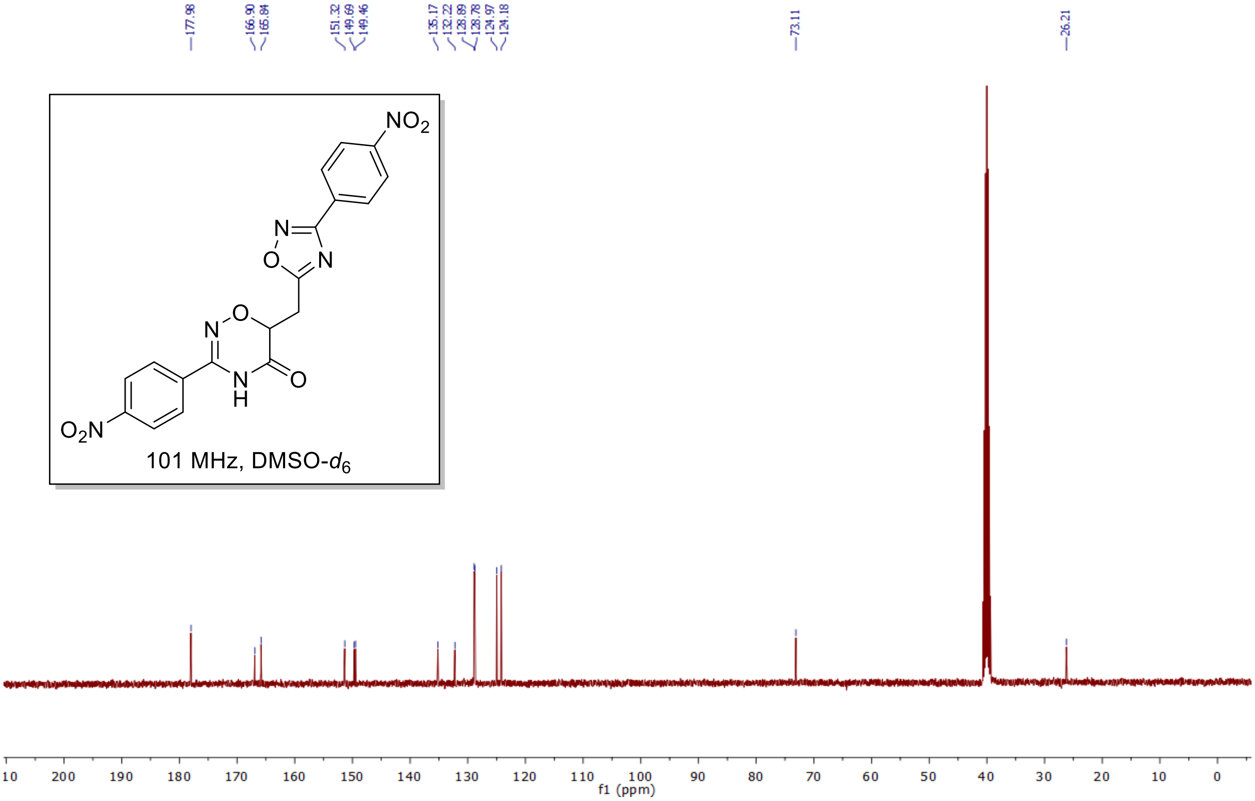

^{1}H, ^{13}C and ^{19}F spectra of 3-(4-(trifluoromethyl)phenyl)-6-((3-(4-(trifluoromethyl)phenyl)-1,2,4-oxadiazol-5-yl)methyl)-4*H*-1,2,4-oxadiazin-5(6*H*)-one **5e**

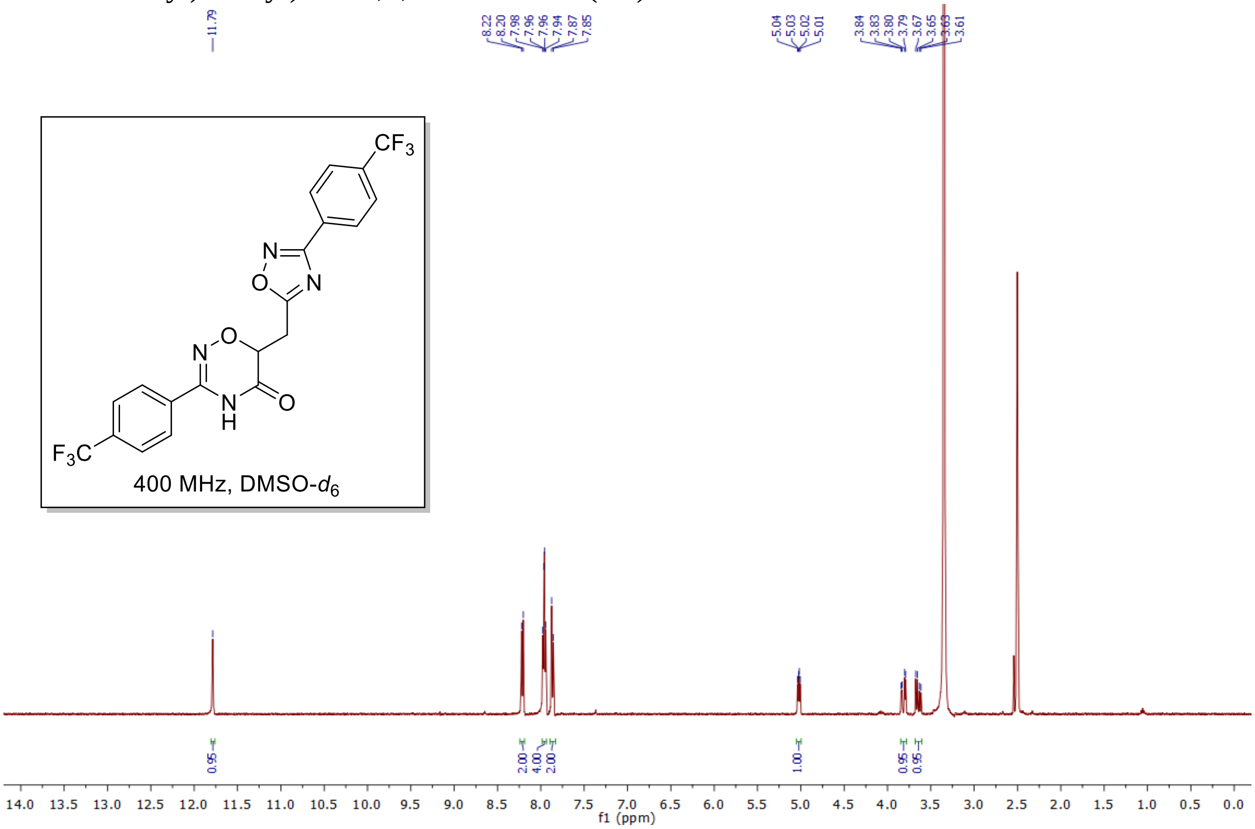

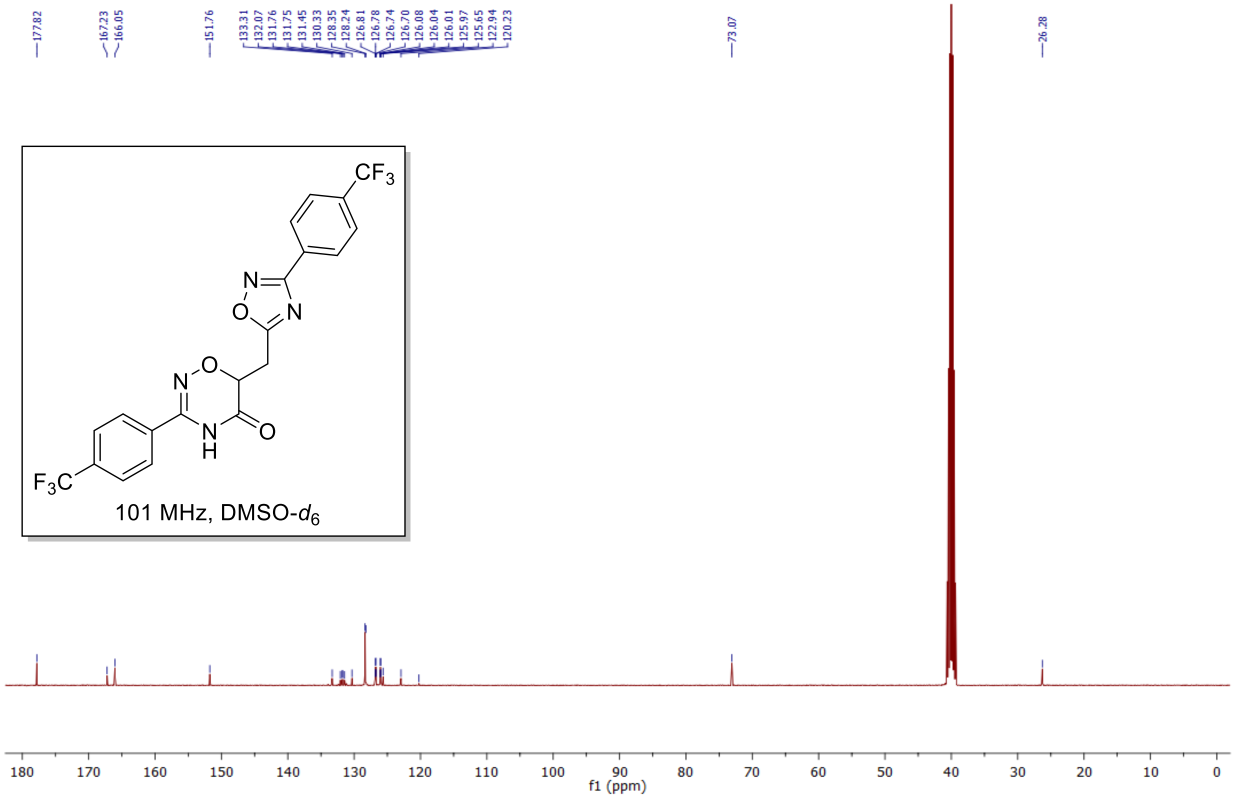

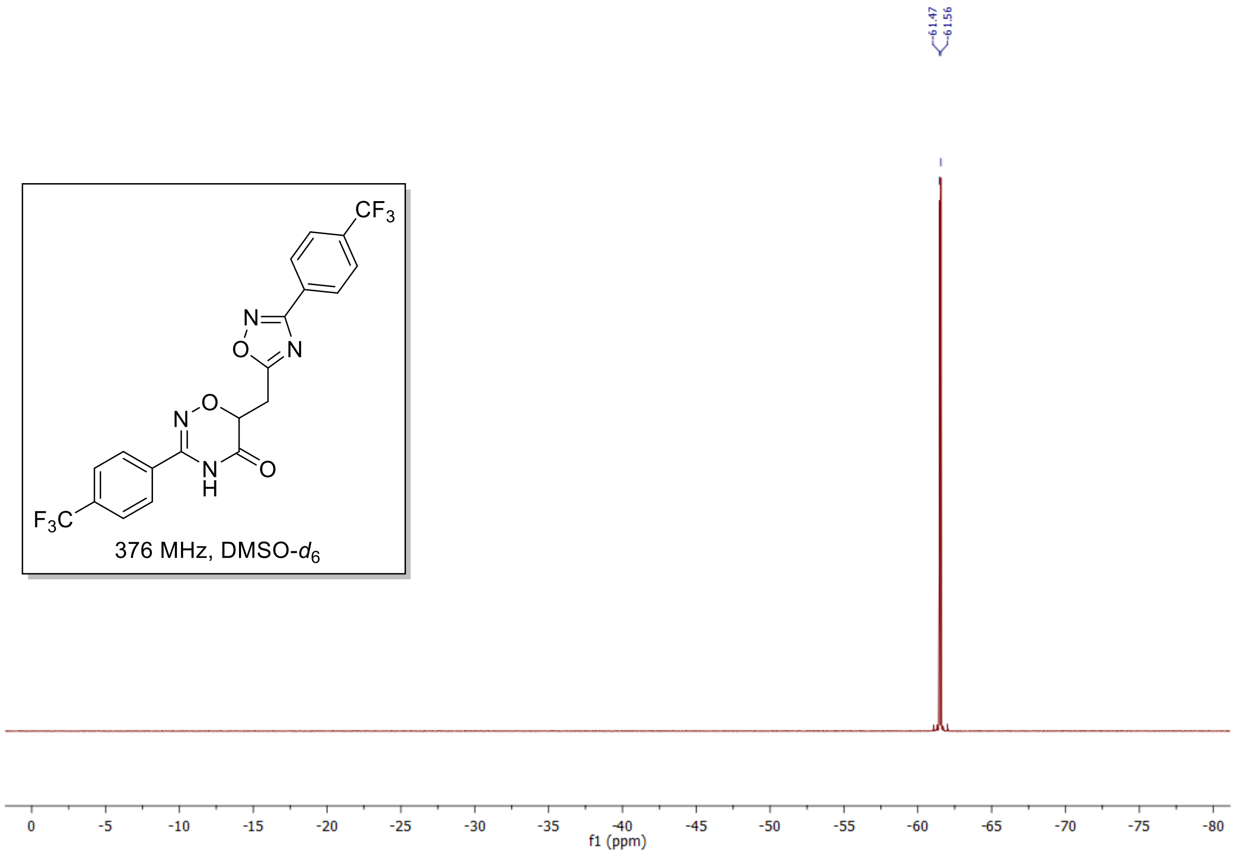
-61.47
-61.56
CF3
N
O
N
N
O
N
H
O
F3C
376 MHz, DMSO-d6
0
-5
-10
-15
-20
-25
-30
-35
-40
-45
-50
-55
-60
-65
-70
-75
-80
f1 (ppm)

^{1}H, ^{13}C and ^{19}F spectra of 3-(4-fluorophenyl)-6-((3-(4-fluorophenyl)-1,2,4-oxadiazol-5-yl)methyl)-4*H*-1,2,4-oxadiazin-5(6*H*)-one **5f**

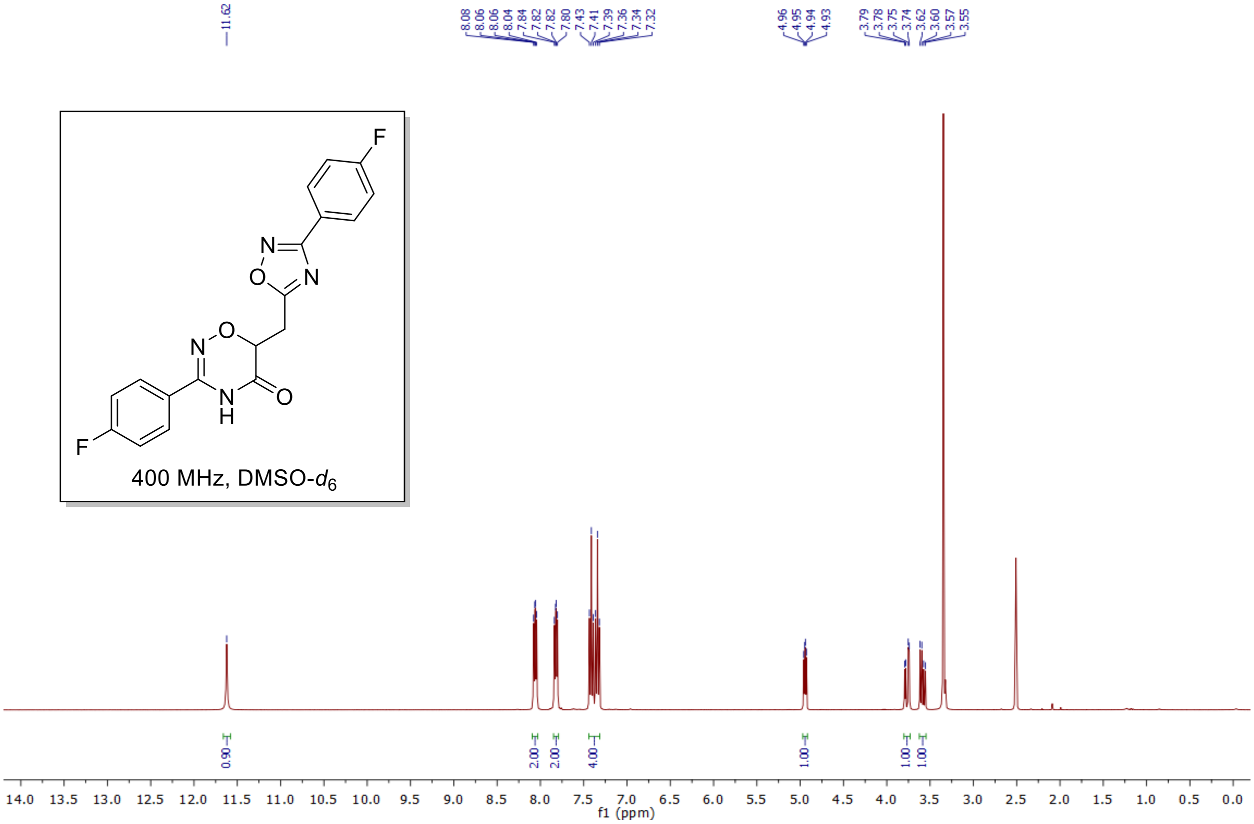

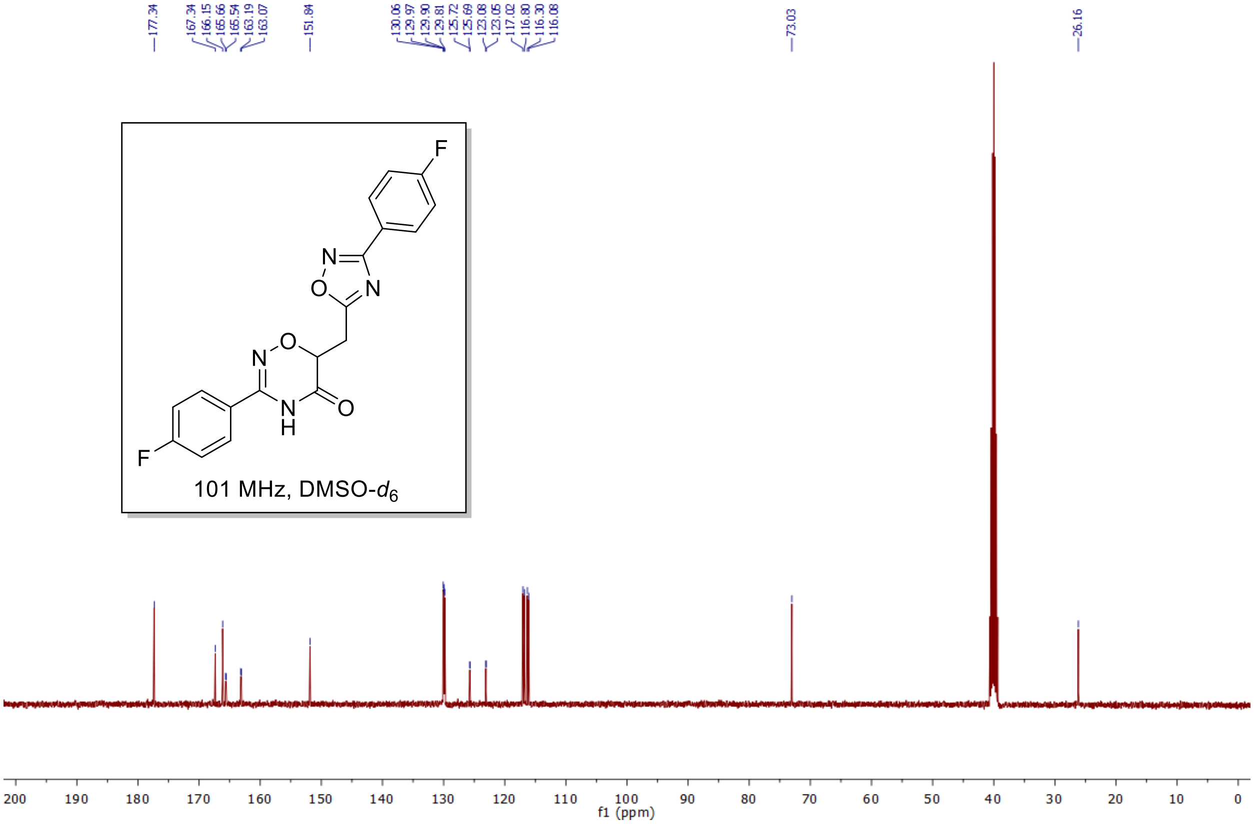

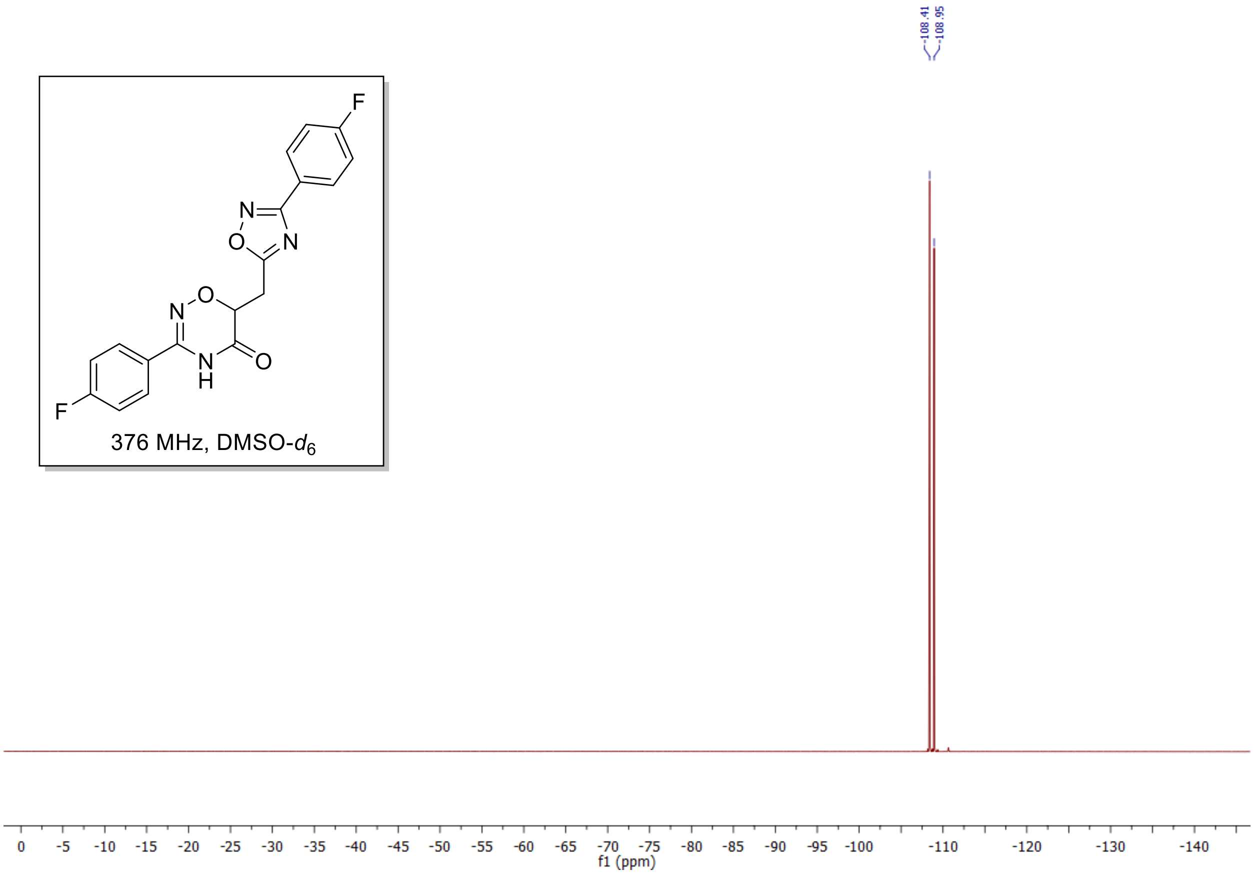

F
N
O
N
O
N
N
H
O
F
376 MHz, DMSO-d6
-108.41
-108.95
0
-5
-10
-15
-20
-25
-30
-35
-40
-45
-50
-55
-60
-65
-70
-75
-80
-85
-90
-95
-100
-110
-120
-130
-140
f1 (ppm)

^{1}H and ^{13}C spectra of 3-(4-chlorophenyl)-6-((3-(4-chlorophenyl)-1,2,4-oxadiazol-5-yl)methyl)-4*H*-1,2,4-oxadiazin-5(6*H*)-one **5g**

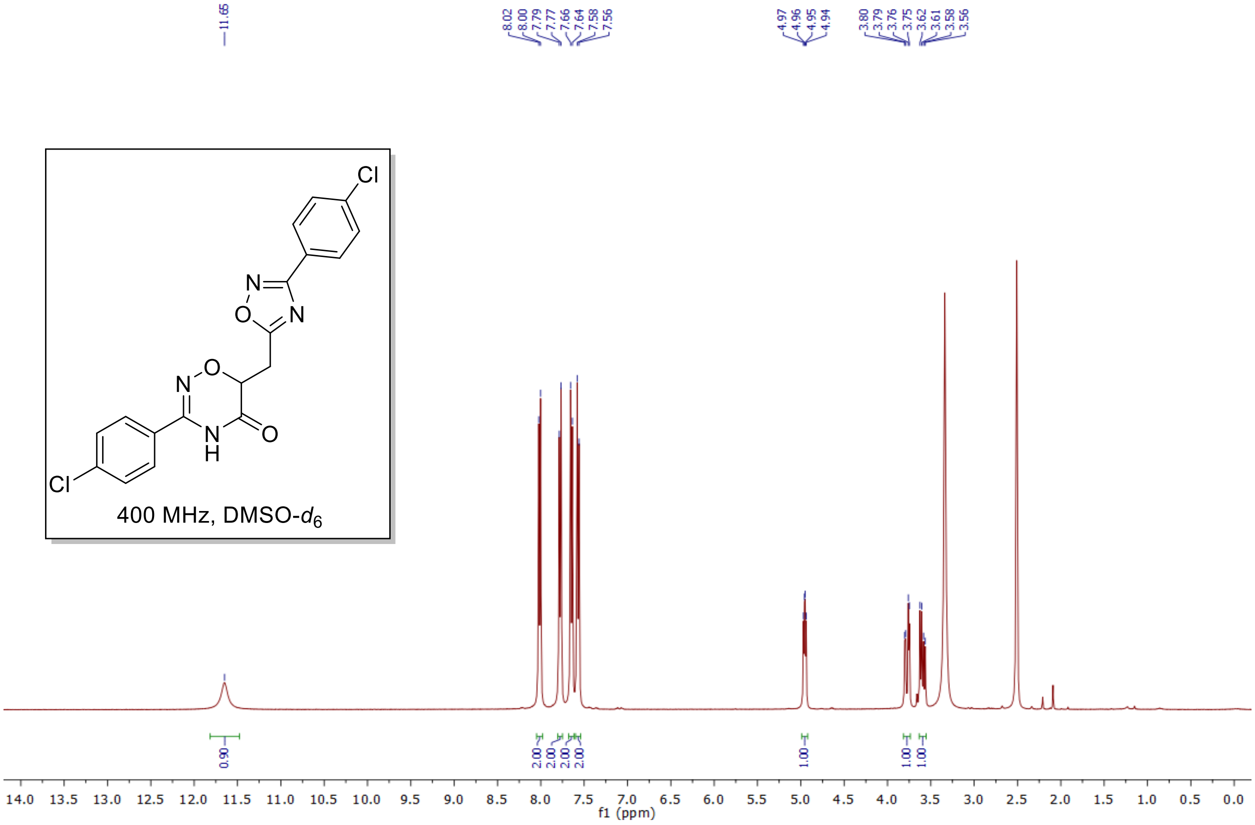

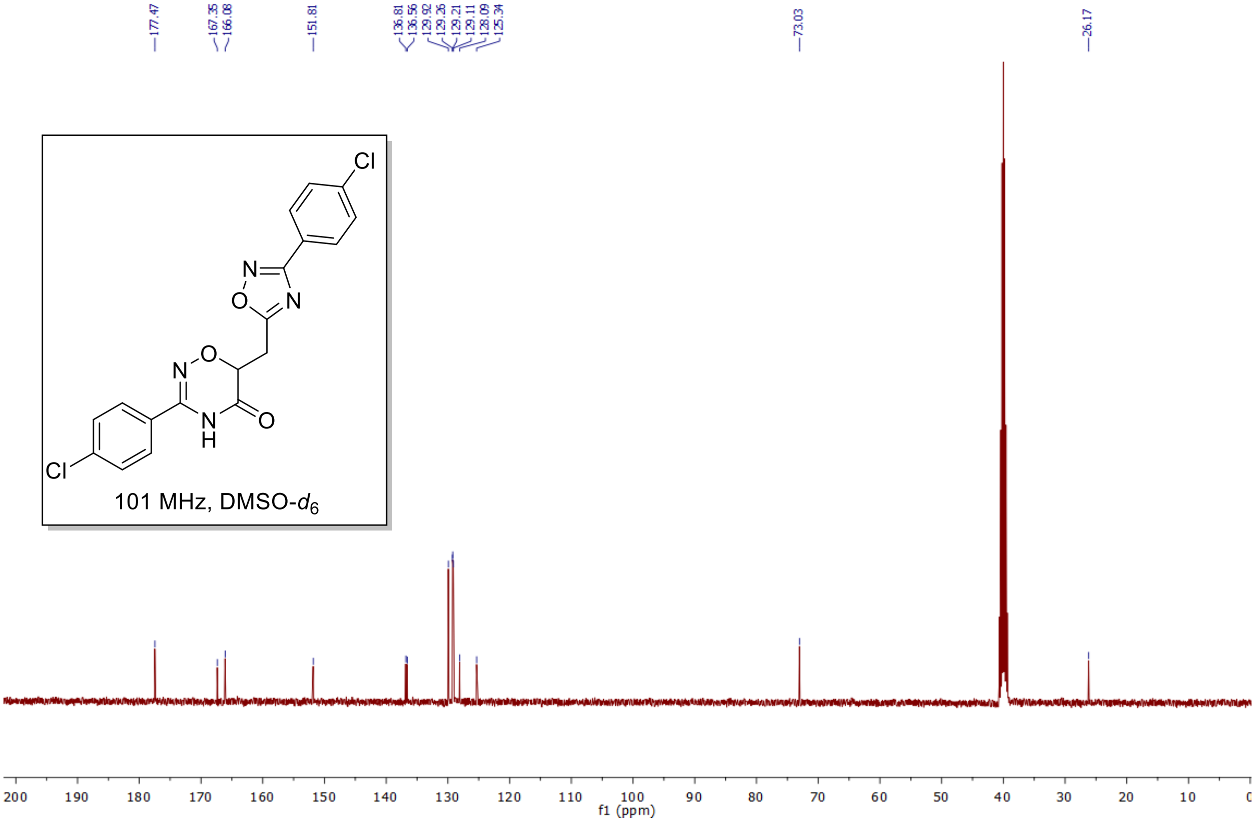

^{1}H and ^{13}C spectra of 3-(4-aminophenyl)-6-((3-(4-aminophenyl)-1,2,4-oxadiazol-5-yl)methyl)-4H-1,2,4-oxadiazin-5(6*H*)-one **5h**

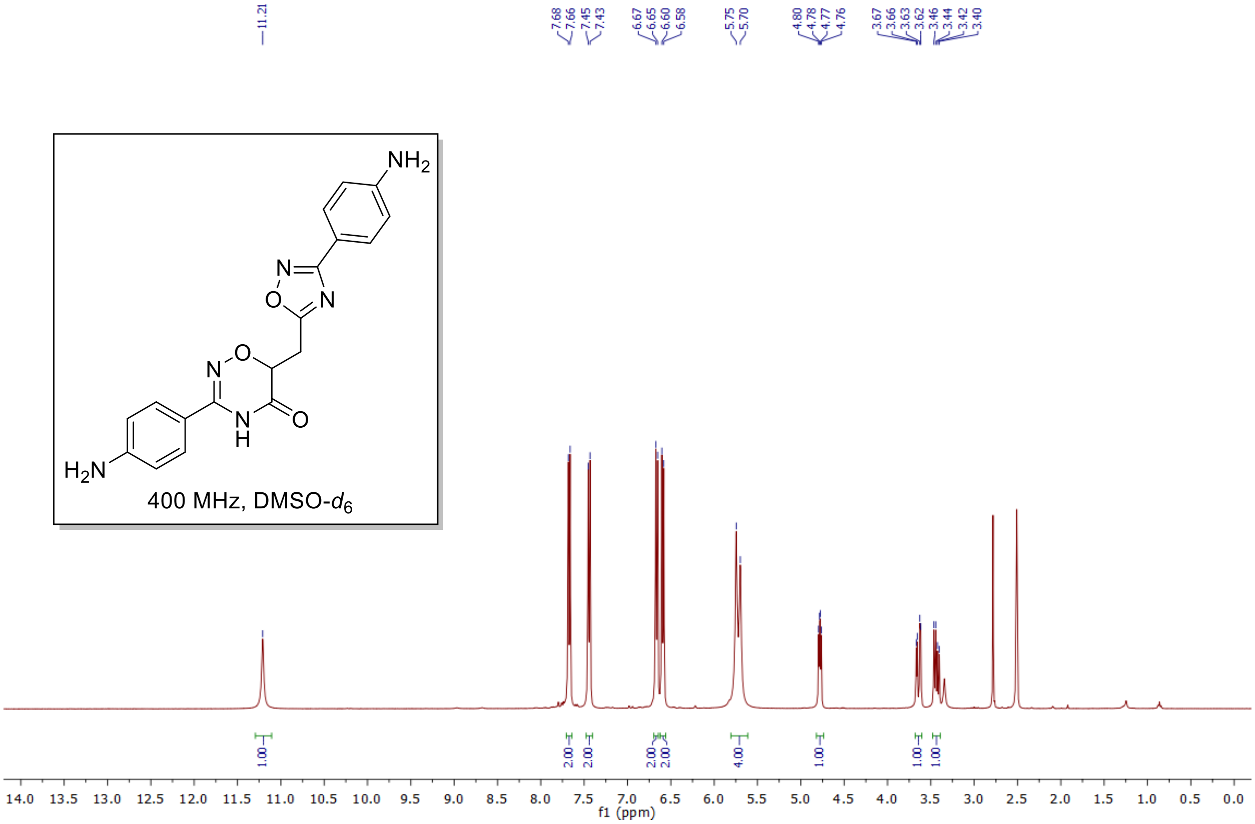

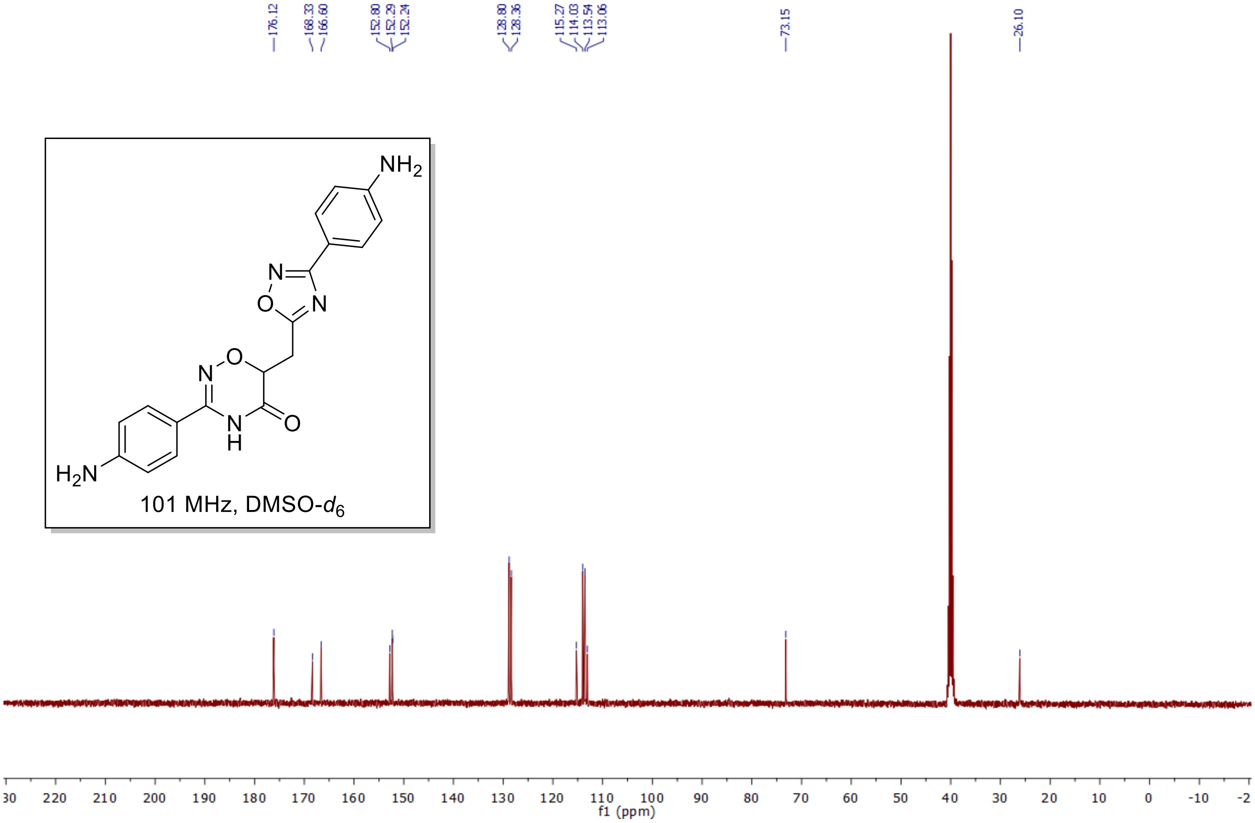

^1H and ^{13}C spectra of 3-(2-chlorophenyl)-6-((3-(2-chlorophenyl)-1,2,4-oxadiazol-5-yl)methyl)-4*H*-1,2,4-oxadiazin-5(6*H*)-one **5i**

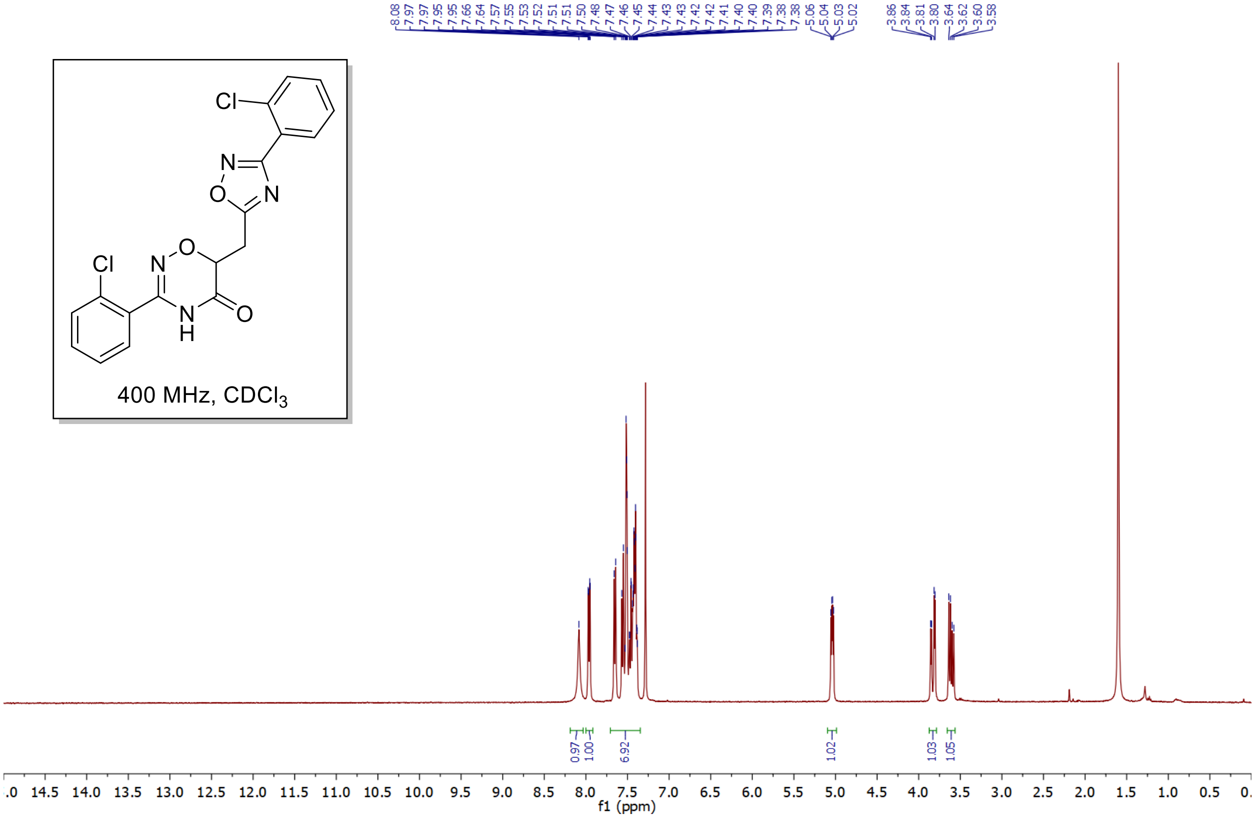

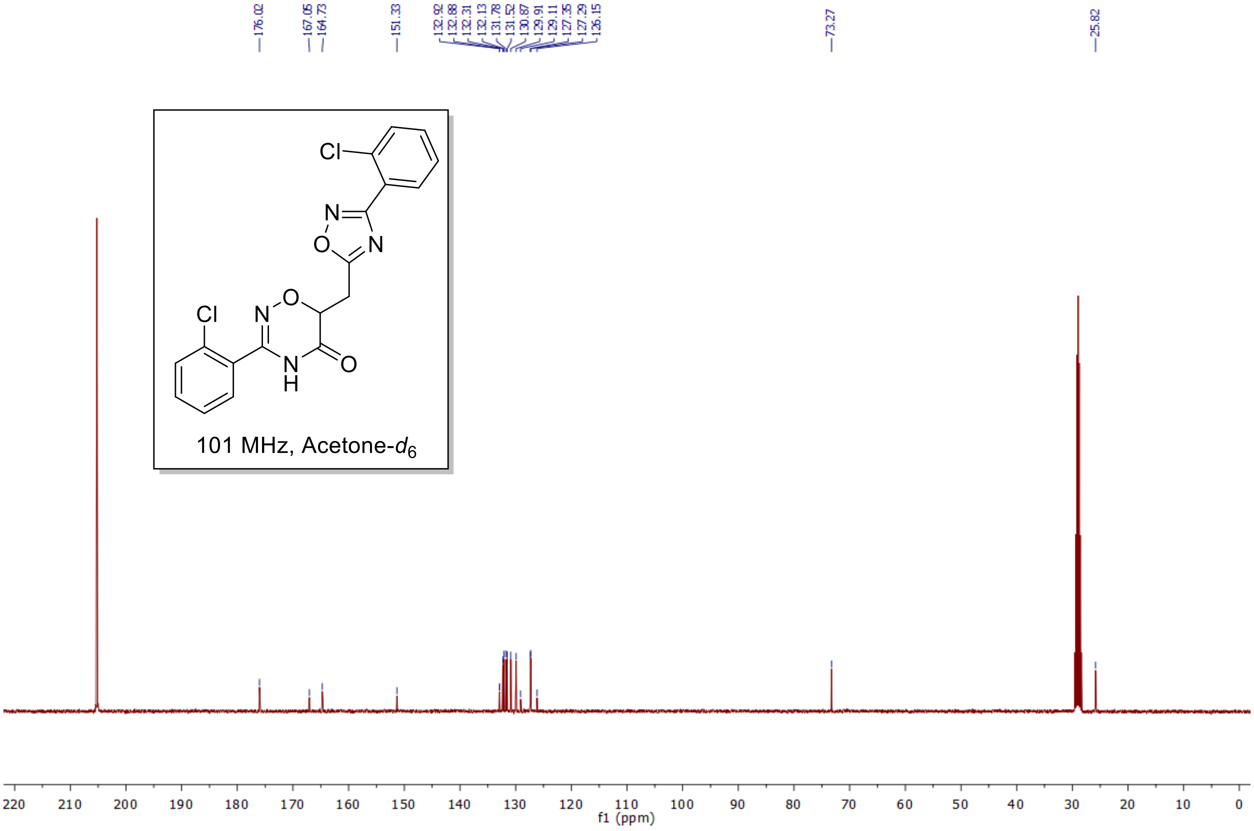

^{1}H and ^{13}C spectra of 3-(5-methylthiophen-2-yl)-6-((3-(5-methylthiophen-2-yl)-1,2,4-oxadiazol-5-yl)methyl)-4*H*-1,2,4-oxadiazin-5(6*H*)-one **5j**

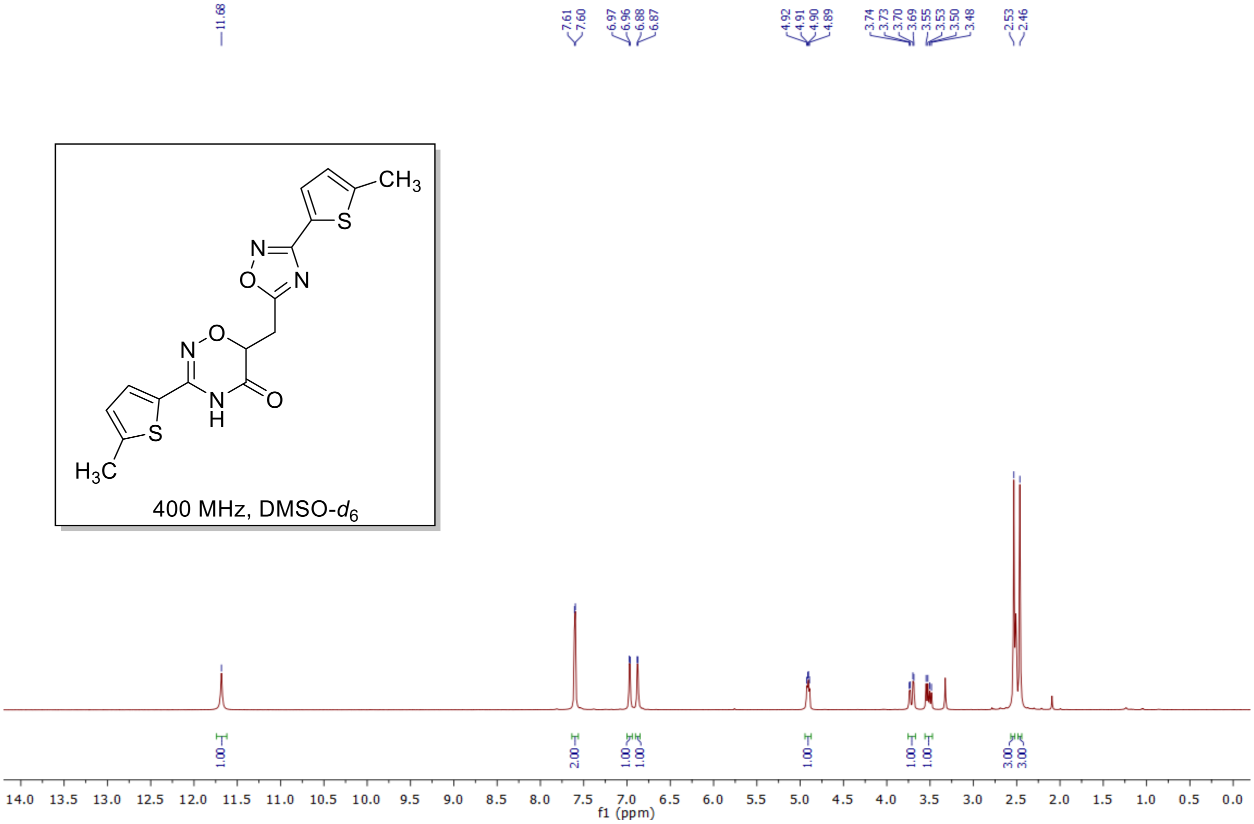

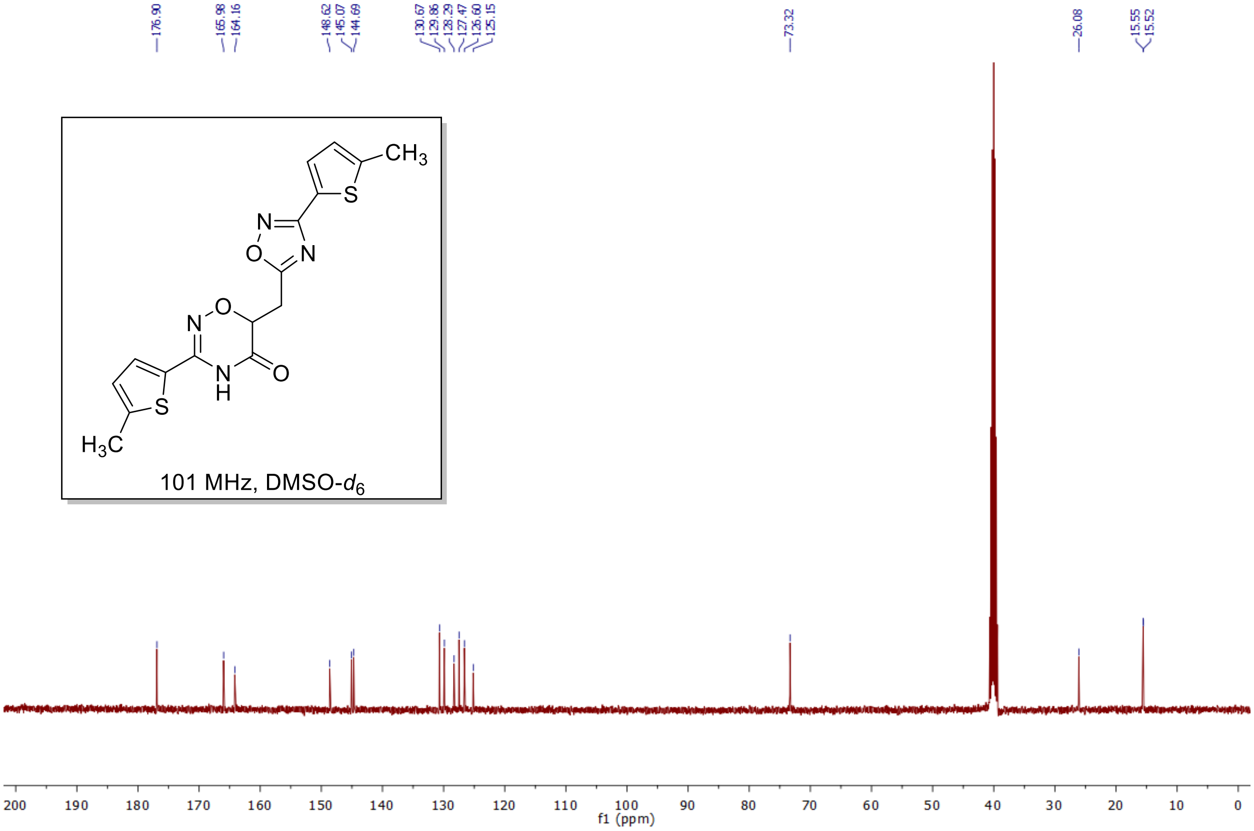

^{1}H and ^{13}C spectra of 3-(pyridin-4-yl)-6-((3-(pyridin-4-yl)-1,2,4-oxadiazol-5-yl)methyl)-4*H*-1,2,4-oxadiazin-5(6*H*)-one **5k**

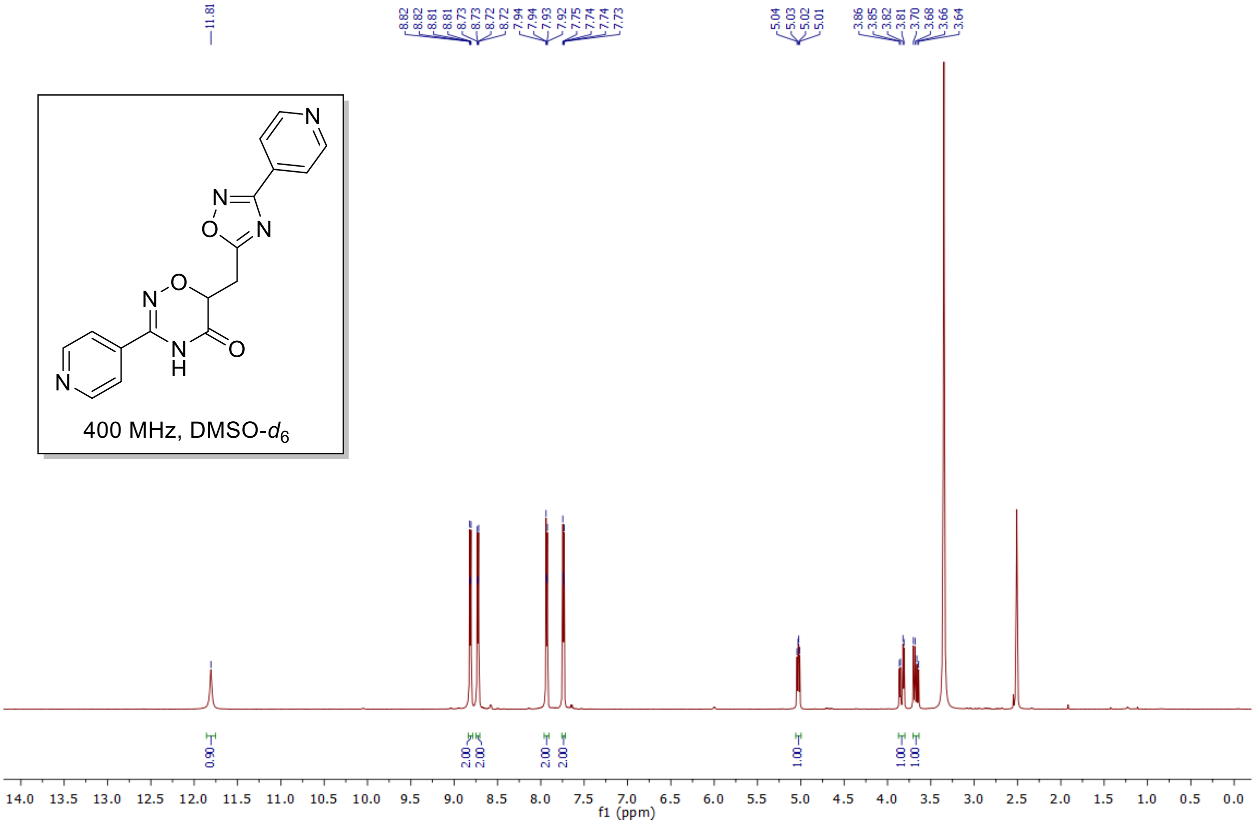

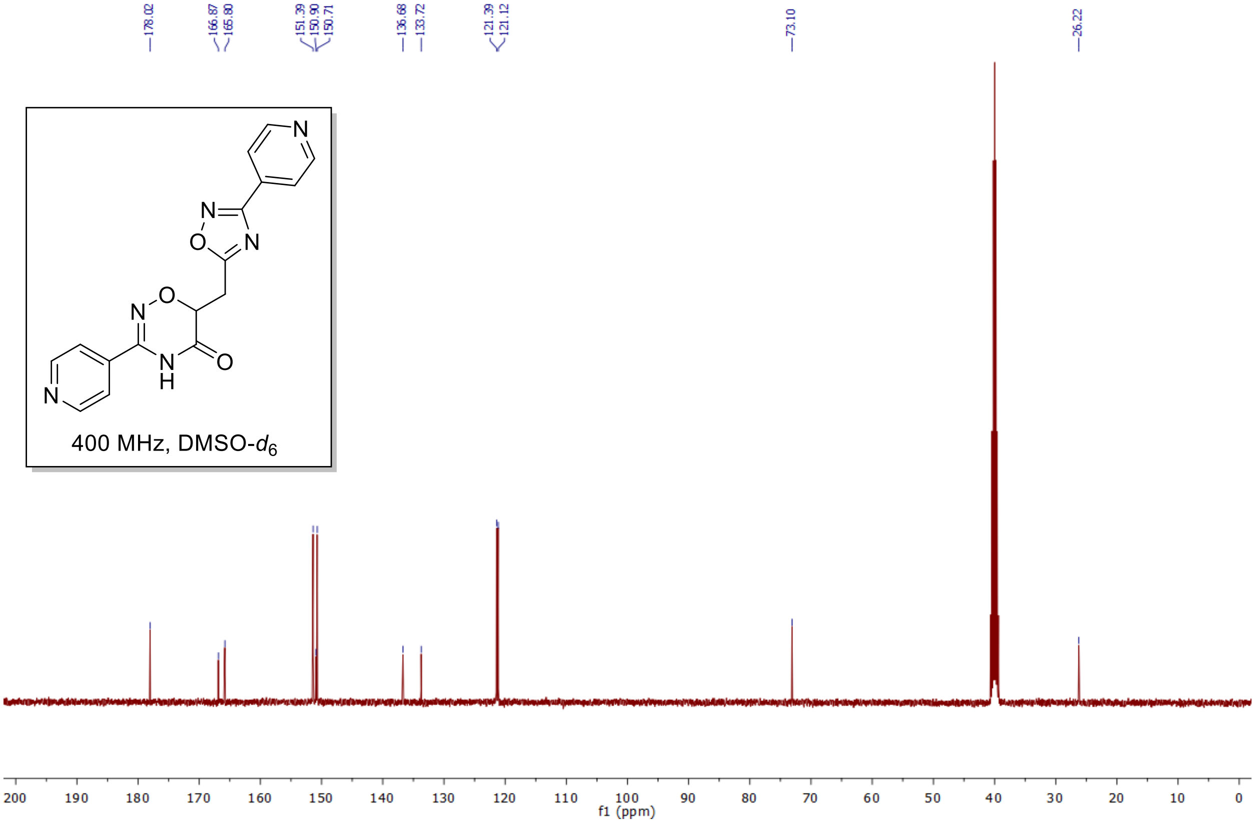

^{1}H and ^{13}C spectra of 3-(pyridin-2-yl)-6-((3-(pyridin-2-yl)-1,2,4-oxadiazol-5-yl)methyl)-4*H*-1,2,4-oxadiazin-5(6*H*)-one **5l**

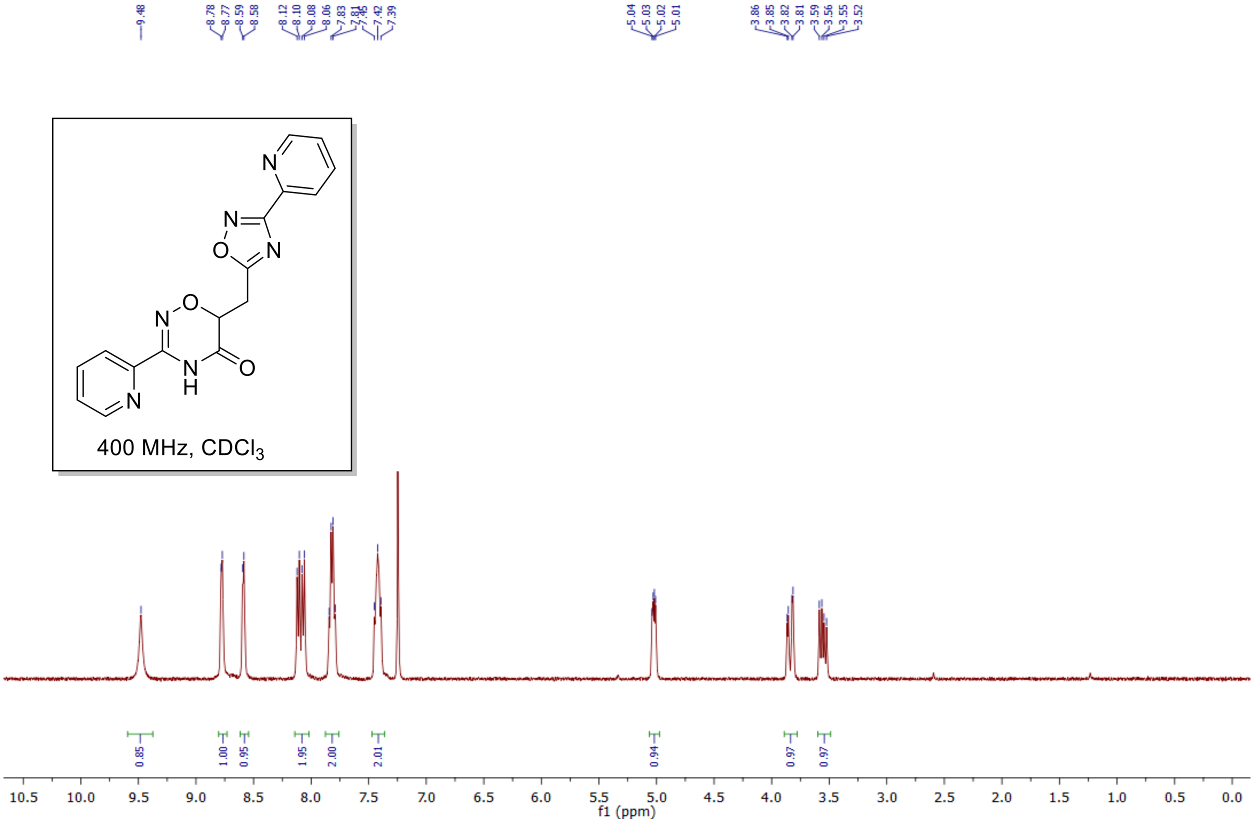

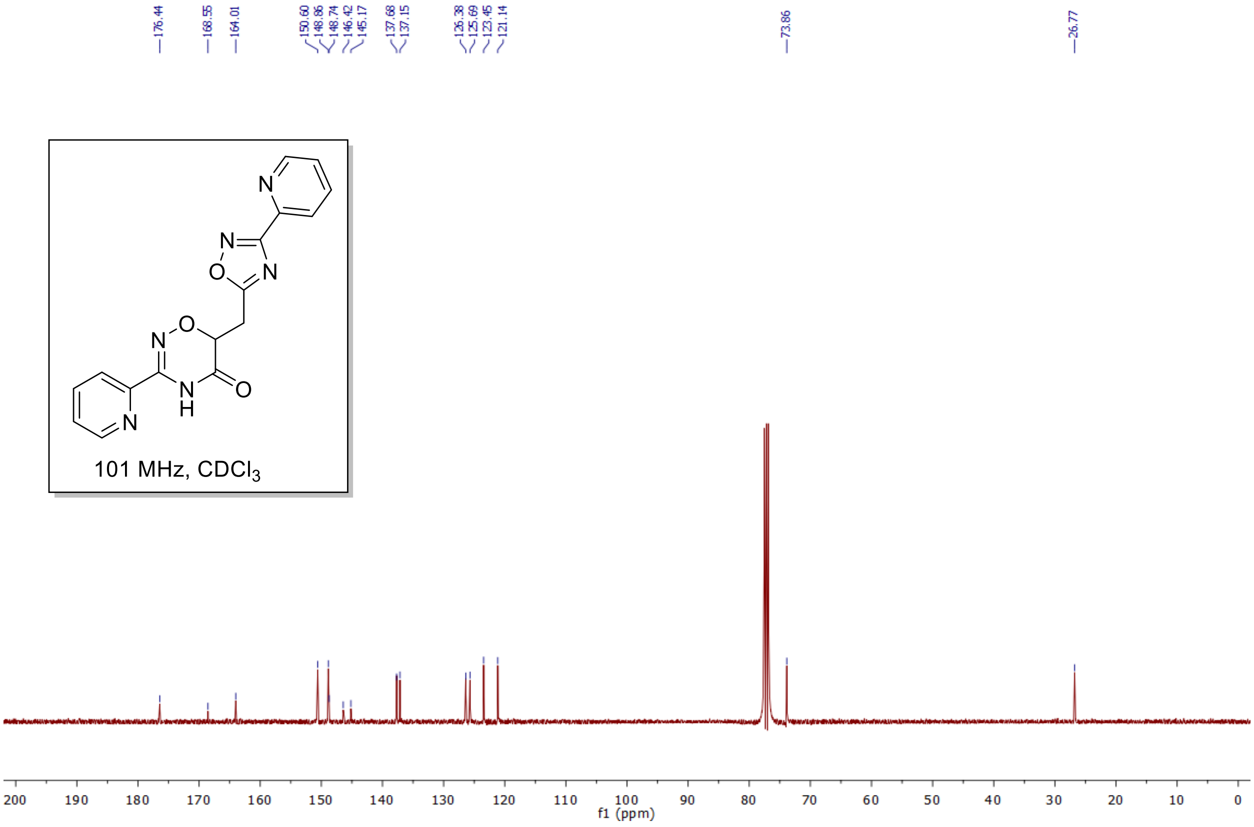

2. X–ray diffraction data

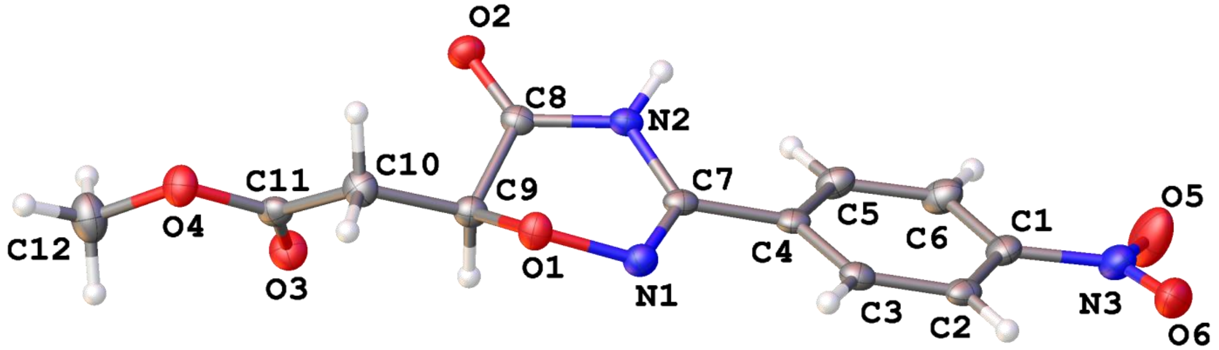

Figure S1. View of the molecular structure of **4d**. Thermal ellipsoids are drawn at the 50% probability level.

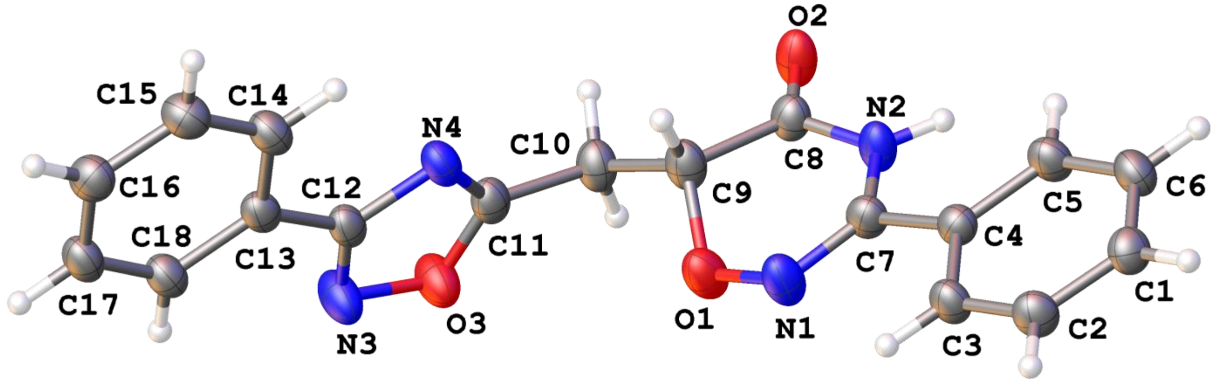

Figure S2. View of the molecular structure of **5b**. Thermal ellipsoids are drawn at the 50% probability level.

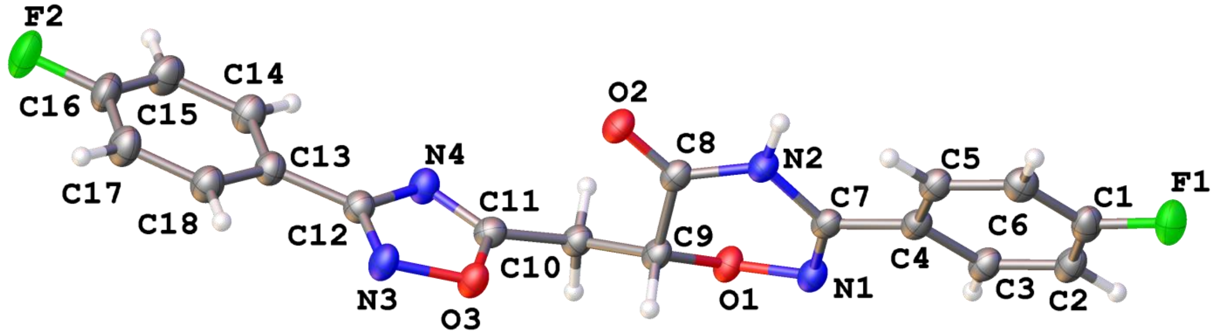

Figure S3. View of the molecular structure of **5f**. Thermal ellipsoids are drawn at the 50% probability level.

Table S1. Crystal data and structure refinement parameters for **4d**, **5b,** and **5f**.

Compound	**4d**	**5b**	**5f**
Identification code	SAV5	OZI-09	CJL-151
CCDC number	2210873	2210875	2210876
Empirical formula	$C_{24}H_{22}N_6O_{12}$	$C_{18}H_{14}N_4O_3$	$C_{18}H_{12}F_2N_4O_3$
Formula weight	586.47	334.33	370.32
Temperature, K	100(2)	100(2)	100(2)
Crystal system	monoclinic	triclinic	triclinic
Space group	C2/c	P-1	P-1
a, Å	21.0394(11)	5.6292(2)	6.2398(2)
b, Å	6.8178(3)	9.9963(3)	7.9297(3)
c, Å	18.9521(10)	14.3394(4)	16.7543(4)
α, °	90	97.054(2)	78.848(2)
β, °	98.851(5)	99.633(3)	80.191(2)
γ, °	90	103.958(3)	89.581(3)
Volume, $Å^3$	2686.2(2)	760.72(4)	801.17(4)
Z	4	2	2
ρ_{calc}g, cm^3	1.450	1.460	1.535
μ, mm^{-1}	0.119	0.850	1.055
F(000)	1216.0	348.0	380.0
Crystal size, mm^3	0.16 × 0.14 × 0.13	0.18 × 0.16 × 0.13	0.15 × 0.14 × 0.12
Radiation	MoKα (λ = 0.71073)	CuKα (λ = 1.54184)	CuKα (λ = 1.54184)
2Θ range for data collection, °	6.288 to 54.998	6.344 to 154.134	5.458 to 140.882
Index ranges	$-27 \le h \le 27$<br>$-8 \le k \le 8$,<br>$-24 \le l \le 24$	$-5 \le h \le 6$,<br>$-12 \le k \le 12$,<br>$-18 \le l \le 17$	$-7 \le h \le 7$,<br>$-9 \le k \le 9$,<br>$-20 \le l \le 20$
Reflections collected	20491	21795	12627
Independent reflections	3015 [R_{int} = 0.0313, R_{sigma} = 0.0188]	3132 [R_{int} = 0.0436 R_{sigma} = 0.0236]	3026 [R_{int} = 0.0397, R_{sigma} = 0.0375]
Data/restraints/parameters	3015/0/191	3132/0/226	3026/0/244
Goodness–of–fit on F^2	1.060	1.077	1.043
Final R indexes [I>=2σ (I)]	R_1 = 0.0472<br>wR_2 = 0.1232	R_1 = 0.0462,<br>wR_2 = 0.1303	R_1 = 0.0401,<br>wR_2 = 0.1085
Final R indexes [all data]	R_1 = 0.0529<br>wR_2 = 0.1274	R_1 = 0.0505,<br>wR_2 = 0.1345	R_1 = 0.0445,<br>wR_2 = 0.1141
Largest diff. peak/hole/ $eÅ^{-3}$	0.47/–0.24	0.85/–0.31	0.24/–0.24
